# Influence of Lytic Polysaccharide Monooxygenase Active Site Segments on Activity and Affinity

**DOI:** 10.3390/ijms20246219

**Published:** 2019-12-10

**Authors:** Christophe V.F.P. Laurent, Peicheng Sun, Stefan Scheiblbrandner, Florian Csarman, Pietro Cannazza, Matthias Frommhagen, Willem J.H. van Berkel, Chris Oostenbrink, Mirjam A. Kabel, Roland Ludwig

**Affiliations:** 1 Biocatalysis and Biosensing Laboratory, Department of Food Science and Technology, BOKU—University of Natural Resources and Life Sciences, Vienna, Muthgasse 18, 1190 Vienna, Austria; christophe.laurent@boku.ac.at (C.V.F.P.L.); stefan.scheiblbrandner@boku.ac.at (S.S.); florian.csarman@boku.ac.at (F.C.); pietro.cannazza@unimi.it (P.C.); 2 Institute of Molecular Modeling and Simulation, Department of Material Sciences and Process Engineering BOKU—University of Natural Resources and Life Sciences, Vienna, Muthgasse 18, 1190 Vienna, Austria; 3 Laboratory of Food Chemistry, Wageningen University & Research, Bornse Weilanden 9, 6708 WG Wageningen, The Netherlands; peicheng.sun@wur.nl (P.S.); matthias.frommhagen@wur.nl (M.F.); willem.vanberkel@wur.nl (W.J.H.v.B.); mirjam.kabel@wur.nl (M.A.K.); 4 Department of Food, Environmental and Nutritional Sciences (DeFENS), Università degli Studi di Milano, Via Mangiagalli 25, 20133 Milano, Italy

**Keywords:** enzyme engineering, lytic polysaccharide monooxygenase, phylogenetic analysis, regioselectivity, substrate binding, substrate specificity

## Abstract

In past years, new lytic polysaccharide monooxygenases (LPMOs) have been discovered as distinct in their substrate specificity. Their unconventional, surface-exposed catalytic sites determine their enzymatic activities, while binding sites govern substrate recognition and regioselectivity. An additional factor influencing activity is the presence or absence of a family 1 carbohydrate binding module (CBM1) connected via a linker to the C-terminus of the LPMO. This study investigates the changes in activity induced by shortening the second active site segment (Seg2) or removing the CBM1 from *Neurospora crassa* LPMO9C. *Nc*LPMO9C and generated variants have been tested on regenerated amorphous cellulose (RAC), carboxymethyl cellulose (CMC) and xyloglucan (XG) using activity assays, conversion experiments and surface plasmon resonance spectroscopy. The absence of CBM1 reduced the binding affinity and activity of *Nc*LPMO9C, but did not affect its regioselectivity. The linker was found important for the thermal stability of *Nc*LPMO9C and the CBM1 is necessary for efficient binding to RAC. Wild-type *Nc*LPMO9C exhibited the highest activity and strongest substrate binding. Shortening of Seg2 greatly reduced the activity on RAC and CMC and completely abolished the activity on XG. This demonstrates that Seg2 is indispensable for substrate recognition and the formation of productive enzyme-substrate complexes.

## 1. Introduction

Lignocellulose is the most abundant renewable resource, but because of the many difficulties to process plant biomass, its huge potential cannot be fully utilized yet. The great number of hydrolytic enzymes found in the secretomes of lignocellulolytic microorganisms indicate the difficulties in efficiently degrading the interwoven biopolymers lignin, cellulose and hemicellulose. In recent years it has been found that specialized oxidoreductases support the action of hydrolases on biopolymers. By performing oxidative cleavage of a polysaccharide chain in the presence of an electron donor and an oxygen species, extracellular fungal lytic polysaccharide monooxygenases (LPMOs) increase the substrate accessibility for, and potentiate the action of, other carbohydrate active enzymes [1,2,3,4,5,6,7,8]. Since the discovery that LPMOs have an oxidative activity on cellulose, there have been constant additions to the various LPMO families. Until now, the number of organisms from different kingdoms reported to produce LPMOs has multiplied and the number of classes or families into which LPMOs are sorted has increased considerably [1]. The classification criteria are either based on the substrate specificity for polysaccharide biopolymers (Enzyme Commission, EC) or on the amino acid sequence, structural features and substrate specificity [carbohydrate active enzyme database (CAZy; www.cazy.org)] [9]. The first two LPMO families created in the CAZy database were classified as auxiliary activities (AA) in families AA9 and AA10. AA9 comprises LPMOs which cleave cellulose chains by oxidation of carbons at C1 (EC 1.14.99.54, lytic cellulose monooxygenase (C1-hydroxylating)) or at C4 carbon atom (EC 1.14.99.56, lytic cellulose monooxygenase (C4-dehydrogenating)). The AA10 family comprises LPMOs that act on chitin (EC 1.14.99.53, lytic chitin monooxygenase) and cellulose (both EC 1.14.99.54 and EC 1.14.99.56) [10]. Within the past years, new LPMO families have been added: AA11 acting on chitin [11], AA13 acting on starch (EC 1.14.99.55 lytic starch monooxygenase) [12], AA14 acting on xylan and cellulose [13], AA15 acting on cellulose and chitin [14] and AA16 acting on cellulose oxidizing the C1 carbon position (EC 1.14.99.54) [15]. The Enzyme Commission has also established a preliminary EC number for lytic xyloglucan monooxygenase (EC 1.14.99.B10).

The catalytic activity of LPMO relies on a mono copper-dependent active site. The copper ion is coordinated by three nitrogen atoms that are part of a histidine-brace composed of two conserved histidine residues [6]. The N-terminal His contributes two copper-binding nitrogen atoms, whereas the third copper-binding nitrogen is part of the second His found in a HXGP motif. This conserved structural motif is found in all LPMOs and is located at the base of the immunoglobin-like β-sandwich fold. In order to initiate the oxidative cleavage of its substrate, the copper center of LPMO needs to undergo a so-called “priming reduction” by either a small molecular reductant or an enzyme like cellobiose dehydrogenase [8]. Subsequently, the breakage of the polysaccharide chains by LPMO is accomplished through an H_2_O_2_ [16] driven mechanism. Despite the ongoing debate of the nature of the co-substrate (O_2_ or H_2_O_2_) [1,5,16], it is agreed that LPMOs oxidize glycosidic bonds through a reactive Cu^2+^−O^•−^ species [17]. Fast oxidative damage of LPMO has been reported when the enzyme is not in contact with its substrate [5,16], which supports the presence of a reactive oxygen species. The disruption of the polysaccharide chain results in an increase of the substrate accessibility and hence potentiates the activity of other carbohydrate active enzymes [1,3,6,8].

LPMOs do not feature a substrate binding pocket, but rather have a flat, slightly grooved binding surface around the exposed active site copper atom [2,18]. However, the exact amino acid residues in this binding surface responsible for substrate recognition and regioselectivity of LPMOs are still unknown. Regioselectivity defines whether LPMOs oxidize glucosyl residues in cellulose at the C1 or C4 position, or both [19]. Studies to link sequences and structural features to the regioselectivity of LPMO have been attempted [19,20,21,22,23,24,25,26,27,28,29,30]. So far, although similarities have occurred, such phylogenetic analysis could not unambiguously predict the three LPMO regioselectivities. Therefore, other studies focused on the deletion of amino acids to study regioselectivity. Two independent studies targeted the so-called L2 loop [20] of two different C1/C4-oxidizing LPMOs to render regioselectivity. Whereas in one case a change in the regioselectivity was reported [19], another study resulted in an inactive enzyme variant [27]. The latter indicates that mutational studies of the LPMO active site are complicated and difficult to compare. Most likely, sequence and structural differences between LPMOs, i.e., from different organisms, are too large to allow good comparison.

In addition to a catalytic module, around one fifth of fungal LPMOs feature a C-terminal CBM1 connected via a flexible linker of variable length and amino acid composition [2,31]. CBM1 is an important module empowering cellulose binding of many hydrolytic cellulose-degrading enzymes [32]. A similar binding role of this CBM1 for LPMOs was demonstrated by isothermal calorimetry (ITC), which measured a forty times lower dissociation constant (*K*_d_) for the complex between the CBM1 of *Nc*LPMO9C and regenerated amorphous cellulose (RAC) than for the complex between the catalytic domain of *Nc*LPMO9C and RAC [24]. Interestingly, removal of the CBM1 from the C1/C4 oxidizing *Podospora anserina* LPMO9H, not only negatively affected binding and activity, but also resulted in a shift in regioselectivity towards predominantly C1 oxidation [32]. Similarly, other studies reported that truncation of the linker and CBM1 of LPMOs affect both the substrate binding and the formed products [24,26].

In the current research, the correlation between AA9 LPMO sequences and activity, substrate binding and regioselectivity is studied. Sequence alignments and phylogenetic analyses were used to define structural elements likely to influence substrate binding. Based on this work, *Nc*LPMO9C was subjected to enzyme engineering to remove a segment of the active site or to remove the CBM1. The performance of the generated *Nc*LPMO9C variants was studied by spectrophotometric activity assays, surface plasmon resonance measurements (SPR) and an HPLC-based product pattern analysis. The results were compared to the corresponding properties of native *Nc*LPMO9C, native *Nc*LPMO9F and native *Nc*LPMO9M, the latter two enzymes having different segments.

## 2. Results

### 2.1. Clustering of AA9 LPMOs Is Based on Extended Catalytic Site Segments

**Figure 1:**
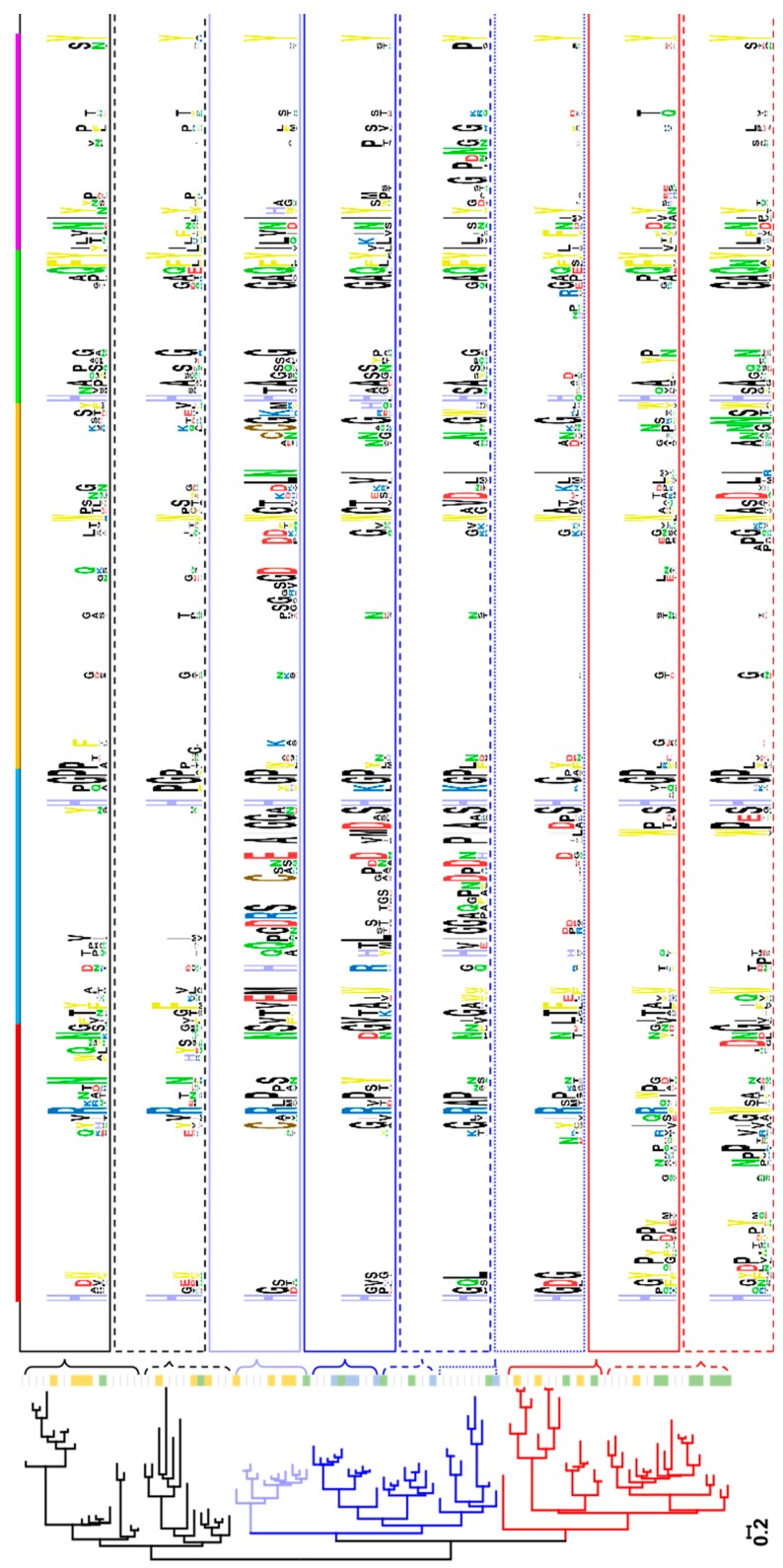
Phylogenetic tree showing the clustering of AA9 lytic polysaccharide monooxygenases (LPMOs) (Cluster 1, black; Cluster 2, blue; Cluster 3, red) and sequence logos of distinct clades within these clusters for the segment sequences. The colored boxes beside the clades indicate the regioselectivity of characterized LPMOs according to Frommhagen et al. [33]: C1, yellow; C4, blue; C1/C4, green. The colored boxes above the sequence logos indicate the extent of each segment: Seg1, red; Seg2, blue; Seg3, orange; Seg4, green; Seg5, purple, and correlate with the color code used in Figure 2.

**Figure 2:**
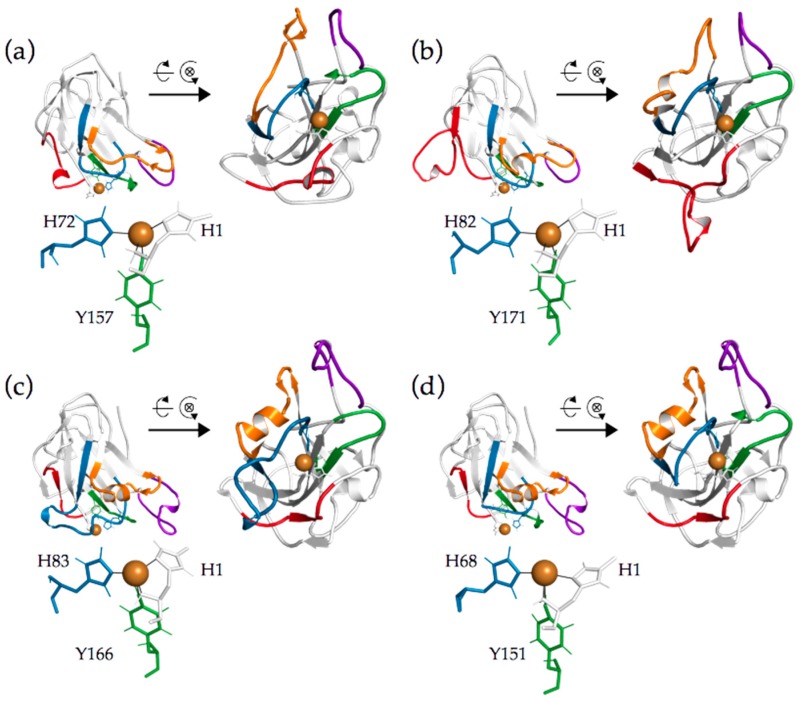
Cartoon representation of (**a**) *Nc*LPMO9F (PDB ID: 4QI8), (**b**) *Nc*LPMO9M (PDB ID:4EIS), (**c**) *Nc*LPMO9C (PDB ID: 4D7U) and (**d**) *Nc*LPMO9C^∆Seg2^. The copper atom in the active site is shown as a brown sphere and its coordinating residues are shown in stick representation. Segments are colored: Seg1, red; Seg2, blue; Seg3, orange; Seg4, green; Seg5, purple. The residues within these segments are listed in Supplementary Table S1. The 3D models have been structurally aligned onto each other and the secondary structure elements were determined using the “Define Secondary Structure of Proteins” (DSSP) algorithm [34] as implemented in GROMOS++ [35]. The model is shown from two directions, the arrow indicates the LPMO model that has been rotated by 90° to show the active site region from the front.

A multiple sequence alignment of 101 putative fungal AA9 LPMO amino acid sequences was compiled with the structure-based MAFFT-DASH algorithm (Supplementary Figure S1). Subsequent phylogenetic analysis was performed with RaxML-NG using the maximum likelihood algorithm (Supplementary Figure S2). The phylogenetic analysis was based on mature protein sequences selected from the CAZy database with a focus on fungi having putative multiple LPMOs. The analyzed sequences grouped into three clusters and LPMOs originating from the same fungus distributed over all three clusters. For example, *Nc*LPMO9F is found in Cluster 1, *Nc*LPMO9C in Cluster 2, and *Nc*LPMO9M in Cluster 3. The main difference between the three clusters was the length of the two different regions located within the first third of the sequence alignment. Similar observations have been made previously and based on structural data these regions, together with two additional ones, were assigned to loops (L2, L3, LS and LC) close to LPMO’s catalytic site [19,20,24,27]. However, since these regions sometimes include secondary structure elements such as α-helices or β-strands, we prefer the term segment instead of loop. Five sequence segments (Seg1–Seg5) were defined based on the alignment and are indicated in various LPMO structures selected from the three clusters (Figure 1, Supplementary Figure S3, Supplementary Table S1). Although the exact designation differs, because our definition is sequence-based, Seg1–3 and Seg5 are comparable to the previously defined L2, L3, LS and LC regions, respectively. Seg4 is short and has not been mentioned in previous literature. LPMOs found in Cluster 1 have a short Seg1 and Seg2 exemplified by *Nc*LPMO9F (Figure 2a). LPMOs in Cluster 2 have an extended Seg2 (*Nc*LPMO9C, Figure 2c), whereas LPMOs in Cluster 3 have an extended Seg1 (*Nc*LPMO9M, Figure 2b). More examples of segments of LPMOs with a resolved structure are shown in Supplementary Figure S4.

### 2.2. Analysis of Active Site Segments Hints Towards Regioselectivity

By analyzing the sequence alignment and the resulting phylogenetic tree we observed that the LPMO clustering roughly reflects the length and composition of the segments. To remove the influence of other positions, a sequence alignment of only the sequences defined as segments was generated (Supplementary Figure S5) and a phylogenetic tree inferred. A comparison of the phylogenetic trees obtained from the alignment of full-length sequences and the “segments only” alignment highlights small differences between these two approaches (Supplementary Figure S2). One difference is the regrouping of a small branch of LPMO sequences containing an elongated Seg2 and a cysteine in Seg1 and Seg3 from Custer 1 to Cluster 2.

This shows that the sequences in the segments affect the clustering in the phylogenetic analysis the most although covering only 33–41% of the total sequence (not including linker or CBM when present), whereas the rest of the positions found in the core and substrate averted surfaces contributes little to the final result. An overview of the amino acids involved in the five segments is given in Supplementary Figure S5. The clusters are relatively well-supported by high bootstrap values, but the phylogenetic distance between the three clusters is relatively small in comparison to the distances between the clades in a cluster. This points to an early acquisition of the differentiating positions (mostly the segments) and an extended evolution of LPMOs within the clusters without changing the segment length. However, the amino acid composition within the segments varies considerably as is obvious from the relatively few conserved positions in the sequence logos in Figure 1. Additionally, most of the conserved positions are at the start and end of the segments and few are found in the middle. The inspection of twelve fungal genomes (*Arthrbotrys oligospora*, *Fusarium fujikuroi*, *F. graminearum*, *F. venenatum*, *Leptosphaeria maculans*, *Neurospora crassa*, *Podospora comata*, *P. anserina*, *Pyricularia oryzae*, *Serendipita indica*, *Thermothelomyces thermophilus*, *Thermothielavioides terrestris*) containing 10 or more *lpmo* genes showed that every fungus in this group encodes LPMOs from all three clusters. This supports the important role of the segments as a basis for the evolution of LPMOs.

When adding the published regioselectivities of the used LPMOs [33] no straightforward correlation of the preferred cleavage position and the clustering was obtained, although certain trends can be found for these three clusters. In Cluster 3, featuring an elongated Seg1, C1/C4-oxidizing LPMOs are predominant (9) together with a small subset of C1-oxidizing LPMOs (4). In Cluster 1, with neither Seg1 or Seg2 elongated, both C1- (7) and C1/C4-oxidizing (2) LPMOs are present, however, here the number of C1-oxidizing LPMOs is higher. In Cluster 2, featuring an elongated Seg2, C4-oxidizing LPMOs are dominant (7) with a small number of C1/C4-oxidizing LPMOs (3). Certainly, the small number of sequences with reported data on the regioselectivity and substrate specificity reduce the predictive power of such an analysis, but the distorted distribution supports the hypothesis that the evolution of sequences within the clusters went a long way after the elongation of the segments and led to a wide diversification of the regioselectivity. The even more interesting question of how the segments and their evolution influences the substrate specificity can, unfortunately, not be answered at present, due to a lack of published substrate specificities for characterized LPMOs.

An interesting observation in regard to Cluster 2 is that in some sequences a cysteine is present in Seg 2. LPMOs with this cysteine residue have a predominantly C1-oxidizing regioselectivity and would in this regard better fit to Cluster 1 than to Cluster 2. Homology models suggest that this cysteine can form a disulfide bond with another cysteine in Seg3 (Supplementary Figure S6). When this bond is present it pulls the elongated Seg2 away from the catalytic site and mimics a short Seg2 that is found in Cluster 1, which contains predominantly C1-oxidizing LPMOs. This can be viewed as an example of evolutionary adaption within a cluster. 

To test the cluster-function relationship (e.g., a potential relationship between Seg2 and a C4 regioselectivity) and the role of the CBM1, we generated a variant of *Nc*LPMO9C found in Cluster 2 and replaced the sequence of Seg2 with that of Seg2 from *Nc*LPMO9F. With that, the LPMO^∆Seg2^ variant was created which is more related to LPMOs from Cluster 1. In addition, we also removed the C-terminal linker and CBM1 from the wild-type *Nc*LPMO9C and from the *Nc*LPMO^∆Seg2^ variant resulting in *Nc*LPMO9C^∆CBM^ and *Nc*LPMO9C^∆Seg2, ∆CBM^, respectively. In detail, *Nc*LPMO9C^∆Seg2^ was generated by replacing the residues 63–82 of *Nc*LPMO9C with residues 67–71 of *Nc*LPMO9F. Rather than deleting the indicated residues from *Nc*LPMO9C we opted for a replacement to avoid a structural strain on the histidine brace forming the active site. Furthermore, Seg2 of *Nc*LPMO9C contains a short β-strand which is not present in *Nc*LPMO9F (Supplementary Figure S3). A secondary structure analysis revealed that this short β-strand should not be present in *Nc*LPMO9C^∆Seg2^. Additionally, we truncated the linker including the CBM1 from both *Nc*LPMO9C and *Nc*LPMO9C^∆Seg2^ by inserting a stop codon at positions P228 and P213, respectively, which resulted in variants *Nc*LPMO9C^∆CBM^ and *Nc*LPMO9C^∆Seg2, ∆CBM^. Additionally, *Nc*LPMO9C, *Nc*LPMO9F and *Nc*LPMO9M were produced for reference experiments.

The activity of the recombinantly expressed and chromatographically purified *Nc*LPMOs and *Nc*LPMO9C variants (Supplementary Figure S7, Supplementary Table S2) was tested with a spectrophotometric assay measuring LPMO activity with 2,6-dimethoxyphenol (2,6-DMP) and H_2_O_2_ [36]. Steady-state kinetic constants of LPMOs and LPMO variants for H_2_O_2_ were determined at pH 6.0 in a sodium acetate buffer (Table 1, Supplementary Figure S8). Based on this assay, all enzymes and variants were active, which indicates that the catalytic site is functional in all produced LPMOs. The measured specific activities of *Nc*LPMO9C and its variants diverge by a factor of three indicating minor effects of the introduced mutations on the catalytic site. The shortened Seg2 in *Nc*LPMO9C^∆Seg2^ and *Nc*LPMO9C^∆Seg2, ∆CBM^ lowers the catalytic efficiency by a factor of two. Removal of the CBM in *Nc*LPMO9C ^∆CBM^ and *Nc*LPMO9C^∆Seg2, ∆CBM^ leads to only small deviations in the catalytic efficiency from the corresponding wild-type and variant enzymes. *Nc*LPMO9M also showed activity with kinetic constants for H_2_O_2_ similar to *Nc*LPMO9C. *Nc*LPMO9F was previously shown to have a specific activity of 2.2 ± 0.2 U g^−1^ in a 100 mM sodium succinate/phosphate buffer at pH 7.5 [36]. The amino acid sequence of all produced LPMOs and LPMO variants was confirmed by liquid chromatography-electrospray ionization mass spectrometry (LC-ESI-MS, Supplementary Figure S9). 

### 2.3. Thermal Stability of Purified LPMOs

**Figure 3:**
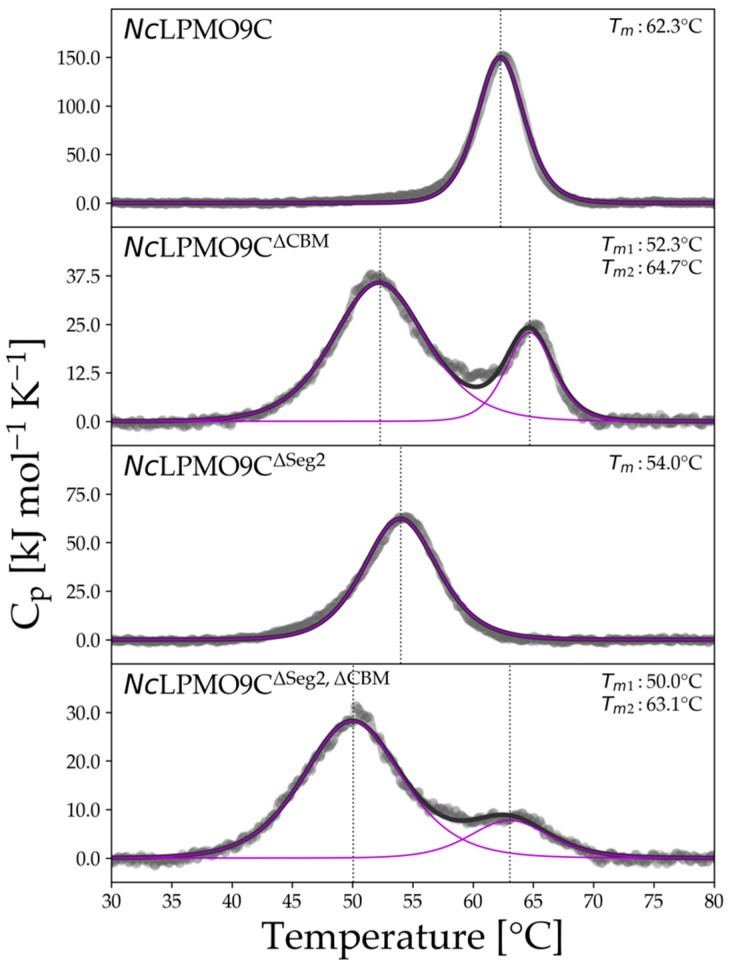
DSC thermograms of *Nc*LPMO and variants. The *Nc*LPMO9C variants lacking linker and a family 1 carbohydrate binding module (CBM1) are designated by ∆CBM and variants with a shortened Seg2 and indicated by ∆Seg2. Solid black lines show the fitted curves to the raw data (grey scatter plots). The transition midpoint temperature (*T_m_*) is indicated by a dotted line.

The thermal stability of *Nc*LPMOs and variants was measured to ensure their proper functioning during further assays. Differential scanning calorimetry (DSC) was used to observe endothermic transitions. *Nc*LPMO9C and *Nc*LPMO9M showed sharp transitions and a similar transition midpoint temperature (*T_m_*) of 62.3 and 63.1 °C, respectively (Figure 3, Supplementary Figures S10 and S11). In contrast, the thermogram of *Nc*LPMO9F revealed a much broader transition with a shoulder at 57.9 °C (*T_m_*_1_) and a maximum at 65.8 °C (*T_m_*_2_) (Supplementary Figure S12). The *T_m_* of *Nc*LPMO9C and *T_m_*_2_ of *Nc*LPMO9F were close to previously reported values [37]. The highest *T_m_* was found for *Nc*LPMO9M which has an additional third disulfide bridge (Supplementary Figure S3).

As a result of shortening Seg2 in *Nc*LPMO9C^∆Seg2^, a decreased *T_m_* was observed and a broader transition compared to the wild-type, indicating a destabilization of the protein fold (Figure 3, Supplementary Figure S13). The removal of the linker and CBM1 in *Nc*LPMO9C^∆CBM^ and *Nc*LPMO9C^∆Seg2, ∆CBM^ resulted in two partially separated, broadened endothermic transitions (Supplementary Figures S14 and S15). Most of the unfolding occurred in the *T_m_*_1_ region, representing 75% and 82% of the total enthalpy, respectively. Whereas the *T_m_*_2_ of both truncated variants was close to the *T_m_* of the wild-type, destabilization of the protein fold by the shortening of Seg2 and removal of the CBM1 was evident. The *T_m_* or *T_m_*_1_ of *Nc*LPMO9C^∆Seg2^, *Nc*LPMO9C^∆CBM^, and *Nc*LPMO9C^∆Seg2, ∆CBM^ decreased in relation to the wild-type by 8.3, 10, and 12.3 °C, respectively. 

**Figure 4:**
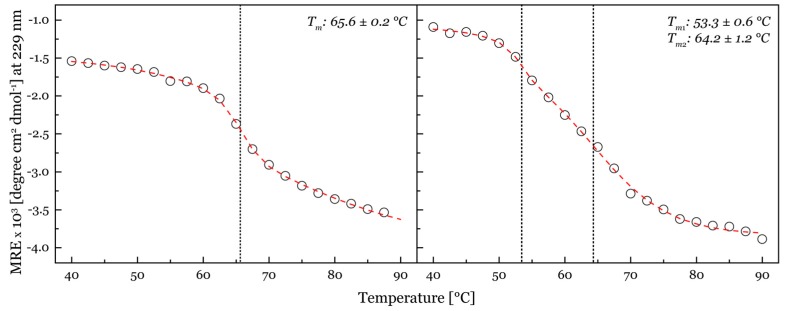
Thermal unfolding followed by circular dichroism spectroscopy. A double sigmoidal curve was fitted to the changes of ellipticity at 229 nm to calculate the transition midpoint temperatures of 3 mg mL^−1^ *Nc*LPMO9C (left panel) and 3 mg mL^−1^ *Nc*LPMO9C^ΔCBM^ (right panel). (Mean residue ellipticity, MRE).

An explanation for the strong destabilizing effect of the removal of the linker and CBM1 on the thermal stability of the resulting variants is that the termination site of the linker is directly after a cysteine. Subsequent linker residues might be involved in the correct folding of the protein and the formation of the C-terminal disulfide bridge in *Nc*LPMO9C (Supplementary Figure S3). The truncation directly after this cysteine could hinder the formation of this disulfide bridge. Support for this hypothesis comes from the presence of *T_m_*_2_ in *Nc*LPMO9C^∆CBM^ and *Nc*LPMO9C^∆Seg2, ∆CBM^, which likely represents the smaller fraction of LPMO with a disulfide bridge. To proof this hypothesis, we incubated LPMOs with 5 mM tris(2-carboxyethyl)phosphine (TCEP) before a DSC scan. Unfortunately, the addition of TCEP reduced all disulfide bonds in the LPMOs, which resulted in thermograms that barely differed from the buffer signal, but demonstrate the important role of disulfide bonds to maintain the native protein conformation. Circular dichroism spectroscopy was used to verify DSC experiments and obtain insights into the changes of secondary structure elements during unfolding (Figure 4). The change in ellipticity at 229 nm indicates also one *T_m_* at 65.6 °C for *Nc*LPMO9C and two *T_m_* values for *Nc*LPMO9C^∆CBM^ and thereby support the DSC experiments. The temperature-induced change in ellipticity between 40–90 °C (Supplementary Figure S16) shows that the unfolding of secondary structure elements begins approximately 5 °C below the *T_m_* for both *Nc*LPMO9C and *Nc*LPMO9C^∆CBM^. Antiparallel β-sheets are the main secondary structure element found in LPMO. The extent of unfolding of the antiparallel β-sheets is similar for both enzymes indicating the same secondary structure composition, but starts already at a lower temperature for *Nc*LPMO9C^∆CBM^ (~50 °C) than for *Nc*LPMO9C (~60 °C). A possible explanation is again the lower stability of the *Nc*LPMO9C^∆CBM^ fraction that has no intact, second disulfide bond in the catalytic domain. Highly interesting is the partial refolding of LPMO, which we observed in DSC rescans (Supplementary Figures S10–S15). The ratio of ${\Delta H}_{\mathrm{cal}}^{\mathrm{rescan}}$ over ${\Delta H}_{\mathrm{cal}}^{\mathrm{scan}}$ was very low for *Nc*LPMO9M (0.07), but moderate for *Nc*LPMO9F (0.38) indicating that the unfolding process was not fully reversible (Supplementary Figure S17). However, *Nc*LPMO9C had a ratio of 0.78 which is close to the threshold classifying reversible unfolding [38]. The removal of Seg2 and the CBM1 decreased this ratio (again additively), indicating their stabilizing role for the protein fold.

### 2.4. LPMO Activity on Cellulosic Substrates

**Figure 5:**
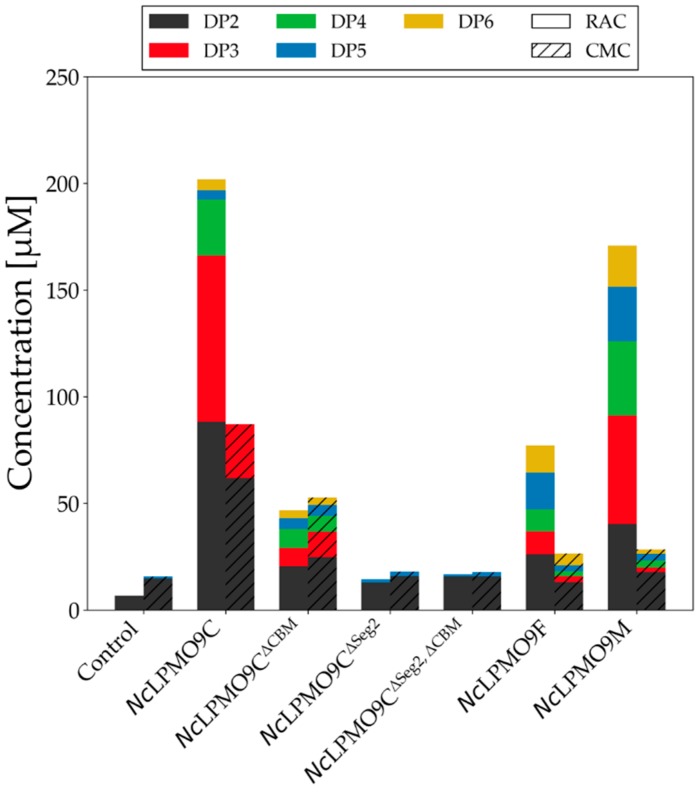
Quantification of released soluble cellodextrins from the incubation of 2 mg mL^−1^ RAC or carboxymethyl cellulose (CMC) with 1.25 µM *Nc*LPMOs and variants in the presence of 1 mM ascorbic acid after 24 h. The quantification of soluble cellodextrins and the calculation is defined in Supplementary Table S3. To exclude the impact of hydrolytic background activity, soluble cellodextrins that were released from the incubation of RAC or CMC with *Nc*LPMOs in the absence of ascorbic acid were subtracted from the above-mentioned quantified values (Supplementary Figure S22). Buffer was added to the control reactions instead of LPMO. DP: degree of polymerization.

To test the enzymatic activity of the produced LPMOs and their variants on cellulosic substrates, all were incubated with RAC for 24 h at 30 °C in a constantly rotating orbital shaker. In agreement with their classifications, the use of high-performance anion exchange chromatography (HPAEC) showed that *Nc*LPMO9C released C4-oxidized products and *Nc*LPMO9F released C1-oxidized products, next to non-oxidized products (Supplementary Figures S18 and S19). RAC incubated with *Nc*LPMO9M led to the release of the previously described C1- and C4-oxidized and non-oxidized products. Oxidized oligosaccharides were identified based on elution time of previously developed methods [39,40,41]. The same regioselectivity was observed when the soluble substrate carboxymethyl cellulose was incubated with the LPMOs (CMC, Supplementary Figures S20 and S21). CMC incubations with *Nc*LPMO9C, *Nc*LPMO9F, and *Nc*LPMO9M resulted in lower amounts of oxidized products, more specifically cellodextrins (degree of polymerization (DP) ranging from 2 to 6) compared to corresponding RAC incubations. (Figure 5, Supplementary Figure S22, Supplementary Tables S4 and S5). Notably, *Nc*LPMO9M and *Nc*LPMO9F released considerably less amounts of non-oxidized and oxidized products from CMC compared to *Nc*LPMO9C.

The removal of the CBM1 in *Nc*LPMO9C^∆CBM^ resulted in a decreased activity towards RAC compared to the wild-type (Figure 5). The total release of soluble non-oxidized products by *Nc*LPMO9C^∆CBM^ was 23% for RAC and 60% for CMC compared to *Nc*LPMO9C (set as 100%). This decrease in the release of non-oxidized (Figure 5) and oxidized (Supplementary Figures S18 and S20) oligosaccharides shows that the CBM1 contributes to the substrate recognition and binding as prerequisite of catalysis. This is especially true for RAC. Interestingly, the preferential conversion of RAC over CMC by *Nc*LPMO9C was not found for *Nc*LPMO9C^∆CBM^. Based on the amounts of the released products one could argue that the removal of the CBM1 no longer allows the enzyme to distinguish between RAC and CMC. Unfortunately, influence of the modified Seg2 on the LPMO substrate specificity and regioselectivity could not be evaluated due to the low activity of *Nc*LPMO9C^∆Seg2^ and *Nc*LPMO9C^∆Seg2, ∆CBM^. Nevertheless, the low activity of *Nc*LPMO9C^∆Seg2^ and *Nc*LPMO9C^∆Seg2, ∆CBM^ indicates that Seg2 is a crucial component for substrate recognition in this Cluster 2 LPMO.

### 2.5. LPMO Activity on Xyloglucan

Since *Nc*LPMO9C is active on tamarind seed xyloglucan (XG) [42], we were interested in how the *Nc*LPMO9C variants performed on this soluble hemicellulose under quasi-homogeneous conditions. A release of non-oxidized and oxidized xylogluco (XG)-oligosaccharides from XG in presence of reduced *Nc*LPMO9C was observed (Supplementary Figures S23 and S24). Similar to the results with RAC and CMC, the amount of non-oxidized and oxidized XG-oligomers released by *Nc*LPMO9C^∆CBM^ was reduced compared to its wild-type. A qualitative difference of oxidized XG-oligomers was observed for peaks with an elution time between 25–45 min between *Nc*LPMO9C^∆CBM^ and *Nc*LPMO9C. No substantial differences of released XG-oligomers of the control and *Nc*LPMO9C^∆Seg2^ or *Nc*LPMO9C^∆Seg2, ∆CBM^ were found, showing that the Seg2 variants lost the capacity of oxidatively degrading XG. The molecular weight distribution (hydrodynamic volume) of XG incubated with the LPMOs was analyzed over time by using high-performance size exclusion chromatography using a refractive index detector (HPSEC-RI). In the absence or presence of ascorbic acid, XG incubated without LPMOs had a molecular weight distribution in the range between 20 kDa to above 700 kDa (Supplementary Figures S25 and S26). With the incubation of XG with *Nc*LPMO9C or *Nc*LPMO9C^∆CBM^ in the presence of ascorbic acid_,_ the hydrodynamic volume of XG decreased dramatically over time leading to the formation of XG-oligomers with a molecular weight distribution between 20 kDa to 0.4 kDa (Supplementary Figures S27–S30).

For a better overview of the product size distribution over time, three elution windows were defined for integration (elution times defined in Supplementary Table S6). Window 1 at the beginning of the elution covers the biggest XG molecules with a molecular weight between 21.2 and >1000 kDa. Window 2 includes middle-sized XG molecules with a molecular mass range of 7–21.2 kDa and Window 3 represents small-sized reaction products with a molecular mass from 0.35–7 kDa (Supplementary Figures S26, S28 and S30). Remarkably, the presence of ascorbic acid had an immediate, molecular-mass decreasing effect in Window 1 (Supplementary Figure S25), which was not observed in the absence of ascorbic acid (Supplementary Figure S25, S27 and S29). 

**Figure 6:**
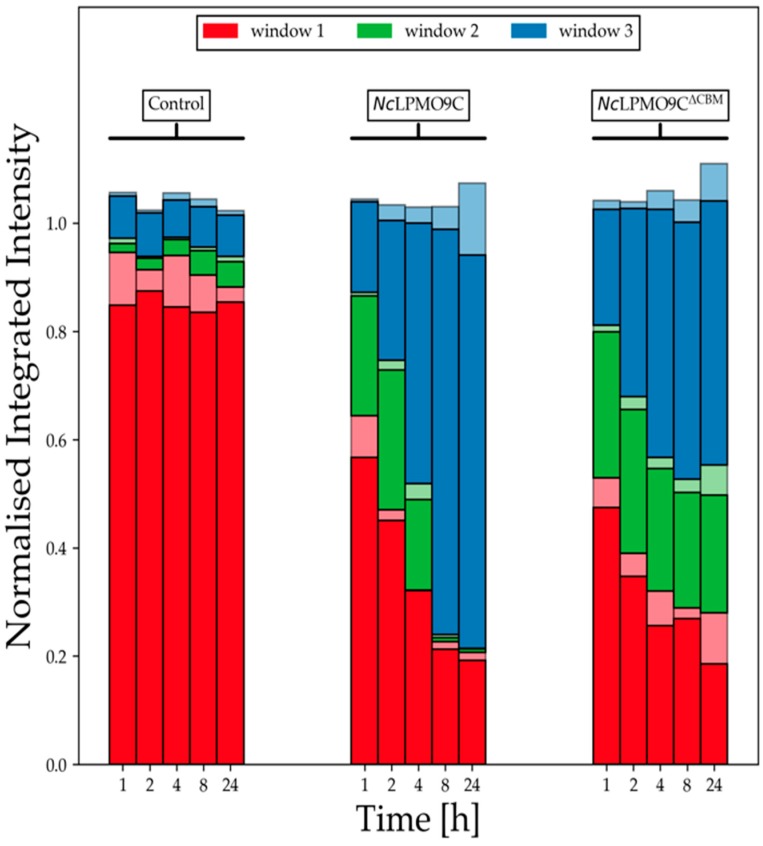
Normalized integrated intensity of high performance size exclusion chromatography coupled to a refractive index detector (HPSEC-RI) chromatograms over time. Integration windows 1, 2 and 3 were defined as ranging from 7.4–11.0, 11.0–12.0, and 12.00–14.75 min (Supplementary Table S6). The upper boundaries of the windows were excluded from the integration. Data for two replicates are shown and have been normalized to the sum of the average data points per time point. The opaque bars and the bars with a reduced transparency are indicating the minimum values and maximum values from two independent measurements.

Figure 6 (reference experiments in Supplementary Figure S31) shows the normalized, integrated intensities of the above-described windows. In general, *Nc*LPMO9C and *Nc*LPMO9C^∆CBM^ quickly reduced the amount of large-size XG molecules found in Window 1 and accumulated mid-sized XG molecules in Window 2, which were then further converted to the even smaller, final XG products found in Window 3. *Nc*LPMO9C consumed the mid-sized XG molecules fully after 4 h, whereas *Nc*LPMO9C^∆CBM^ stopped after 4 h to convert mid-sized XG molecules into small-sized final products indicating a stop of the conversion process. Fitting areas of Window 3 to an exponential function (Supplementary Figure S32), one observes after only 4 h a plateau for *Nc*LPMO9C^∆CBM^ whereas *Nc*LPMO9C continues to increase until 8 h. According to the linear slope between 0–4 h, the production rate of small-sized XG molecules is similar for *Nc*LPMO9C and *Nc*LPMO9C^∆CBM^ and shows the CBM1 is not essential for the activity on XG. However, the CBM1 might increase the binding affinity to XG to avoid self-inactivation of LPMO catalytic site, leading to a longer reaction time. Furthermore, the lack of activity of *Nc*LPMO9C^∆Seg2^ and *Nc*LPMO9C^∆Seg2, ∆CBM^ on XG indicates that Seg2 is also important for the substrate recognition of XG.

### 2.6. LPMO Binding to Cellulose

**Figure 7:**
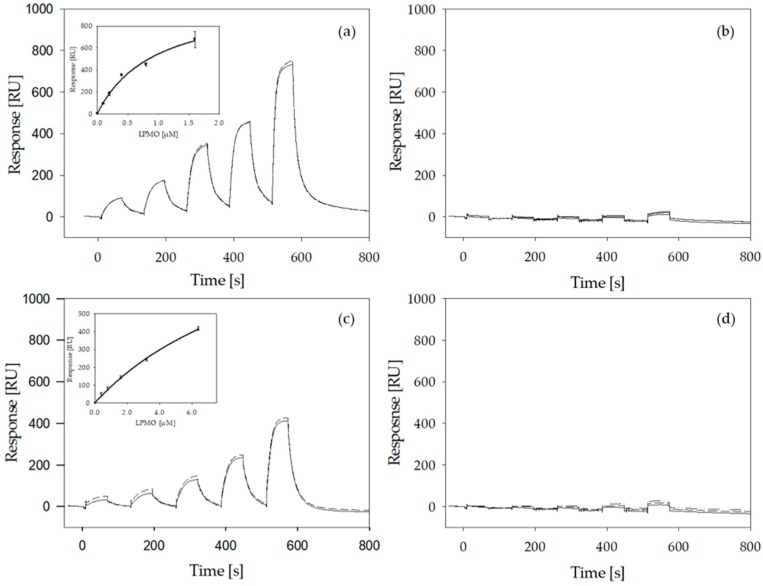
Sensograms of surface plasmon resonance measurements (SPR) interaction studies of LPMO on gold-modified cellulose surfaces measured by single-cycle kinetics. Five injections with increasing concentrations of the analyte were performed. (**a**) *Nc*LPMO9C (measured concentration: 0.1, 0.2, 0.4, 0.8 and 1.6 µM), (**b**) *Nc*LPMO9C^∆CBM^, (**c**) *Nc*LPMO9C^∆Seg2^, (**d**) *Nc*LPMO9C^∆Seg2, ∆CBM^ (all with concentrations of: 0.4, 0.8, 1.6, 3.2 and 6.4 µM). All runs were performed in triplicates. Insets in (**a**) and (**c**) show LPMO concentration vs. response units and were fitted with SigmaPlot 12.

Conversion experiments clearly showed the correlation between the ability of LPMO to bind to its substrate and the exerted activity. To investigate the effect of the introduced changes on the substrate binding affinity of *Nc*LPMO9C and its variants, *K*_d_ values were determined by SPR measurements (Figure 7). Microcrystalline cellulose was dissolved in *N*,*N*-dimethylacetamide and LiCl and spin-coated onto the SPR gold probes to produce a thin layer of cellulose. By measuring the amount of adsorbed LPMO using single-cycle-kinetic measurements with five sequential LPMO injections, *K*_d_ values were calculated for *Nc*LPMO9C and its variants (Figure 7). Kinetic titration experiments were chosen over multicycle experiments to avoid the modification of the deposited cellulose film by surfactants or high ion concentrations. The *K*_d_ determined for the complex between *Nc*LPMO9C and the deposited cellulose was 0.94 ± 0.27 µM, an about 10-times higher affinity than estimated with *Nc*LPMO9C^ΔSeg2^ (*K*_d_ = 11.7 ± 2.76 µM). This showed clearly that the binding site of *Nc*LPMO9C^ΔSeg2^ has a greatly decreased substrate affinity and it also showed the synergistic effect of the substrate binding site and the CBM1. The two other studied wild-type *Nc*LPMOs have no CBM1, which allowed us to study the affinity of a single LPMO binding site (although with different active site segments). By applying the SPR methodology described above we determined for the Cluster 1 *Nc*LPMO9F a *K*_d_ of 25.6 ± 6.87 µM and for Cluster 3 *Nc*LPMO9M a *K*_d_ of 53.3 ± 7.87 µM for the complex with the cellulose substrate (Figure 7). Keeping in mind that the cellulose on the SPR probe is only an approximation of its naturally available form, it still demonstrated that the presence of a CBM1 directs the LPMO to its substrate and decreases the fraction of unbound, diffusible LPMO.

To quantify how much the removal of the CBM1 and the shortening of Seg2 decreased the affinity we tried to determine the *K*_d_ values for *Nc*LPMO9C^∆CBM^ and *Nc*LPMO9C^∆Seg2, ∆CBM^, but did observe only very weak binding (Figure 7b,d). Unfortunately, this did not allow us to perform reliable calculations. This demonstrates that the binding site of *Nc*LPMO9C, neither intact nor with the shortened Seg2, has a high affinity towards the employed cellulosic substrate. We can therefore only indirectly deduce that the loss of activity observed for *Nc*LPMO9C^∆Seg2^ (Figure 5) comes from a reduced affinity of the binding site, because *Nc*LPMO9C^∆CBM^ is active on RAC, CMC, and XG.

Investigating the binding behavior of LPMO in its reduced state to its cellulosic substrate by SPR measurements, using 10 mM gallic acid as a reducing agent, showed no significant difference of the binding affinity for *Nc*LPMO9C compared to the oxidized form (Supplementary Figure S33). For *Nc*LPMO9C^∆CBM^, a higher response could be observed in its reduced state. Unfortunately, the determination of the *K*_d_ values in the reduced state was not possible. This would require an oxygen-free environment, which unfortunately is a major hurdle in SPR measurements. Moreover, less efficient regeneration of the cellulosic surface in the presence of gallic acid did also hinder reliable experiments.

## 3. Discussion

Previous studies to infer the regioselectivity of LPMOs from phylogenetic trees [19,21,22,24,29,30] resulted in the currently described four types of LPMOs (types 1, 2, 3, and 3*). The amino acid segments around the LPMO catalytic site are flexible which gives them the necessary mobility to adapt to different substrates (Supplementary Figure S34). Intriguingly, this is not changing the site-specific oxidation of the substrate. Type 1 and type 2 LPMOs both hold a short Seg1 and are supposed to have a C1 or C4 regioselectivity, respectively. The elongated Seg2 in type 2 LPMOs has been suggested to be responsible for the preference in C4 regioselectivity [19]. Type 3 LPMOs were found to have an elongated Seg1 and to produce both 4-ketoaldoses and aldonic acids. However, type 3* LPMOs are defined as a subgroup of type 3, solely producing C1-oxidized products [19]. This was attributed to the lack of conserved residues within Seg1. Based on our sequence alignment we found that not all C1-oxidizing LPMOs in Cluster 3 were lacking the conserved residues described by Vu and coworkers (i.e., CAP71839.1 *T. anserina* S mat+ LPMO9F, CAP66744.1 *T. anserina* S mat+ LPMO9D) [19]. Therefore, we suggest that the classification of LPMO regioselectivity solely based on phylogenetic clusters is not reliable enough. One example are LPMOs within Cluster 2 that are featuring a cysteine in Seg2 (i.e., AON76800.1 *T. thermophilus* LPMO9B). A potential disulfide bond to a cysteine in Seg3 could result in a simulated short Seg2 (Supplementary Figure S6). Furthermore, according to Bey and coworkers [43], *T. anserina* S mat+ LPMO9B (CAP68375.1) is switching between the generation of C1/C4- and C1-oxidation products when reduced by ascorbic acid or cellobiose dehydrogenase (CDH), respectively. Since CDH is a natural reductant we suggest that the latter LPMO should belong to type 1 LPMOs rather than type 2. This is highlighting the complexity of the regioselectivity in LPMOs and hinting that the heterogenous distribution might be due to non-optimal experimental conditions. The large phylogenetic distances between LPMO sequences within a cluster shows that the evolutionary adaption process was done after the division into clusters. This leads to highly diverse substrate specificities and regioselectivities. In contrast, the phylogenetic distances between the three clusters are relatively short. Nevertheless, more reliable data from characterized LPMOs are necessary to provide a final proof of the correlation between LPMO phylogeny and regioselectivity.

A previous study showed that it is possible to alter the LPMO regioselectivity by altering Seg1 of the active site [19]. A second study did not reproduce this result for a different LPMO (*Hj*LPMO9A) [27] but resulted in an inactive enzyme. Presumably the loss of activity in the second study was linked to a high strain on the catalytic site after the deletion of Seg1. For this reason, we did not simply shorten Seg2 from *Nc*LPMO9C but replaced it with Seg2 from *Nc*LPMO9F. Despite this strategy, the expressed variant *Nc*LPMO9C^∆Seg2^ showed no activity or affinity towards any of the tested polysaccharide substrates. Since Seg2 is close to the catalytic site, its shortening could influence the integrity of the catalytic site as reported by Danneels and coworkers [27]. However, shortening Seg2 in *Nc*LPMO9C did not result in the complete inactivation of the catalytic activity as demonstrated by the ability to determine the kinetic constants of LPMO variants for H_2_O_2_. The obtained *K_M_*- and *k_cat_*-values of *Nc*LPMO9C and the variant LPMOs were similar to previously published values [36]. In contrast to the recently published work by Chalak and coworkers [32], we were able to successfully produce an active LPMO variant with a truncated linker and CBM1 right after the catalytic domain. However, this modification resulted in a partial reduction of the thermostability. It seems that a truncation right after the cysteine 227 of *Nc*LPMO9C is leading to an unequal folding of the protein. This is supported by the appearance of two distinct *Tm* values in DSC experiments. Most likely the LPMO linker is contributing to the correct folding of the LPMO and formation of the C-terminal disulfide bond back to the catalytic domain of *Nc*LPMO9C. Tanghe and coworkers have shown that disulfide bridges increase the thermal stability of a bacterial LPMO [44]. The observed partial refolding of LPMOs has been previously reported [45,46]. It was suggested that this effect is connected to the disulfide bonds [45]. Moreover, a similar signal collapse near 205 nm in the *Nc*LPMO9C CD spectrum was observed by Frommhagen and coworkers for two *Myceliophthora thermophila* LPMOs [47]. In their study the changes in ellipticity were suggested to show that the LPMOs adopted a fibrillar-like state during heat treatment. It is known that a significantly negative spectrum around 200 nm is pointing towards a poly (L-proline) type II helix [48]. The observed more negative ellipticity above 70 °C in the ECD spectra (Figure 4) therefore suggests that there is no complete unfolding, but possibly a formation of fibrils.

When looking at the regioselectivity on cellulosic substrates, *Nc*LPMOs 9F, 9C and 9M share the same product pattern as previously published [19]. In contrast to Chalak and coworkers [32] no change in the regioselectivity of the enzyme was observed when removing the CBM1. However, recent efforts to elucidate the role of a family 2 CBM linked to LPMO10C from *Streptomyces coelicolor* revealed no change in regioselectivity upon truncation of the CBM2 from the enzyme [26]. Furthermore, the same study also showed that the quantity of formed products was decreased. This finding is further supported by our conversion experiments with *Nc*LPMO9C^∆CBM^. Since *Nc*LPMO9C^∆CBM^ is lacking the CBM1 it is conceivable that the latter domain helps the enzyme to stay close to the substrate and therefore promotes the formation of even smaller products. This effect has been described by Courtade et al. [26]. For the conversion of XG, the presence of the CBM1 is not necessary since similar conversion rates of *Nc*LPMO9C and *Nc*LPMO9C^∆CBM^ were observed. However, the conversion of XG stopped earlier when employing *Nc*LPMO9C^∆CBM^, maybe due to a faster deactivation. The lack of activity of *Nc*LPMO9C^∆Seg2^ and *Nc*LPMO9C^∆Seg2, ∆CBM^ on XG indicates that Seg2 is important for the substrate recognition of XG.

The role of the CBM1 of *Nc*LPMO9C on substrate binding was already investigated by performing isothermal titration calorimetry (ITC) experiments with both RAC and XG [24]. The authors of the study demonstrated that the loss of the CBM1 resulted in a significantly higher *K*_d_ values for both substrates. The SPR experiments performed during this study were confirming this result. Nevertheless, we were not able to detect an interpretable signal in the absence of the CBM1. However, when turning to the *Nc*LPMOs 9F and 9M we were able to calculate dissociation constants despite the lack of a carbohydrate binding module. This is pointing towards an optimization of the catalytic site for binding without a CBM1 and might explain the difference in binding affinity between *Nc*LPMO9C and *Nc*LPMO9C^∆Seg2^. A recent study provided evidence that the reduction of the catalytic site copper is improving the binding affinity of *Nc*LPMO9C with a more than two times lower *K*_d_ (9.5 and 4.4 µM for the oxidized and the reduced LPMO, respectively) [46]. In preliminary SPR experiments with reduced enzymes we were able to confirm that the binding to a cellulosic substrate was greatly improved.

In conclusion, we could show that the shortening of Seg2 of *Nc*LPMO9C reduced thermal stability and the affinity to polysaccharide substrates. Moreover, our results confirm that the C-terminal linker between the *Nc*LPMO9C and its CBM1 plays a role in the thermal stability. Possibly, the linker is promoting the formation of a disulfide bridge which is stabilizing the protein fold. Furthermore, we could demonstrate that the CBM1 strongly contributes to substrate binding and does not influence the regioselectivity of the reaction.

## 4. Materials and Methods

### 4.1. Multiple Sequence Alignment, Phylogenetic Analysis and Homology Modelling

The multiple sequence alignment of fungal AA9 LPMO sequences was calculated with the structure-based MAFFT-DASH algorithm. The alignment and subsequent phylogenetic analysis were based on mature protein sequences selected from the CAZy database with a focus on fungi having multiple LPMOs. Phylogenetic analysis was performed with RaxML-NG [49]. ModelTest-NG [50] was used to find the best-fit substitution model. The evolutionary history was inferred by using the Maximum Likelihood method using the Wheelan & Goldman model [51] with frequencies, invariant sites, and the number of gamma distributed sites set to 4. Bootstraps analysis for tree inference was carried out until convergence (cut-off: 0.03; reached after 720 bootstraps) [52]. A second tree was established using only the putative surface substrate binding regions of the LPMOs using the same settings and the BLOSUM62 model with frequencies, invariant sites, and the number of gamma distributed sites set to 4. In both cases, the best-scoring most-likelihood tree is shown. The analyzed sequences grouped into three clusters and LPMOs originating from the same fungus were distributed in all three clusters. The phylogenetic trees of fungal LPMOs were linked to already-described substrate types. Homology model of *Tt*LPMO9B (GeneBank ID: AON76800.1) was generated using SWISS-MODEL [53,54,55,56,57] (template: PDB entry 5TKF, sequence identity: 41.86%, QMEAN: −2.03).

### 4.2. Chemicals and Substrates

All chemicals and the substrates carboxymethyl cellulose (CMC) and 2,6-dimethoxyphenol (2,6-DMP) were purchased form Sigma-Aldrich (St. Louis, MO, USA) unless indicated otherwise. Regenerated amorphous cellulose (RAC) was prepared as previously described by Zhang and coworkers [58]. Tamarind seed xyloglucan was purchased at Megazyme (Wicklow, Ireland).

### 4.3. Plasmid Design

The cDNA of *Nc*LPMO9M with the native signal peptide (NCU07898, XP_328604.1, EAA33178.1) and the modified *Nc*LPMO9C^∆Seg2^ with the *Nc*LPMO9C signal peptide were synthesized by BioCat GmbH (Heidelberg, Germany) and inserted into the pPICZ A vector by means of restriction enzymes BstBI and NotI. To generate the *Nc*LPMO9C^∆Seg2^ variant, nucleotides coding for residues 79–98 were replaced by the ones coding for residues 84–88 of *Nc*LPMO9F (amino acid numbering including signal peptide). To generate the expression plasmids coding for the *Nc*LPMOs 9C^∆CBM^ and 9C^∆Seg2, ∆CBM^, variants with the truncated linker and CBM1, prolines 228 and 213 respectively were mutated into an ochre stop codon using the PCR primers P228X_F and P228X_R (Supplementary Table S7). Successful mutations were confirmed by Sanger sequencing at Microsynth Austria GmbH (Vienna, Austria) using the inhouse sequencing primers 5-AOX1 and 3-AOX1.

### 4.4. Enzyme Production and Purification

*Nc*LPMO9C and *Nc*LPMO9F were produced by Kittl et al. [37]. *Nc*LPMO9M, *Nc*LPMO9C^∆seg2^, *Nc*LPMO9C^∆CBM^ and *Nc*LPMO9C^∆Seg2, ∆CBM^ were recombinantly expressed in *Pichia pastoris* X-33 as previously described [37]. The enzyme production was carried out in a 5 L fermenter [Eppendorf (Hamburg, Germany) BioFlo 120 system] with the addition of 5 µM CuSO_4_ and the enzyme was subsequently purified by column chromatography as published [37]. In brief, ammonium sulfate salt was added to the clear supernatant until a concentration of 30% was reached. Subsequently, the supernatant was loaded onto a 600 mL Phenyl-Sepharose Fast Flow column which was equilibrated with 50 mM sodium acetate, pH 5.0 containing 30% ammonium sulfate. The conductivity was matched to the supernatant by adding sodium chloride. A linear gradient from 30–0% ammonium sulfate over 3 column volumes was used to elute the proteins. Fractions containing LPMO activity were pooled and diafiltered with a Vivaflow crossflow module [MWCO 10000, Millipore (Burlington, MA, USA)]. A 60 mL Q15-Source column was equilibrated with 20 mM Tris/HCl buffer, pH 8.0 and the pooled fractions were loaded. As previously described, the LPMOs eluted in the flow-through. To reduce the volume the flow-through was diafiltrated with the same Vivaflow crossflow module as described above. *Nc*LPMO9C and *Nc*LPMO9C^∆CBM^ were further purified with a 500 mL Superdex 75 column equilibrated with 20 mM potassium phosphate buffer, pH 6.0. Finally, the activity of the purified LPMOs was confirmed by the recently published LPMO activity assay [36]. Supplementary Table S2 summarizes the purification steps and yields. The enzyme purity was verified by SDS-PAGE and peptide mapping as described below (Supplementary Figures S7 and S9 and Supplementary Table S8). The SDS-PAGE was prepared as follows. Lanes and molecular weights of the marker proteins (Precision Plus Protein Unstained Standards, Bio-Rad, Hercules, CA, USA) are indicated. The precast gel (4–20% Mini-PROTEAN^®^ TGX Stain-Free™ Precast Gel, Bio-Rad, Hercules, CA, USA) was stained with colloidal Coomassie blue [Bio-Safe™ Coomassie G.250 Stain, BioRad (Hercules, CA, USA)] for 1 h and destained overnight.

### 4.5. Protein Identification: Peptide Mapping Analysis

The sample was digested in solution. The protein mixture was reduced with dithiothreitol, S-alkylated with iodoacetamide and digested with Sequencing Grade Modified Trypsin [Promega (Madison, WI, USA)]. The digested samples were loaded on a BioBasic (Markham, ON, Canda) C18 column [BioBasic-18, 150 × 0.32 mm, 5 µm, Thermo Scientific (Waltham, MA, USA)] using 80 mM ammonium formate buffer as the aqueous solvent. A gradient from 96.5% solvent A and 3.5% solvent B (solvent A: 80 mM ammonium formate buffer at pH 3.0, B: 80% acetonitrile and 20% A) to 40% B in 45 min was applied, followed by a 15-min gradient from 40% B to 95% B, at a flow rate of 6 μL min^−1^. Detection was performed with a QTOF MS (Bruker maXis 4G) equipped with the standard ESI source in positive ion, DDA mode (= switching to MSMS mode for eluting peaks). MS-scans were recorded (range: 150–2200 m/z) and the 6 highest peaks were selected for fragmentation. Instrument calibration was performed using ESI calibration mixture (Agilent, Santa Clara, CA, USA). The analysis files were converted (using Data Analysis, Bruker, Billerica, MA, USA) to mgf files, which are suitable for performing a MS/MS ion search with Global Proteome Machine [GPM (Rockville, MD, USA); X! Tandem embedded, https://www.thegpm.org/tandem/]. The files were searched against a homemade database containing the target sequences.

### 4.6. LPMO Activity Assay

The activity and catalytic properties of the LPMOs were determined with the 2,6-DMP activity assay for LPMO [36]. The activity of the LPMOs was measured at pH 6.0 in a 100 mM sodium acetate buffer, pH 6.0 in the presence of 2 mM 2,6-DMP. To determine the Michaelis Menten kinetics, H_2_O_2_ was added to the reactions in the following final concentrations: 1.562, 3.125, 6.25, 12.5, 25, 50, 100 and 500 µM. The reactions were incubated in triplicates at 30 °C for 10 min before adding LPMO to the reactions. The absorbance at 469 nm was recorded in 1 mL cuvettes for 300 s using a PerkinElmer (Waltham, MA, USA) LAMBDA 35 Spectrophotometer.

### 4.7. Enzyme Incubations

Substrates (RAC, CMC, and XG) were suspended in 50 mM ammonium acetate buffer, pH 5.0 to a concentration of 2 g L^−1^. Subsequently, enzymes were added to a final concentration of 1.25 µM. In order to reduce the LPMO copper center, 1 mM ascorbic acid was added to the incubations. Control reactions were performed without the addition of ascorbic acid. All reactions were incubated at 30 °C in a head-over-tail rotator at 20 rpm (time curves; 5 mL total volume) or in an Eppendorf (Hamburg, Germany) Thermomixer comfort at 800 rpm (single 24 h reactions, 200 µL total volume). An incubation in an Eppendorf (Hamburg, Germany) ThermoMixer^®^ C at 97 °C for 10 min was performed to stop the enzyme reactions. Subsequently, the supernatant was recovered after centrifugation in a Hermle (HERMLE Labortechnik GmbH, Wehingen, Germany) Z 233 MK-2 centrifuge at 22,000× *g* (rotor: 220.87 VO5/6) and stored at −20 °C until further usage.

### 4.8. Differential Scanning Calorimetry

Differential scanning calorimetry (DSC) was performed on a MicroCal PEAQ-DSC Automated (Malvern Panalytical, Malvern, Worcestershire, UK) equipped with a 96-well plate autosampler holding at 4 °C. To prevent the solutions from boiling, the experiments were performed under increased pressure (~4.2 bar). At least four buffer runs (i.e., buffer in sample as well as reference cell) were performed at the beginning of an experiment to establish the thermal history of the cells. *Nc*LPMOs (15 µM in 50 mM potassium phosphate buffer, pH 6.0) were heated from 20 to 90 °C with a temperature ramp of 1 K min^−1^ in high feedback mode. Since the rescans (identical settings) showed some unfolding events, the baseline correction was performed with the buffer. The data analysis was performed using the MicroCal PEAQ-DSC Software version 1.4 (Malvern Panalytical, Malvern, Worcestershire, UK). Correction for sample baseline was done using the spline function. A non-two-state thermal unfolding model was fitted to the data points after subtraction of the buffer baselines and normalization for the protein concentration.

### 4.9. Electronic Circular Dichroism (ECD) Spectroscopy

A Chirascan circular dichroism spectrometer (Applied Photophysics, Leatherhead, Surrey, UK) was used to collect ECD spectra. The instrument was flushed with a nitrogen flow of 5 L min^−1^ throughout all experiments. The samples were analyzed at 30 °C using a 0.1 mm path length quartz cell. Spectra were recorded at a wavelength range of 190–280 nm with scan speed set to 5 s nm^−1^ at a bandwidth of 1 nm. Thermal denaturation experiments were performed from 40–90 °C with a constant heating rate of 1 °C min^−1^ at a wavelength range of 200–250 nm and a scan speed of 2 s nm^−1^ at a bandwidth of 1 nm. All measurements were performed in 50 mM phosphate buffer, pH 6.0 with a LPMO concentration of 3 mg mL^−1^. Data were analyzed with the PRO-DATA SX software version 2.2.17 (Applied Photophysics, Leatherhead, Surrey, UK). Transition midpoint temperatures were calculated from double sigmoidal non-linear curve fits at a wavelength of 229 nm, corresponding to the highest delta ellipticity of the thermal denaturation experiment. Secondary structure was predicted using the BeStSel web server (http://bestsel.elte.hu, accessed on 25 November 2019) from the far UV wavelength range of 200–250 nm and a scale factor of 1 [59].

### 4.10. HPAEC Analysis for Profiling Oligosaccharides

Degraded xyloglucan oligosaccharides were analyzed by high performance anion exchange chromatography (HPAEC) with pulsed amperometric detection (PAD) on a ICS5000 (Thermo Scientific, Waltham, MA, USA) system equipped with a CarboPac PA-1 column (2 mm ID × 250 mm) in combination with a CarboPac PA guard column (2 mm ID × 50 mm). The mobile phases used were (A) 0.1 M NaOH, (B) 1 M NaOAc in 0.1 M NaOH and the column temperature was 20 °C. The elution program applied has previously been described [41]. Samples were diluted 5-fold prior to analysis. Standard cellodextrins [DP 2–6; Sigma-Aldrich (St. Louis, MO, USA)] were mixed, each in a concentration of 2.5 µg mL^−1^ and used for calibration.

### 4.11. HPSEC Analysis for Molecular Weight Distribution of (Degraded) Xyloglucan

Xyloglucan and corresponding digests were analyzed by high performance size exclusion chromatography (HPSEC) for their molecular weight distribution. The analysis was performed on an Ultimate 3000 system (Thermo Scientific, Waltham, MA, USA) coupled to a Shodex RI-101 detector (Showa Denko K.K., Tokyo, Japan). Three TSK-Gel columns (SuperAW4000, SuperAW3000, SuperAW2500; 6 mm × 150 mm per column) in series combined with a TSK Gel super AW guard column (6 mm ID × 40 mm) (Tosoh Bioscience, Tokyo, Japan) were used with the column temperature of 55 °C. Supernatant (10 μL) of LPMO-xyloglucan digests was injected and eluted with 0.2 M NaNO_3_ at a flow rate of 0.6 mL min^−1^. Pullulans (Associated Polymer Labs Inc., New York, NY, USA) in the range of 0.4–708 kDa were used as calibrants.

### 4.12. Binding Studies on Cellulose

Microcrystalline cellulose (MCC) for column chromatography (Merck, Darmstadt, Germany) was dissolved in *N*,*N*-dimethylacetamide/LiCl to prepare cellulose films for SPR measurements. LiCl was dried at 200 °C for 24 h and afterwards stored in the desiccator over silica prior to use. Anhydrous *N*,*N*-dimethylacetamide (DMAc) was purchased from Sigma-Aldrich (St. Louis, MO, USA). Poly(diallyldimethylammoniumchloride) solution (PDADMAC) with an average molecular weight of 400,000 to 500,000 g mol^−1^ and a concentration of 20 wt.% dissolved in water was obtained from Sigma-Aldrich (St. Louis, MO, USA). Activation and dissolution of cellulose was performed by the procedure adapted from the procedure described by Raj et al. [60]. In brief, 2 g of MCC were swollen in 100 mL HQ-water constantly stirred at 22 °C for 18 h. The swollen cellulose fibers were further incubated in 25 mL methanol for 45 min at 40 °C twice before they were swollen in 25 mL DMAc at 22 °C for 45 min for four times. Between the washing and swelling steps, the fibers were recovered using a paper filter. The same filter was used throughout the different steps to minimize losses. Activated cellulose was dried under vacuum at 60 °C for 24 h and stored over silica prior to further use. Water free LiCl was dissolved to a final concentration of 7 wt.% in anhydrous DMAc at 40 °C. This solvent system was used to dissolve activated cellulose by stirring at 22 °C for 18 h to obtain a clear cellulose solution. 

SPR sensors were prepared using the SIA Kit Au (GE Healthcare, Chicago, IL, USA). The method for the preparation of cellulose films on gold surfaces was modified from the procedure described by Sczech and Riegler [61]. Gold-coated targets were cleaned using alkaline Piranha solution containing NH_4_OH/H_2_O_2_/H_2_O in a 1:1:3 ratio at 75 °C for 15 min and rinsed with HQ-water and dried at 80 °C. PDADMAC was used as adhesion promoter. The cleaned targets were covered with a solution of 100 mg L^−1^ PDADMAC and 0.01 M NaCl for 30 min and rinsed with HQ-water. Afterwards, the targets were dried at 80 °C and stored over silica prior to their modification with cellulose. The cellulose film was deposited on the gold surface by spin-coating. For that, 80 µL of 0.5 wt.% cellulose in DMAc/LiCl were deposited at 3000 rpm for 3 min and the solvent was removed by drying at 160 °C. The cellulose film was extensively washed in HQ-water to remove LiCl and the target was dried at 160 °C. Finally, the sensor chip was assembled according the instructions of the manufacturer. 

Surface plasmon resonance spectroscopy (SPR) was performed with a Biacore T200 system (GE Healthcare, Chicago, IL, USA). All experiments were performed at 30 °C at a flow rate of 10 µL min^−1^ in Dulbecco’s phosphate buffered saline supplemented with 0.05 wt.% Tween 20. Binding kinetics were determined using single-cycle-kinetic measurements with five sequential injections of the analyte (LPMO) with an association time of 60 s and a final dissociation of 180 s without regeneration between the injections of one cycle. Between the cycles, the surface was regenerated using 4 M MgCl_2_ for 60 s and 10 mM glycine/HCl (pH 2.5) for 30 s at the same flow rate. For the initial equilibration of the surface three cycles of blank injections with running buffer and the same regeneration procedure were applied. Each measurement was performed in three independent runs on independent flow channels. Data were evaluated using the BiaEvaluation Software version 3.1 (GE Healthcare, Chicago, IL, USA) and SigmaPlot version 12.0 (Systat Software Inc., San Jose, CA, USA). To study the binding behavior of LPMO in its reduced state to the cellulosic substrate the running buffer, solutions used for regeneration of the surface and sample solutions were degassed applying vacuum, purged with nitrogen and finally with argon immediately before the measurement to remove oxygen. The running buffer and the sample solutions were supplemented with 10 mM gallic acid to reduce the active site copper.

## Figures and Tables

**Table 1:** Kinetic constants with standard error of the mean of *Nc*LPMOs and variants for H_2_O_2_ using 2 mM 2,6-DMP as substrate and reductant in 100 mM sodium acetate buffer, pH 6.0 (*n* = 3).

Enzyme	*K*_M_ [µM]	Spec. act. with 100 µM H_2_O_2_ [U g^−1^]	*k_cat_* [s^−1^]	*k_cat_/K*_M_ [µM^−1^ s^−1^]
*Nc*LPMO9C	2.8 ± 0.1	28.8 ± 0.2	0.57 ± 0.03	0.20 ± 0.02
*Nc*LPMO9C^∆CBM^	1.4 ± 0.1	13.0 ± 0.1	0.39 ± 0.01	0.28 ± 0.03
*Nc*LPMO9C^∆Seg2^	5.4 ± 0.1	42.4 ± 0.2	0.55 ± 0.01	0.10 ± 0.01
*Nc*LPMO9C^∆Seg2, ∆CBM^	4.0 ± 0.1	41.1 ± 0.3	0.37 ± 0.01	0.09 ± 0.01
*Nc*LPMO9M	7.0 ± 0.3	39.1 ± 0.3	0.40 ± 0.01	0.06 ± 0.01

## Supplementary Materials

Supplementary materials can be found at https://www.mdpi.com/1422-0067/20/24/6219/s1.

## Abbreviations

AA	Auxiliary activity enzyme
CAYZy	Carbohydrate-active enzyme
CBM1	Carbohydrate binding module family 1
CMC	Carboxymethyl cellulose
DP	Degree of polymerization
DSC	Differential scanning calorimetry
ECD	Electronic circular dichroism
HPAEC-PAD	High performance anion exchange chromatography coupled to a pulsed amperometric detector
HPSEC-RI	High performance size exclusion chromatography coupled to a refractive index detector
LC-ESI-MS	Liquid chromatography-electrospray ionization mass spectrometry
LPMO	Lytic polysaccharide monooxygenase
RAC	Regenerated amorphous cellulose
SPR	Surface plasmon resonance
XG	Tamarind seed xyloglucan
