# Supplementary material for: Influence of Lytic Polysaccharide Monooxygenase Active Site Segments on Activity and Affinity
Source: Int J Mol Sci. 2019 Dec 10;20(24):6219. doi: 10.3390/ijms20246219 (PMC6940765; doi:10.3390/ijms20246219)
Supplement: Supplementary file 1 [file ijms-20-06219-s001.pdf]

Influence of Lytic Polysaccharide Monooxygenase Active Site Segments on Activity and Affinity

Christophe V.F.P. Laurent[1,2*], Peicheng Sun[3*], Stefan Scheiblbrandner[1], Florian Csarman[1], Pietro Cannazza[1,3], Matthias Frommhagen[4], Willem J.H. van Berkel[4], Chris Oostenbrink[2], Mirjam A. Kabel[4] and Roland Ludwig[1†]

[1] Biocatalysis and Biosensing Laboratory, Department of Food Science and Technology, BOKU – University of Natural Resources and Life Sciences, Vienna, Muthgasse 18, 1190 Vienna, Austria
[2] Institute of Molecular Modeling and Simulation, Department of Material Sciences and Process Engineering BOKU – University of Natural Resources and Life Sciences, Vienna, Muthgasse 18, 1190 Vienna, Austria
[3] Department of Food, Environmental and Nutritional Sciences (DeFENS), Università degli Studi di Milano, Via Mangiagalli 25, 20133 Milano, Italy
[4] Laboratory of Food Chemistry, Wageningen University & Research, Bornse Weilanden 9, 6708 WG Wageningen, The Netherlands
[†] Correspondence: roland.ludwig@boku.ac.at; Tel.: +43 1 47654-75216
*These authors contributed equally to this work

Supplementary Material

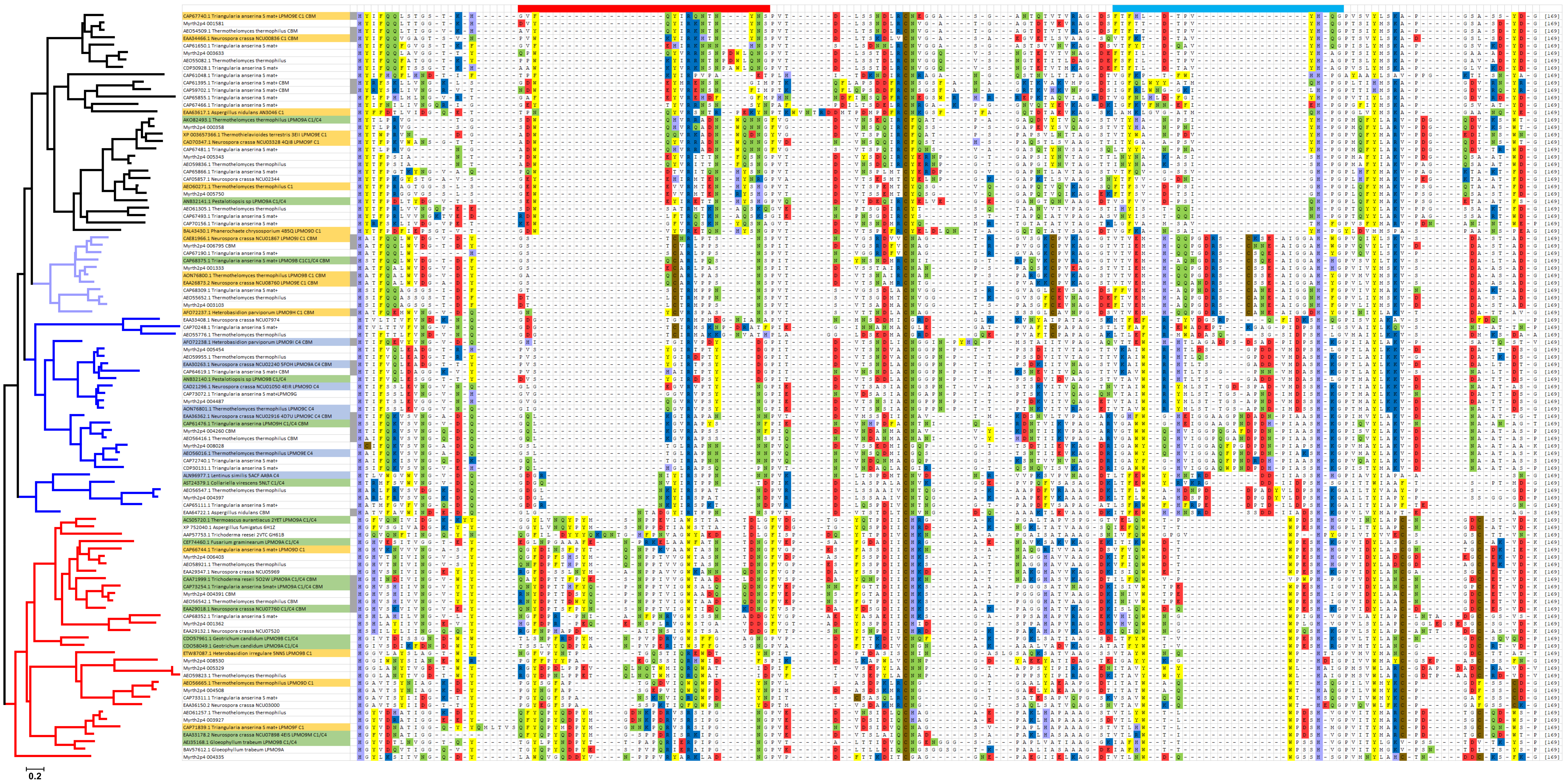
0.2

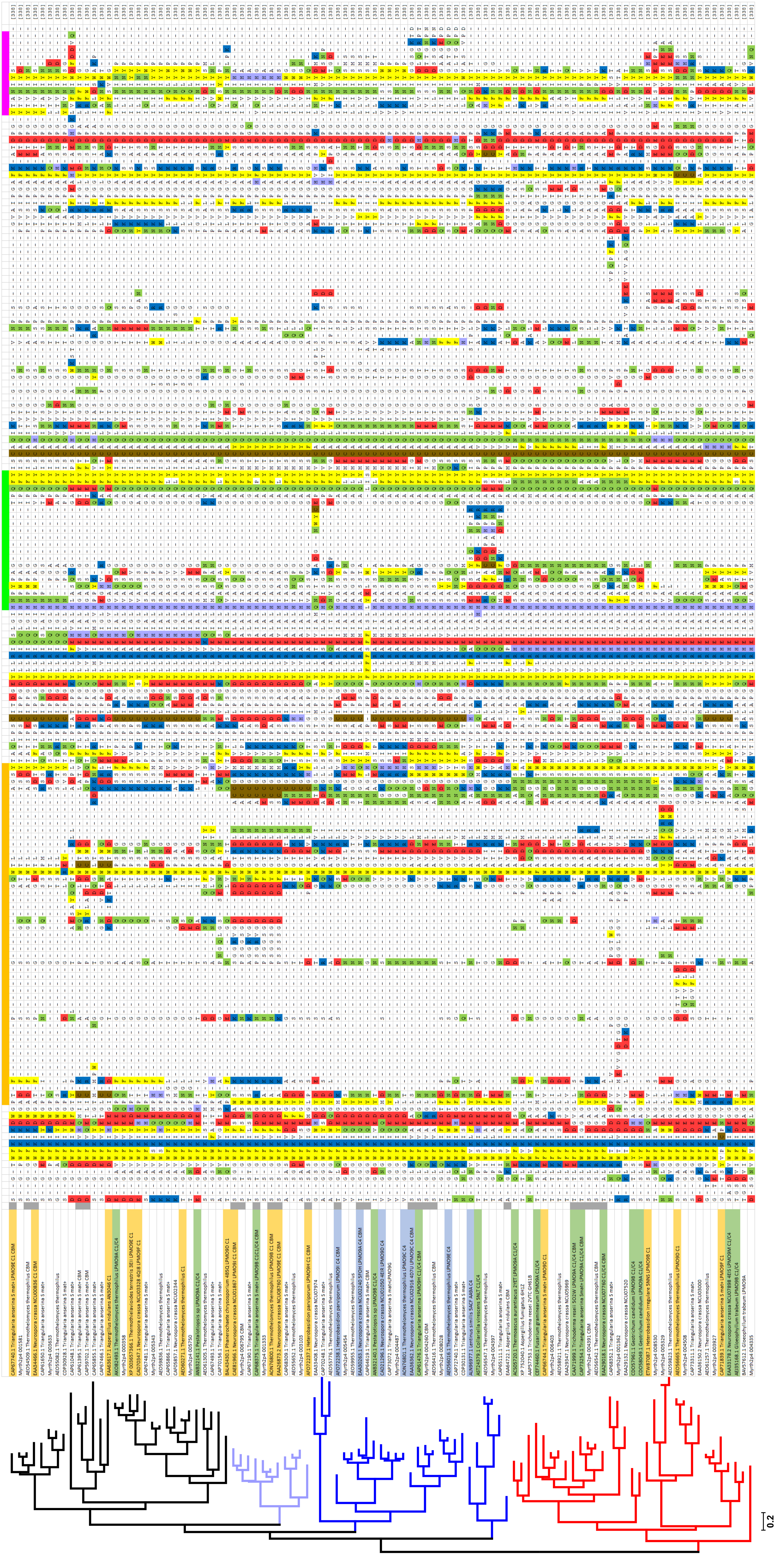

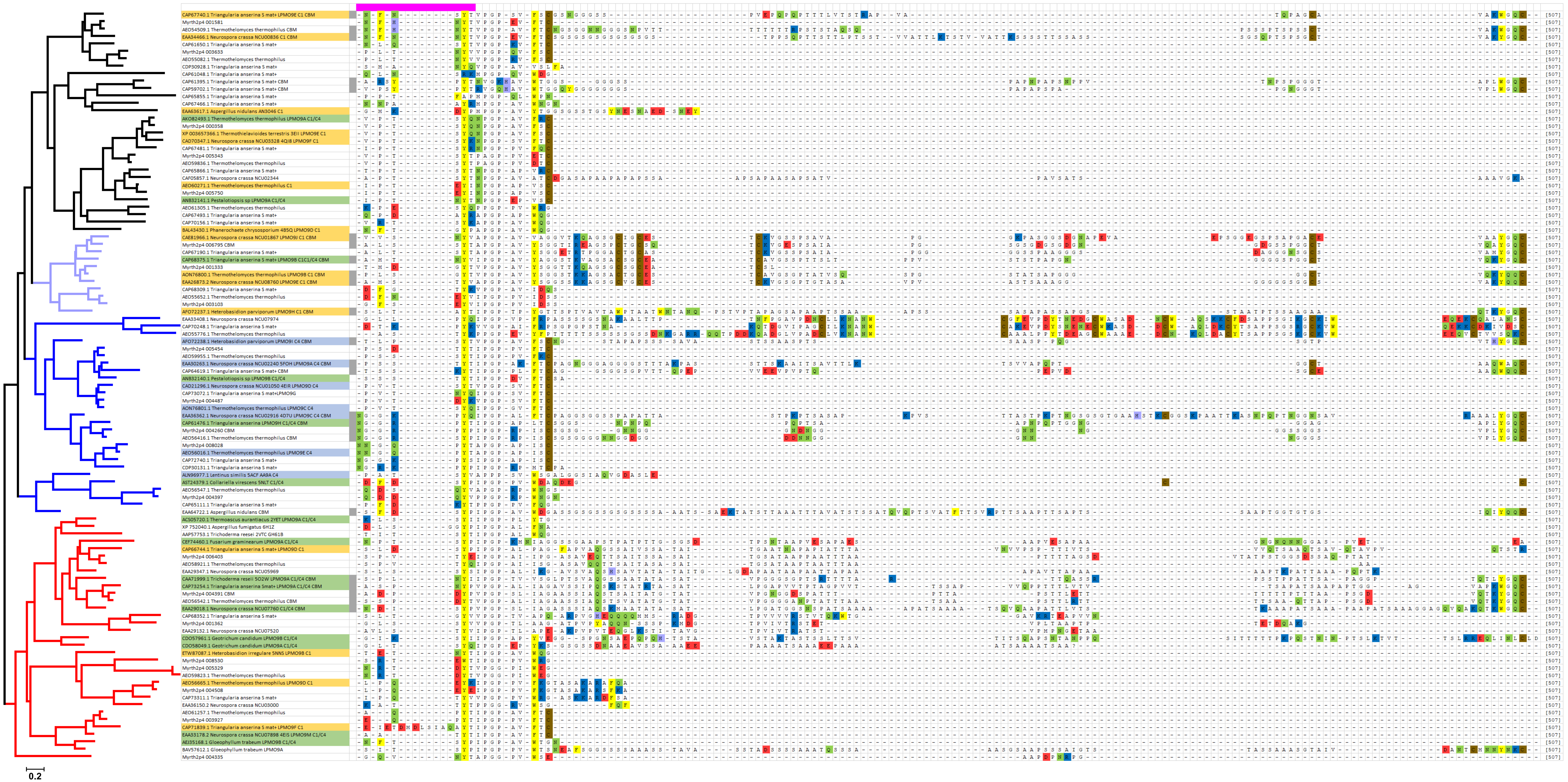

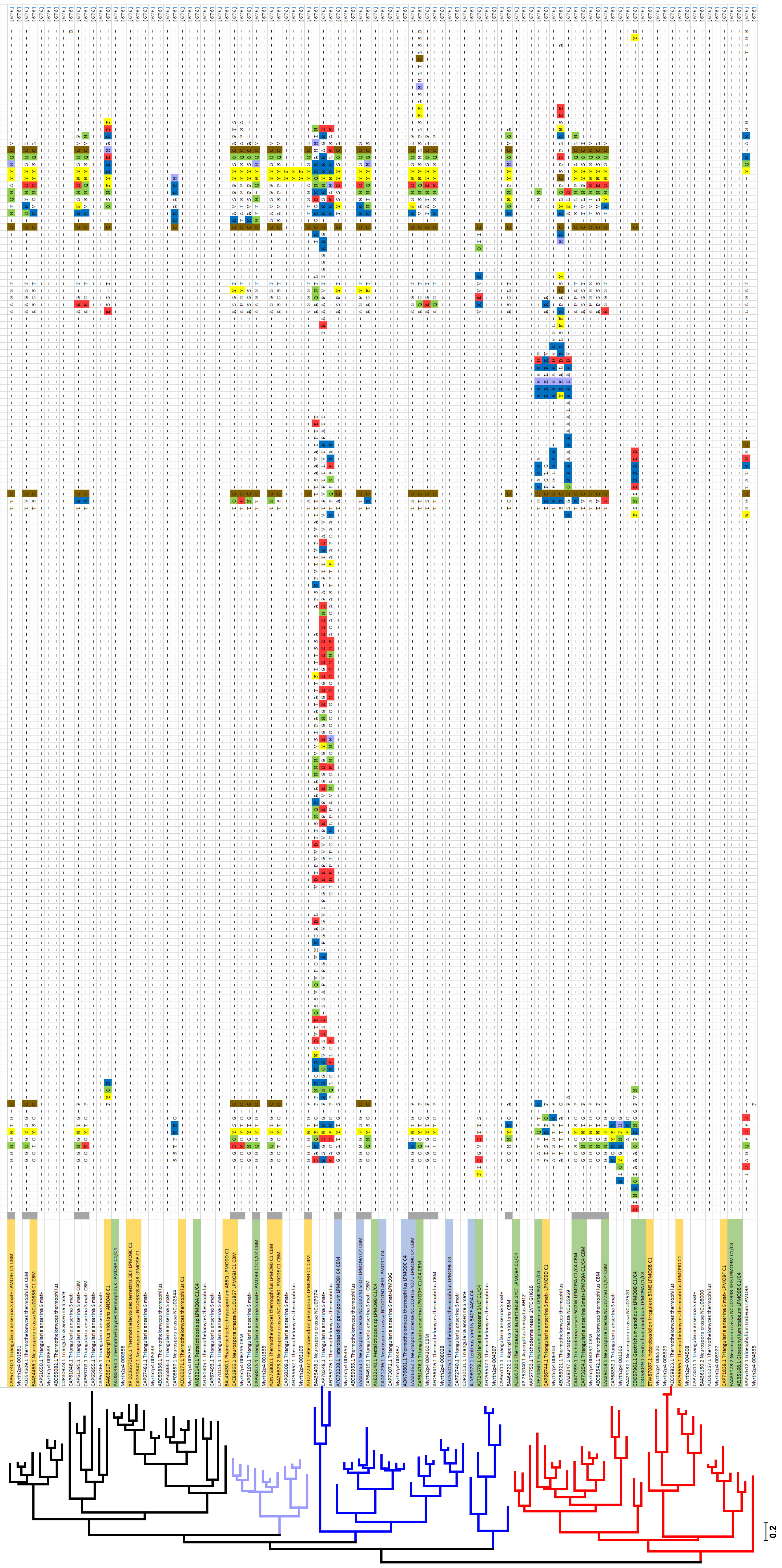
0.2

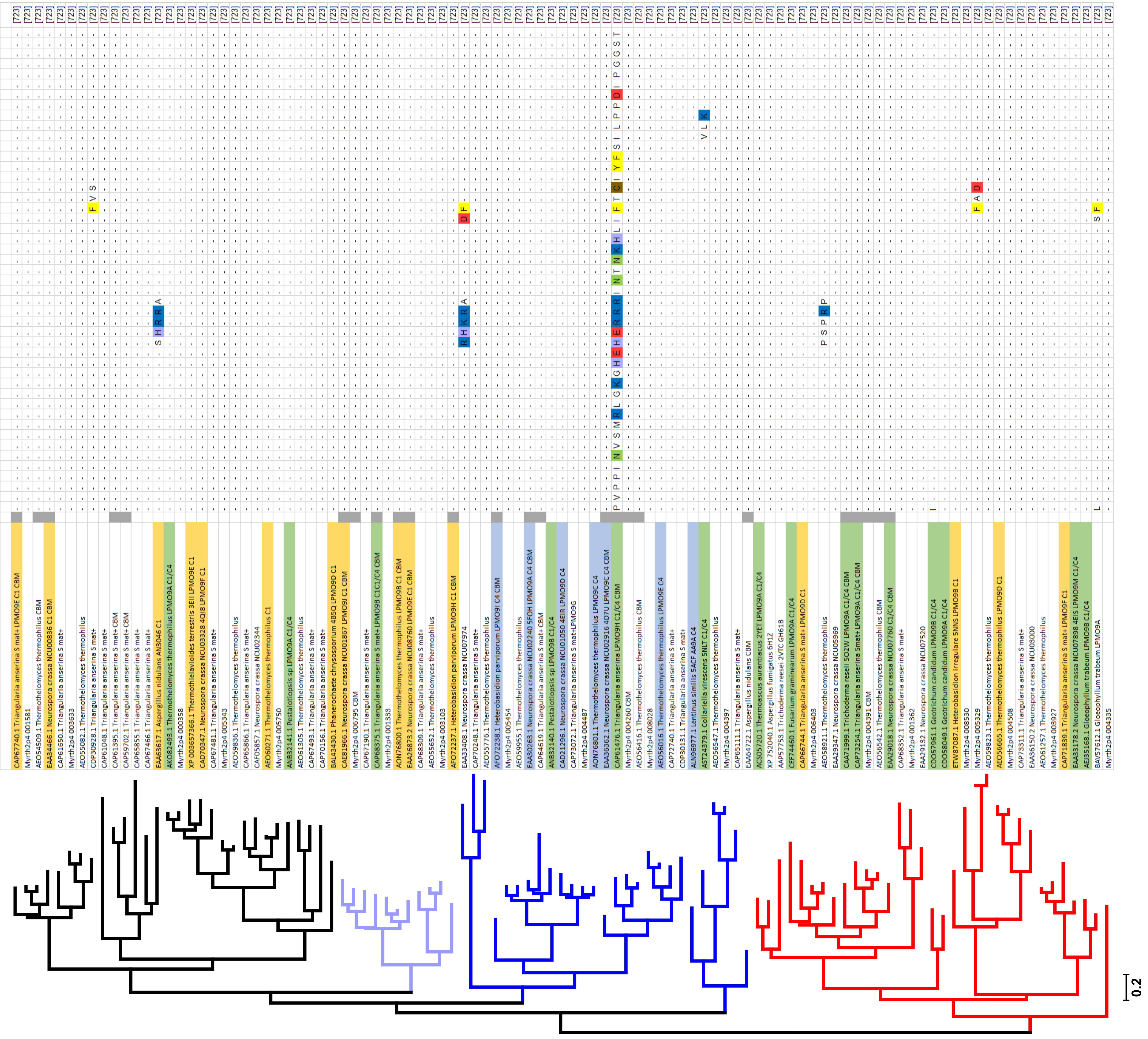

Figure S1. Full length multiple sequence alignment without linker region and CBM and associated phylogenetic tree of AA9 LPMOs. All available AA9 LPMO sequences of *Neurospora crassa, Podospora anserina* S mat+ (syn. *Triangularia anserina* S mat+), *Thermothelomyces thermophilum* (syn. *Myceliophthora thermophila*), *Crassicarpon hotsonii* (syn *Myriococcum thermophilum*) as well as all AA9 LPMOs listed under the headers "characterized" and "structure" in the CAZy database were aligned using the MAFFT-DASH algorithm based on the protein secondary structure. Prior to phylogenetic analysis the C-terminal linker region and the CBM1 were deleted from the alignment. Phylogeny was inferred by using the RaxML-NG algorithm applying the Wheelan & Goldman model with frequencies and gamma distribution (4). The color code of the tree corresponds to the top line of the alignment and indicates the respective segments: Seg1, red; Seg2, blue; Seg3, orange; Seg4, green; Seg5, magenta. Gray squares next to the sequence name column indicate the presence of a CBM1. The color code used to color sequence names indicates the regioselectivity of AA9 LPMOs as previously published [1]: C1, yellow; C4, blue; C1/C4, green. Amino acids considered relevant for catalysis or substrate interaction are colored in the multiple sequence alignment: His, light blue; Arg and Lys, blue; Asp and Glu, red; Asn and Gln, green; Tyr and Phe, yellow.

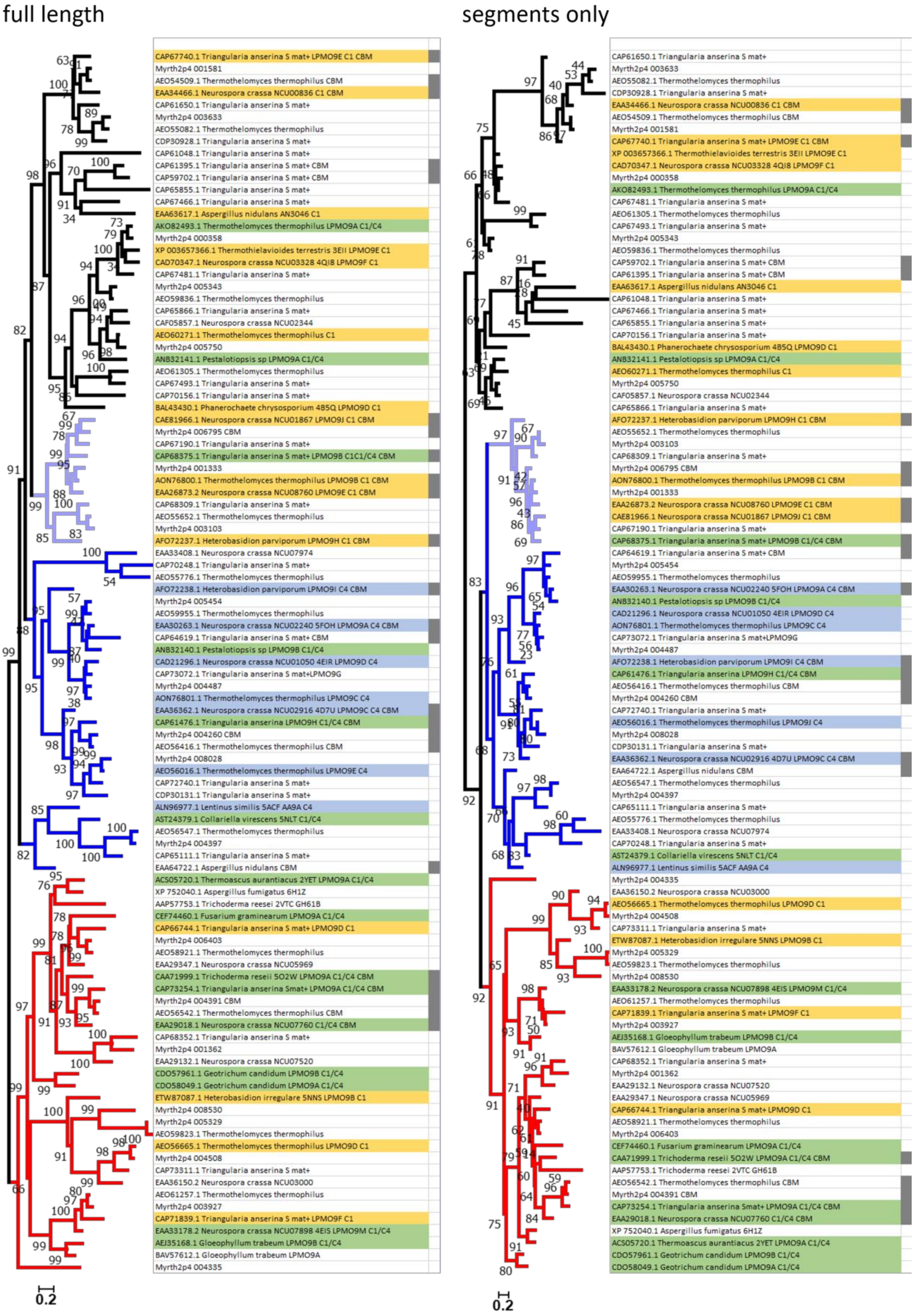
full length
CAP67740.1 Triangularia anserina S mat+ LPMO9E C1 CBM
Myrth2p4 001581
AEO54509.1 Thermothelomyces thermophilus CBM
EAA34466.1 Neurospora crassa NCU00836 C1 CBM
CAP61650.1 Triangularia anserina S mat+
Myrth2p4 003633
AEO55082.1 Thermothelomyces thermophilus
CDP30928.1 Triangularia anserina S mat+
CAP61048.1 Triangularia anserina S mat+
CAP61395.1 Triangularia anserina S mat+ CBM
CAP59702.1 Triangularia anserina S mat+ CBM
CAP65855.1 Triangularia anserina S mat+
CAP67466.1 Triangularia anserina S mat+
EAA63617.1 Aspergillus nidulans AN3046 C1
AKO82493.1 Thermothelomyces thermophilus LPMO9A C1/C4
Myrth2p4 000358
XP 003657366.1 Thermothielavioides terrestris 3EII LPMO9E C1
CAD70347.1 Neurospora crassa NCU03328 4QI8 LPMO9F C1
CAP67481.1 Triangularia anserina S mat+
Myrth2p4 005343
AEO59836.1 Thermothelomyces thermophilus
CAP65866.1 Triangularia anserina S mat+
CAF05857.1 Neurospora crassa NCU02344
AEO60271.1 Thermothelomyces thermophilus C1
Myrth2p4 005750
ANB32141.1 Pestalotiopsis sp LPMO9A C1/C4
AEO61305.1 Thermothelomyces thermophilus
CAP67493.1 Triangularia anserina S mat+
CAP70156.1 Triangularia anserina S mat+
BAL43430.1 Phanerochaete chrysosporium 4B5Q LPMO9D C1
CAE81966.1 Neurospora crassa NCU01867 LPMO9J C1 CBM
Myrth2p4 006795 CBM
CAP67190.1 Triangularia anserina S mat+
CAP68375.1 Triangularia anserina S mat+ LPMO9B C1C1/C4 CBM
Myrth2p4 001333
AON76800.1 Thermothelomyces thermophilus LPMO9B C1 CBM
EAA26873.2 Neurospora crassa NCU08760 LPMO9E C1 CBM
CAP68309.1 Triangularia anserina S mat+
AEO55652.1 Thermothelomyces thermophilus
Myrth2p4 003103
AFO72237.1 Heterobasidion parviporum LPMO9H C1 CBM
EAA33408.1 Neurospora crassa NCU07974
CAP70248.1 Triangularia anserina S mat+
AEO55776.1 Thermothelomyces thermophilus
AFO72238.1 Heterobasidion parviporum LPMO9I C4 CBM
Myrth2p4 005454
AEO59955.1 Thermothelomyces thermophilus
EAA30263.1 Neurospora crassa NCU02240 5FOH LPMO9A C4 CBM
CAP64619.1 Triangularia anserina S mat+ CBM
ANB32140.1 Pestalotiopsis sp LPMO9B C1/C4
CAD21296.1 Neurospora crassa NCU01050 4EIR LPMO9D C4
CAP73072.1 Triangularia anserina S mat+LPMO9G
Myrth2p4 004487
AON76801.1 Thermothelomyces thermophilus LPMO9C C4
EAA36362.1 Neurospora crassa NCU02916 4D7U LPMO9C C4 CBM
CAP61476.1 Triangularia anserina LPMO9H C1/C4 CBM
Myrth2p4 004260 CBM
AEO56416.1 Thermothelomyces thermophilus CBM
Myrth2p4 008028
AEO56016.1 Thermothelomyces thermophilus LPMO9E C4
CAP72740.1 Triangularia anserina S mat+
CDP30131.1 Triangularia anserina S mat+
ALN96977.1 Lentinus similis 5ACF AA9A C4
AST24379.1 Collariella virescens 5NLT C1/C4
AEO56547.1 Thermothelomyces thermophilus
Myrth2p4 004397
CAP65111.1 Triangularia anserina S mat+
EAA64722.1 Aspergillus nidulans CBM
ACS05720.1 Thermoascus aurantiacus 2YET LPMO9A C1/C4
XP 752040.1 Aspergillus fumigatus 6H1Z
AAP57753.1 Trichoderma reesei 2VTC GH61B
CEF74460.1 Fusarium graminearum LPMO9A C1/C4
CAP66744.1 Triangularia anserina S mat+ LPMO9D C1
Myrth2p4 006403
AEO58921.1 Thermothelomyces thermophilus
EAA29347.1 Neurospora crassa NCU05969
CAA71999.1 Trichoderma reseii 5O2W LPMO9A C1/C4 CBM
CAP73254.1 Triangularia anserina Smat+ LPMO9A C1/C4 CBM
Myrth2p4 004391 CBM
AEO56542.1 Thermothelomyces thermophilus CBM
EAA29018.1 Neurospora crassa NCU07760 C1/C4 CBM
CAP68352.1 Triangularia anserina S mat+
Myrth2p4 001362
EAA29132.1 Neurospora crassa NCU07520
CDO57961.1 Geotrichum candidum LPMO9B C1/C4
CDO58049.1 Geotrichum candidum LPMO9A C1/C4
ETW87087.1 Heterobasidion irregulare 5NNS LPMO9B C1
Myrth2p4 008530
Myrth2p4 005329
AEO59823.1 Thermothelomyces thermophilus
AEO56665.1 Thermothelomyces thermophilus LPMO9D C1
Myrth2p4 004508
CAP73311.1 Triangularia anserina S mat+
EAA36150.2 Neurospora crassa NCU03000
AEO61257.1 Thermothelomyces thermophilus
Myrth2p4 003927
CAP71839.1 Triangularia anserina S mat+ LPMO9F C1
EAA33178.2 Neurospora crassa NCU07898 4EIS LPMO9M C1/C4
AEJ35168.1 Gloeophyllum trabeum LPMO9B C1/C4
BAV57612.1 Gloeophyllum trabeum LPMO9A
Myrth2p4 004335
0.2
segments only
CAP61650.1 Triangularia anserina S mat+
Myrth2p4 003633
AEO55082.1 Thermothelomyces thermophilus
CDP30928.1 Triangularia anserina S mat+
EAA34466.1 Neurospora crassa NCU00836 C1 CBM
AEO54509.1 Thermothelomyces thermophilus CBM
Myrth2p4 001581
CAP67740.1 Triangularia anserina S mat+ LPMO9E C1 CBM
XP 003657366.1 Thermothielavioides terrestris 3EII LPMO9E C1
CAD70347.1 Neurospora crassa NCU03328 4QI8 LPMO9F C1
Myrth2p4 000358
AKO82493.1 Thermothelomyces thermophilus LPMO9A C1/C4
CAP67481.1 Triangularia anserina S mat+
AEO61305.1 Thermothelomyces thermophilus
CAP67493.1 Triangularia anserina S mat+
Myrth2p4 005343
AEO59836.1 Thermothelomyces thermophilus
CAP59702.1 Triangularia anserina S mat+ CBM
CAP61395.1 Triangularia anserina S mat+ CBM
EAA63617.1 Aspergillus nidulans AN3046 C1
CAP61048.1 Triangularia anserina S mat+
CAP67466.1 Triangularia anserina S mat+
CAP65855.1 Triangularia anserina S mat+
CAP70156.1 Triangularia anserina S mat+
BAL43430.1 Phanerochaete chrysosporium 4B5Q LPMO9D C1
ANB32141.1 Pestalotiopsis sp LPMO9A C1/C4
AEO60271.1 Thermothelomyces thermophilus C1
Myrth2p4 005750
CAF05857.1 Neurospora crassa NCU02344
CAP65866.1 Triangularia anserina S mat+
AFO72237.1 Heterobasidion parviporum LPMO9H C1 CBM
AEO55652.1 Thermothelomyces thermophilus
Myrth2p4 003103
CAP68309.1 Triangularia anserina S mat+
Myrth2p4 006795 CBM
AON76800.1 Thermothelomyces thermophilus LPMO9B C1 CBM
Myrth2p4 001333
EAA26873.2 Neurospora crassa NCU08760 LPMO9E C1 CBM
CAE81966.1 Neurospora crassa NCU01867 LPMO9J C1 CBM
CAP67190.1 Triangularia anserina S mat+
CAP68375.1 Triangularia anserina S mat+ LPMO9B C1/C4 CBM
CAP64619.1 Triangularia anserina S mat+ CBM
Myrth2p4 005454
AEO59955.1 Thermothelomyces thermophilus
EAA30263.1 Neurospora crassa NCU02240 5FOH LPMO9A C4 CBM
ANB32140.1 Pestalotiopsis sp LPMO9B C1/C4
CAD21296.1 Neurospora crassa NCU01050 4EIR LPMO9D C4
AON76801.1 Thermothelomyces thermophilus LPMO9C C4
CAP73072.1 Triangularia anserina S mat+LPMO9G
Myrth2p4 004487
AFO72238.1 Heterobasidion parviporum LPMO9I C4 CBM
CAP61476.1 Triangularia anserina LPMO9H C1/C4 CBM
AEO56416.1 Thermothelomyces thermophilus CBM
Myrth2p4 004260 CBM
CAP72740.1 Triangularia anserina S mat+
AEO56016.1 Thermothelomyces thermophilus LPMO9J C4
Myrth2p4 008028
CDP30131.1 Triangularia anserina S mat+
EAA36362.1 Neurospora crassa NCU02916 4D7U LPMO9C C4 CBM
EAA64722.1 Aspergillus nidulans CBM
AEO56547.1 Thermothelomyces thermophilus
Myrth2p4 004397
CAP65111.1 Triangularia anserina S mat+
AEO55776.1 Thermothelomyces thermophilus
EAA33408.1 Neurospora crassa NCU07974
CAP70248.1 Triangularia anserina S mat+
AST24379.1 Collariella virescens 5NLT C1/C4
ALN96977.1 Lentinus similis 5ACF AA9A C4
Myrth2p4 004335
EAA36150.2 Neurospora crassa NCU03000
AEO56665.1 Thermothelomyces thermophilus LPMO9D C1
Myrth2p4 004508
CAP73311.1 Triangularia anserina S mat+
ETW87087.1 Heterobasidion irregulare 5NNS LPMO9B C1
Myrth2p4 005329
AEO59823.1 Thermothelomyces thermophilus
Myrth2p4 008530
EAA33178.2 Neurospora crassa NCU07898 4EIS LPMO9M C1/C4
AEO61257.1 Thermothelomyces thermophilus
CAP71839.1 Triangularia anserina S mat+ LPMO9F C1
Myrth2p4 003927
AEJ35168.1 Gloeophyllum trabeum LPMO9B C1/C4
BAV57612.1 Gloeophyllum trabeum LPMO9A
CAP68352.1 Triangularia anserina S mat+
Myrth2p4 001362
EAA29132.1 Neurospora crassa NCU07520
EAA29347.1 Neurospora crassa NCU05969
CAP66744.1 Triangularia anserina S mat+ LPMO9D C1
AEO58921.1 Thermothelomyces thermophilus
Myrth2p4 006403
CEF74460.1 Fusarium graminearum LPMO9A C1/C4
CAA71999.1 Trichoderma reseii 5O2W LPMO9A C1/C4 CBM
AAP57753.1 Trichoderma reesei 2VTC GH61B
AEO56542.1 Thermothelomyces thermophilus CBM
Myrth2p4 004391 CBM
CAP73254.1 Triangularia anserina Smat+ LPMO9A C1/C4 CBM
EAA29018.1 Neurospora crassa NCU07760 C1/C4 CBM
XP 752040.1 Aspergillus fumigatus 6H1Z
ACS05720.1 Thermoascus aurantiacus 2YET LPMO9A C1/C4
CDO57961.1 Geotrichum candidum LPMO9B C1/C4
CDO58049.1 Geotrichum candidum LPMO9A C1/C4
0.2

Figure S2. Comparison of the phylogenetic trees inferred from full length (left) and "segments only" (right) multiple sequence alignments. The three main clusters are color coded (Cluster 1, black; Cluster 2, blue; Cluster 3) and a subgroup of sequences changing from Cluster 1 to Cluster 2 when phylogenetic inference is not based on the full dataset, but the sequence information is highlighted in pale blue. Taxa color code: yellow, C1-oxidizing; blue, C4-oxidizing; green, C1/C4-oxidizing. Gray boxes besides the taxa names indicate the presence of a CBM.

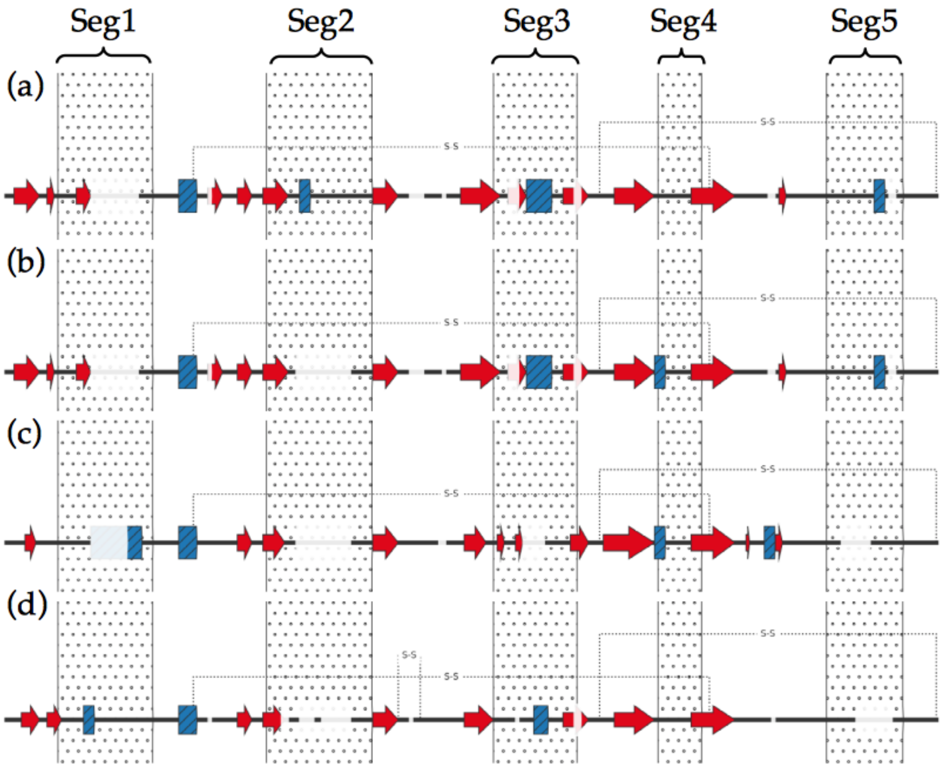

Figure S3. Schematic representation of the secondary structure elements of the catalytic domains. (a) *Nc*LPMO9C, (b) *Nc*LPMO9C$^{\Delta Seg2}$, (c) *Nc*LPMO9F and (d) *Nc*LPMO9M. α-Helices and ß-strands are shown in blue and red, respectively. Disulfide bonds are depicted as dotted lines. Transparent regions are indicating gaps in the multiple sequence alignment and dotted regions are highlighting the Seg1–5 are defined as given in Supplmentary Table S1. the secondary structure elements were determined using the DSSP algorithm [2] as implemented in GROMOS++ [3]

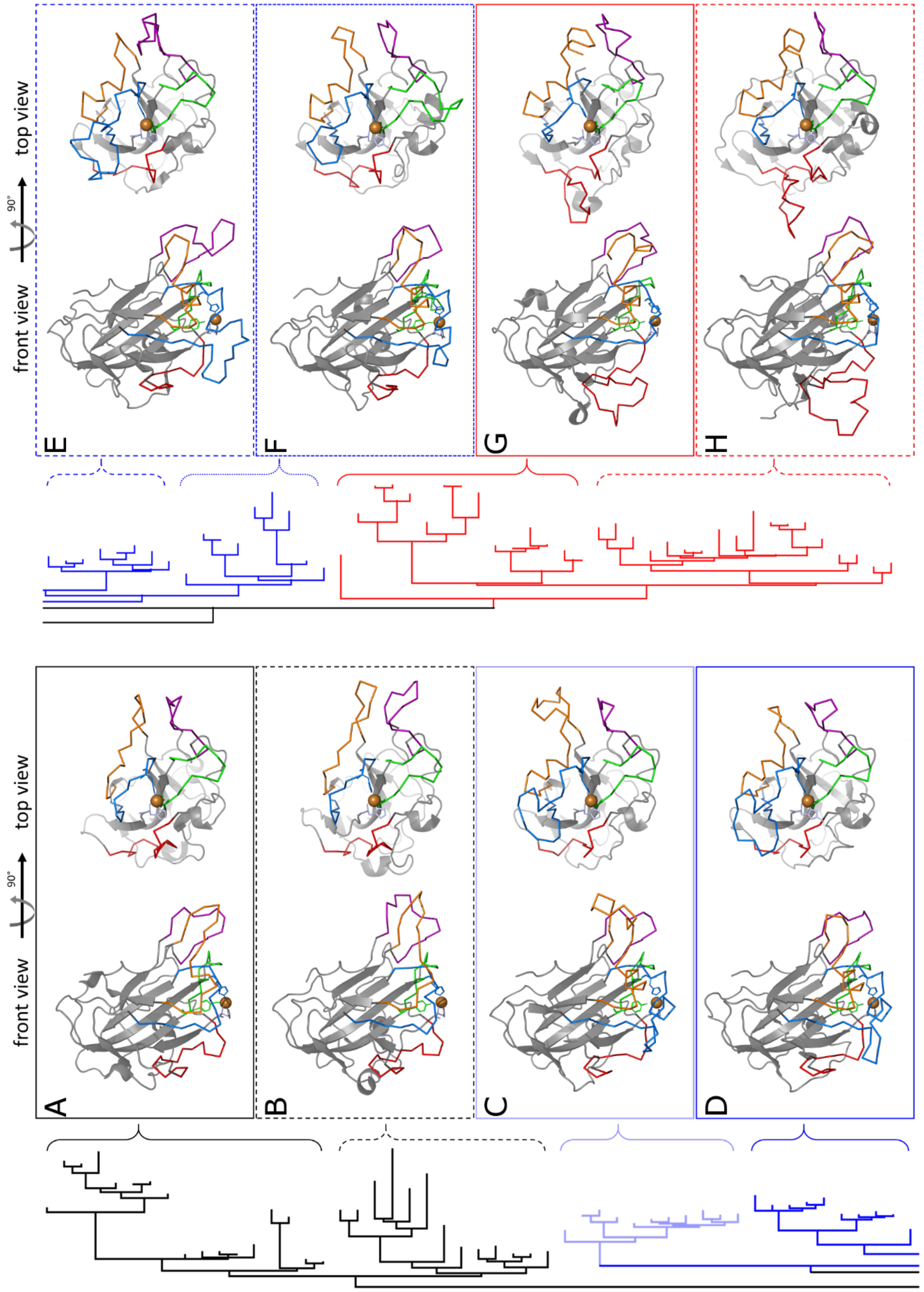

Figure S4. Front and top view of crystal structures of selected AA9 LPMOs. The crystal structures and homology models of eight LPMOs is shown in "front view", at which the copper atom and the putative, planar-like substrate surface binding region is facing the bottom, and "top view), at which the previous visualization is rotated by

90°. Each structure represents one of eight distinct clades of the phylogenetic tree. (A) *Nc*LPMO9F (GeneBank: CAD70347.1; PDB: 4QI8), (B) *Pc*LPMO9D (GeneBank: BAL43430.1; PDB: 4B5Q), (C) *Tt*LPMO9B (GeneBank: AON76800.1; SwissModel template: 5TKF with 41.68 % sequence identity), (D) *Nc*LPMO9D (GeneBank: CAD21296.1; PDB: 4EIR), (E) *Nc*LPMO9C (GeneBank: EAA36362.1; PDB 4D7U), (F) *Cv*AA9A (GeneBank: AST24379.1; PDB: 5NLT), (G) *Hi*LPMO9B (GeneBank: ETW87087.1; PDB: 5NNS), and (H) *Tr*LPMO9A (GeneBank: CAA71999.1; PDB: 5O2W). Segments 1 to 5 are colored according to the previously introduced color code (S1: red; S2: blue; S3: orange; S4: green; S5: purple). The catalytic copper atom is shown as orange sphere.

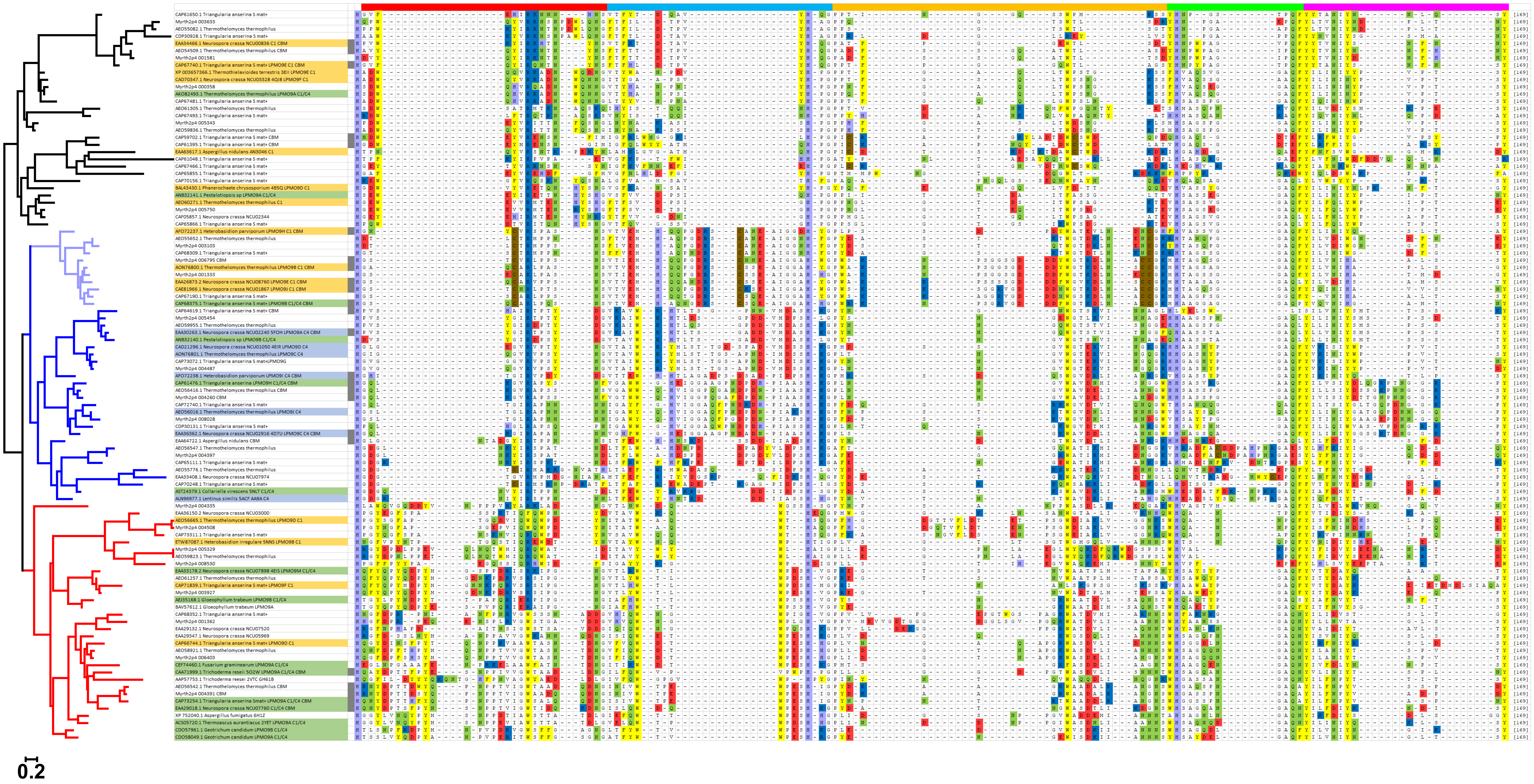

Figure S5. “Segments-only” multiple sequence alignment. All available AA9 LPMO sequences of *Neurospora crassa*, *Podospora anserina* S mat+ (syn. *Triangularia anserina* S mat+), *Thermothelomyces thermophilum* (syn. *Myceliophthora thermophila*), *Crassicarpon hotsonii* (syn *Myriococcum thermophilum*) as well as all AA9 LPMOs listed under the headers “characterized” and “structure” in the CAZy database were aligned using the MAFFT-DASH algorithm based on the protein secondary structure. The multiple sequence alignment was then assembled in a way that left only the indicated segments (Seg1–5) in the alignment. The phylogeny was inferred by the RaxML-NG algorithm applying the Wheelan & Goldman model with frequencies and gamma distribution (4). The color code of the tree corresponds to the top line of the alignment and indicates the position/possession of the respective segments: Seg1, red; Seg2, blue; Seg3, orange; Seg4, green; Seg5, magenta. Gray squares next to the sequence name column indicate the presence of a CBM1. The color code used to color sequence names indicates the regioselectivity of AA9 LPMOs as previously published [1]: C1, yellow; C4, blue; C1/C4, green. Amino acids considered relevant for catalysis or substrate interaction are colored in the multiple sequence alignment: His, light blue; Arg and Lys, blue; Asp and Glu, red; Asn and Gln, green; Tyr and Phe, yellow.

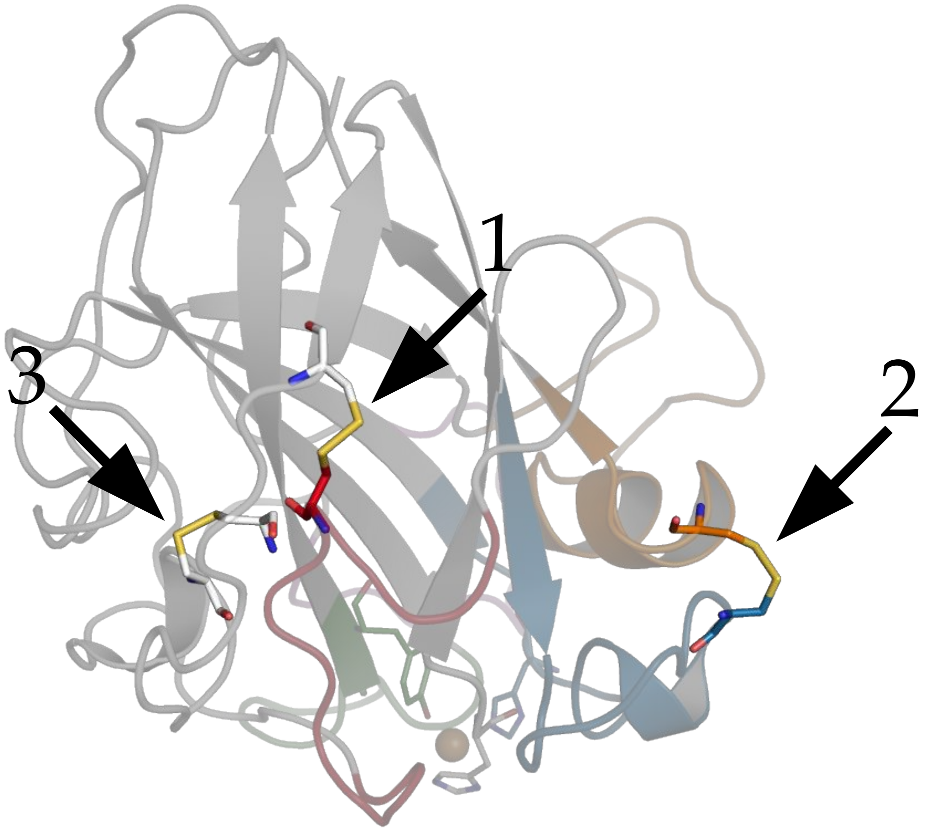

Figure S6. Cartoon representation of the homology model of AON76800.1 *Thermothelomyces thermophilus* LPMO9B. The segments are colored red (Seg1), blue (Seg2), orange (Seg3), green (Seg4) and magenta (Seg5). The disulfide bonds are highlighted by stick representation. The numbers 1–3 indicate the disulfide bonds between the residues 18–49, 38–173 and 70–133, respectively. The second disulfide bond is binding and shortening Seg2 and thereby mimicking a short Seg2. The Homology model was generated using SWISS-MODEL [4–8] (template: PDB entry 5TKF, sequence identity: 41.86%, QMEAN: -2.03).

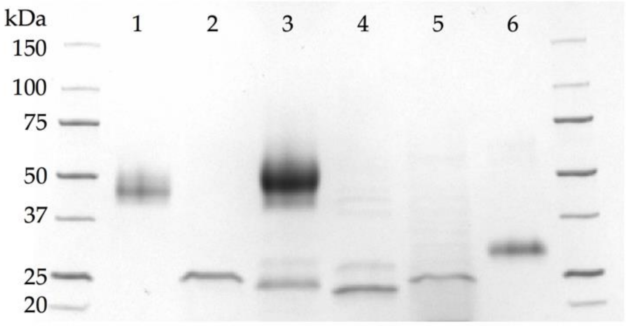

Figure S7. SDS-PAGE of purified LPMOs and LPMO variants. *Nc*LPMO9C (Lane 1), *Nc*LPMO9C$^{\Delta CBM}$ (Lane 2), *Nc*LPMO9C$^{\Delta Seg2}$ (Lane 3), *Nc*LPMO9C$^{\Delta Seg2,\ \Delta CBM}$ (Lane 4), *Nc*LPMO9F (Lane 5) and *Nc*LPMO9M (Lane 6). Lanes and molecular weights of the marker proteins (Precision Plus Protein Unstained Standards, Bio-Rad) are indicated. The precast gel (4–20% Mini-PROTEAN® TGX Stain-Free™ Precast Gel, BioRad) was stained with colloidal Coomassie blue (Bio-Safe™ Coomassie G250 Stain, BioRad) for 1 h and destained overnight.

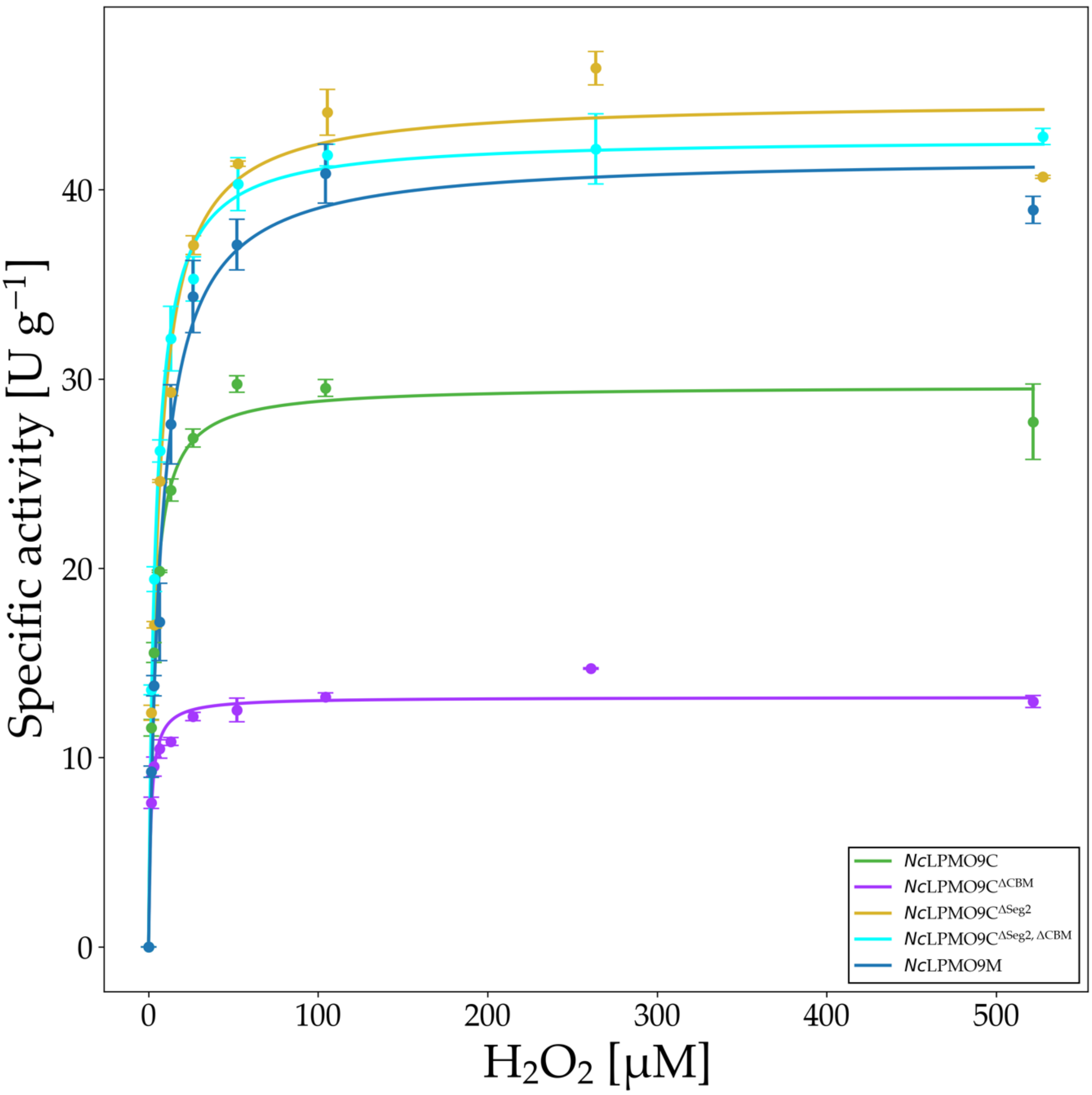

Figure S8. Determination of kinetic constants of *Nc*LPMO9M, *Nc*LPMO9C and *Nc*LPMO9C variants for H_2O_2. As substrate 2 mM 2,6-DMP in 100 mM sodium acetate buffer, pH 6.0 was used. The LPMO acitivity assay was performed as previously published [9].The calculated K_M and k_{cat} values are given in **Table 1**. Error bars show the standard deviation of three replicates.

```
(a) 1   HTIFQKVSVNGADQGQLKGIRAPANNNPVTDVMSSDIICNAVTMKDSNVLTVPAGAKVGH 60
    61  FWGHEIGGAAGPNDADNPIAASHKGPIMVYLAKVDNAATTGTSGLKWFKVAEAGLSNGKW 120
    121 AVDDLIANNGWSYFDMPTCIAPGQYLMRAELIALHNAGSQAGAQFYIGCAQINVTGGGSA 180
    181 SPSNTVSFPGAYSASDPGILINIYGGSGKTDNGGKPYQIPGPALFTCPAGGSGGSSPAPA 240
    241 TTASTPKPTSASAPKPVSTTASTPKPTNGSGSGTGAAHSTKCGGSKPAATTKASNPQPTN 300
    301 GGNSAVRAAALYGQCGGKGWTGPTSCASGTCKFSNDWYSQCLP                  343

(b) 1   HTIFQKVSVNGADQGQLKGIRAPANNNPVTDVMSSDIICNAVTMKDSNVLTVPAGAKVGH 60
    61  FWGHEIGGAAGPNDADNPIAASHKGPIMVYLAKVDNAATTGTSGLKWFKVAEAGLSNGKW 120
    121 AVDDLIANNGWSYFDMPTCIAPGQYLMRAELIALHNAGSQAGAQFYIGCAQINVTGGGSA 180
    181 SPSNTVSFPGAYSASDPGILINIYGGSGKTDNGGKPYQIPGPALFTC              227

(c) 1   HTIFQKVSVNGADQGQLKGIRAPANNNPVTDVMSSDIICNAVTMKDSNVLTVPAGAKVGH 60
    61  FWAPSVYHKGPIMVYLAKVDNAATTGTSGLKWFKVAEAGLSNGKWAVDDLIANNGWSYFD 120
    121 MPTCIAPGQYLMRAELIALHNAGSQAGAQFYIGCAQINVTGGGSASPSNTVSFPGAYSAS 180
    181 DPGILINIYGGSGKTDNGGKPYQIPGPALFTCPAGGSGGSSPAPATTASTPKPTSASAPK 240
    241 PVSTTASTPKPTNGSGSGTGAAHSTKCGGSKPAATTKASNPQPTNGGNSAVRAAALYGQC 300
    301 GGKGWTGPTSCASGTCKFSNDWYSQCLP                                 328

(d) 1   HTIFQKVSVNGADQGQLKGIRAPANNNPVTDVMSSDIICNAVTMKDSNVLTVPAGAKVGH 60
    61  FWAPSVYHKGPIMVYLAKVDNAATTGTSGLKWFKVAEAGLSNGKWAVDDLIANNGWSYFD 120
    121 MPTCIAPGQYLMRAELIALHNAGSQAGAQFYIGCAQINVTGGGSASPSNTVSFPGAYSAS 180
    181 DPGILINIYGGSGKTDNGGKPYQIPGPALFTC                             212

(e) 1   HYTFPKVWANSGTTADWQYVRRADNWQNNGFVDNVNSQQIRCFQSTHSPAQSTLSVAAGT 60
    61  TITYGAAPSVYHPGPMQFYLARVPDGQDINSWTGEGAVWFKIYHEQPTFGSQLTWSSNGK 120
    121 SSFPVKIPSCIKSGSYLLRAEHIGLHVAQSSGAAQFYISCAQLSITGGGSTEPGANYKVS 180
    181 FPGAYKASDPGILININYPVPTSYKNPGPSVFTC                           214

(f) 1   HGFVDNATIGGQFYQFYQPYQDPYMGSPPDRISRKIPGNGPVEDVTSLAIQCNADSAPAK 60
    61  LHASAAAGSTVTLRWTIWPDSHVGPVITYMARCPDTGCQDWTPSASDKVWFKIKEGGREG 120
    121 TSNVWAATPLMTAPANYEYAIPSCLKPGYYLVRHEIIALHSAYSYPGAQFYPGCHQLQVT 180
    181 GSGTKTPSSGLVSFPGAYKSTDPGVTYDAYQAATYTIPGPAVFTC                225
```

Figure S9. Analysis of *Nc*LPMO variants by liquid chromatography-electrospray ionization mass spectrometry (LC-ESI-MS) confirmed their mutations. Section a, *Nc*LPMO9C; Section b, *Nc*LPMO9C$^{\Delta CBM}$; Section c, *Nc*LPMO9C$^{\Delta Seg2}$; Section d, *Nc*LPMO9C$^{\Delta Seg2,\ \Delta CBM}$, Section e *Nc*LPMO9F and Section f, *Nc*LPMO9M. Sequence sections colored in red were identified by the software Global Proteome Machine (GPM) by performing MS/MS ion searches against a homemade database containing the host cell proteome and all target sequences. Amino acids marked in green are marked as "unlikely to find" due to too short or too long peptides (or glycosylation).

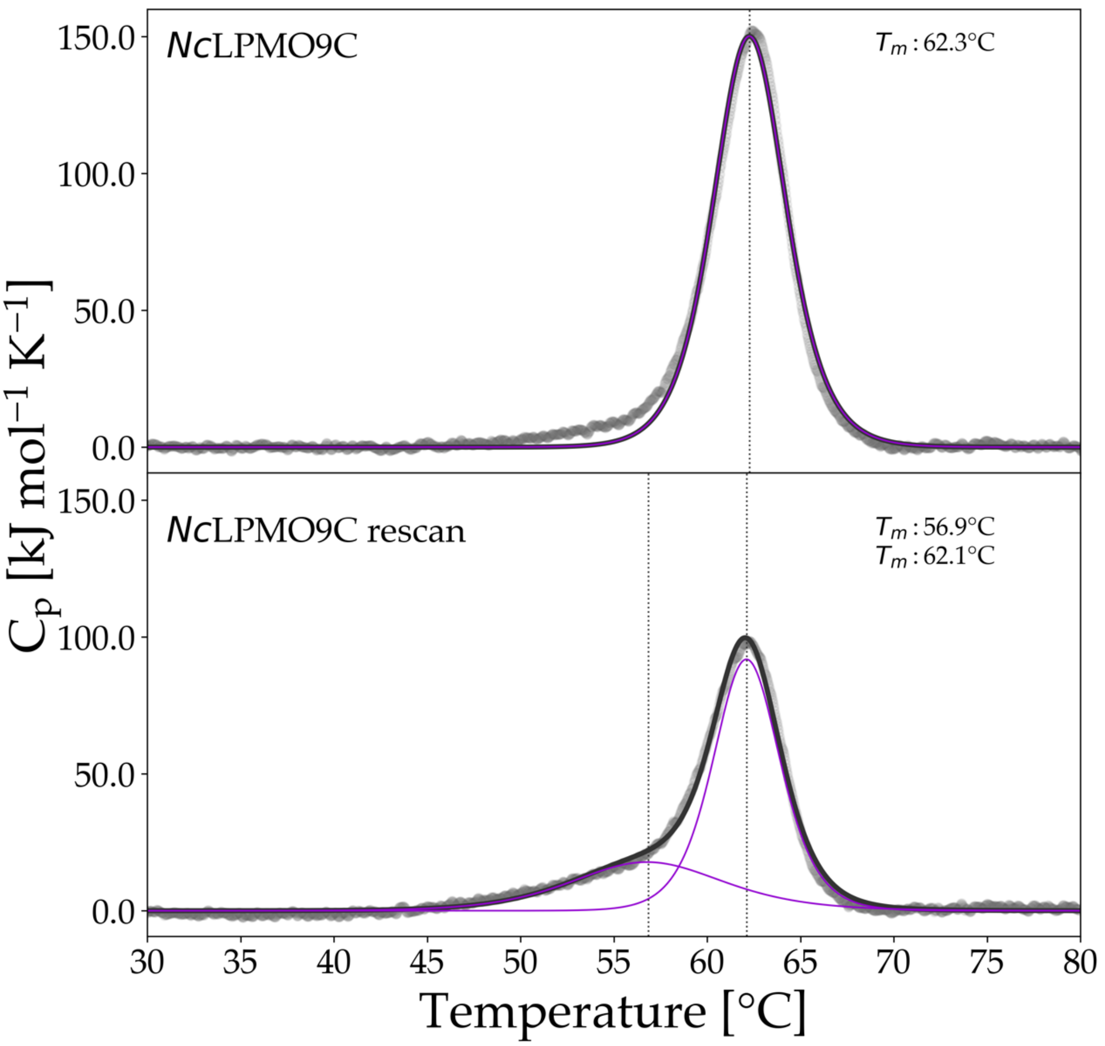

Figure S10. Differential scanning calorimetry (DSC) thermograms of the scan and rescan of *Nc*LPMO9C. Baseline subtracted raw data are shown as a grey scatter plot and the fitted curve is shown in black. The fitted curves are shown as a purple line and the transition midpoint temperature (Tm) is indicated by a vertical dotted line. Starting the scan at 20 °C, a temperature ramp of 1 K min^{-1} was applied until reaching 90 °C. For the rescan, the sample was cooled down with a temperature ramp of 1 K min^{-1} immediately after reaching 90°C and when reaching 20°C the rescan was started without delay. A 15 µM *Nc*LPMO9C concentration in 50 mM potassium phosphate buffer, pH 6.0 was applied to the instruments measurement cell.

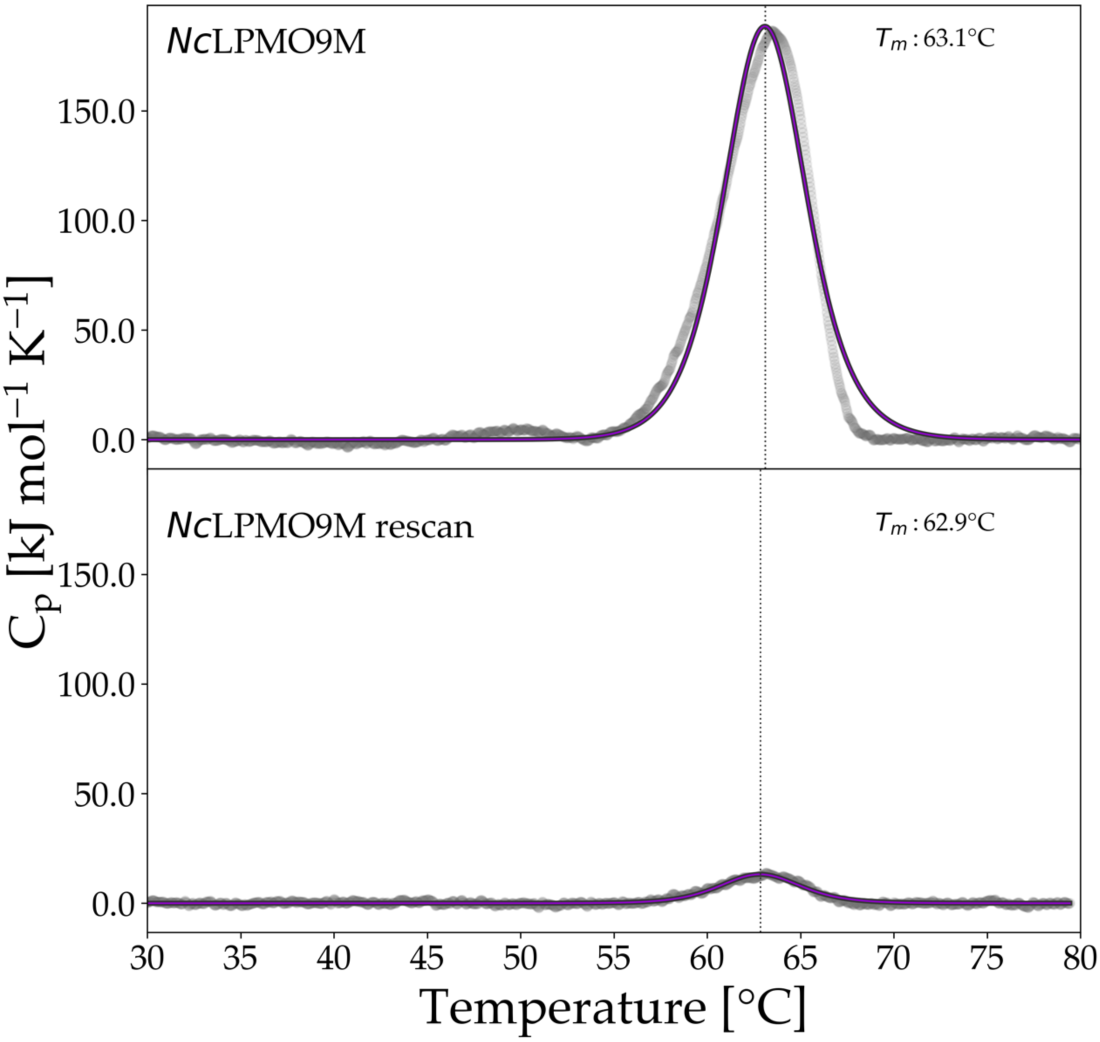

Figure S11. DSC thermograms of the scan and rescan of *Nc*LPMO9M. Baseline subtracted raw data are shown as a grey scatter plot and the fitted curve is shown in black. The fitted T_m curves are shown as a purple line and the transition midpoint temperatures are indicated by a vertical dotted line. Starting the scan at 20 °C, a temperature ramp of 1 K min^{-1} was applied until reaching 90 °C. For the rescan, the sample was cooled down with a temperature ramp of 1 K min^{-1} immediately after reaching 90°C and when reaching 20°C the rescan was started without delay. A 15 µM *Nc*LPMO9C concentration in 50 mM potassium phosphate buffer, pH 6.0 was applied to the instruments measurement cell.

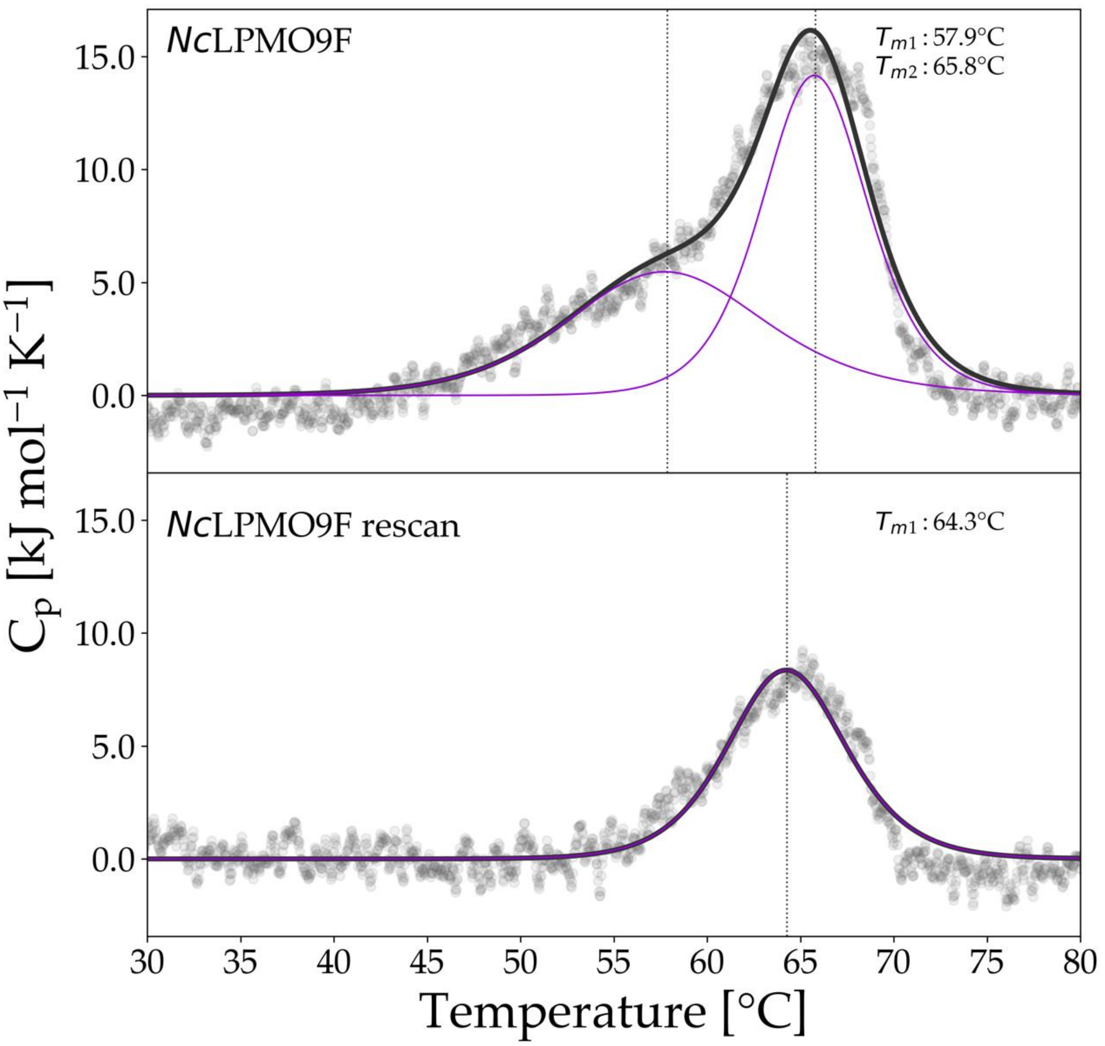

Figure S12. DSC thermograms of the scan and rescan of *Nc*LPMO9F. Baseline subtracted raw data are shown as a grey scatter plot and the fitted curve is shown in black. The fitted T_m curves are shown as a purple line and the transition midpoint temperatures are indicated by a vertical dotted line. Starting the scan at 20 °C, a temperature ramp of 1 K min^{-1} was applied until reaching 90 °C. For the rescan, the sample was cooled down with a temperature ramp of 1 K min^{-1} immediately after reaching 90°C and when reaching 20°C the rescan was started without delay. A 15 µM *Nc*LPMO9C concentration in 50 mM potassium phosphate buffer, pH 6.0 was applied to the instruments measurement cell.

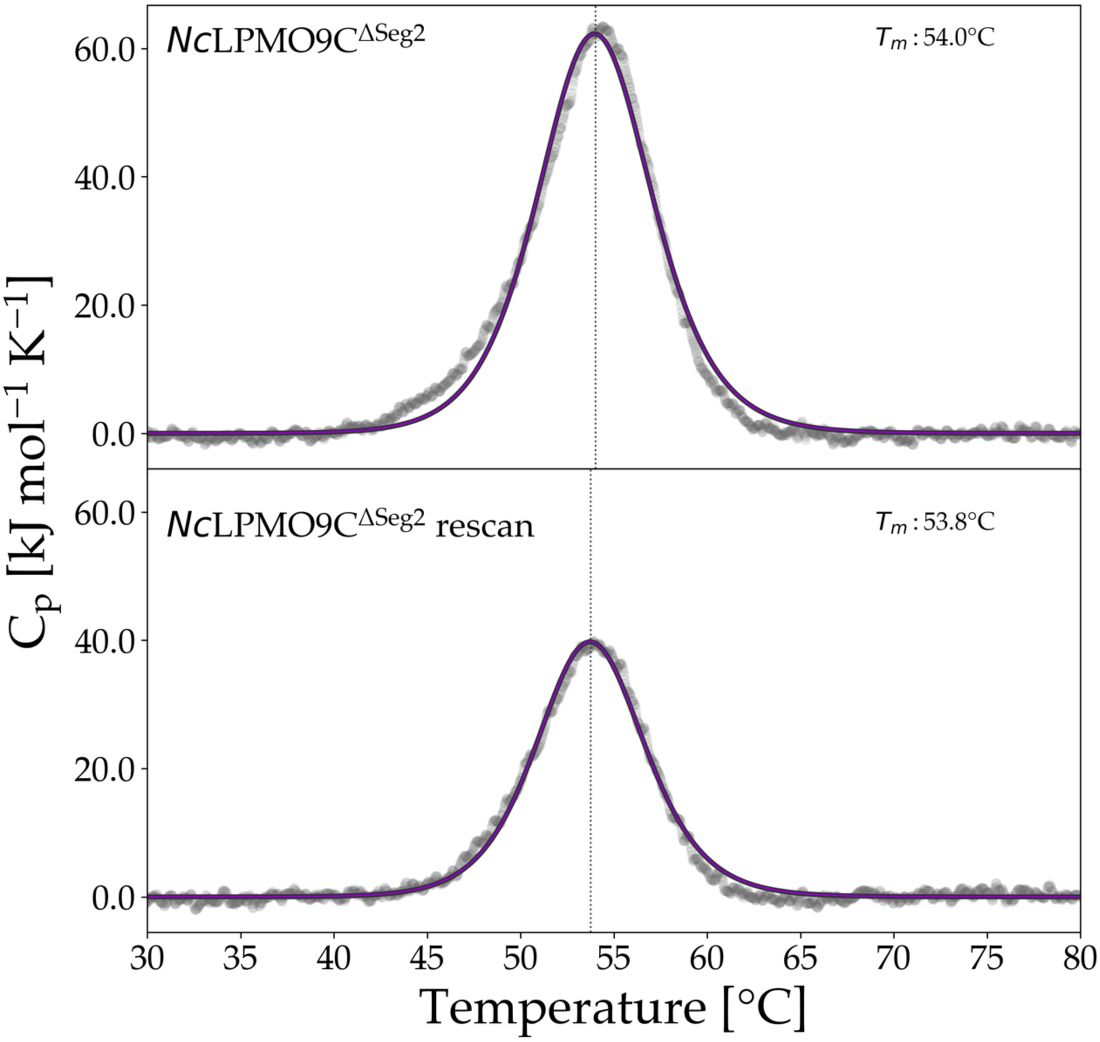

Figure S13. DSC thermograms of the scan and rescan of *Nc*LPMO9$^{\Delta Seg2}$. Baseline subtracted raw data are shown as a grey scatter plot and the fitted curve is shown in black. The fitted T_m curves are shown as a purple line and the transition midpoint temperatures are indicated by a vertical dotted line. Starting the scan at 20 °C, a temperature ramp of 1 K min^{-1} was applied until reaching 90 °C. For the rescan, the sample was cooled down with a temperature ramp of 1 K min^{-1} immediately after reaching 90°C and when reaching 20°C the rescan was started without delay. A 15 µM *Nc*LPMO9C concentration in 50 mM potassium phosphate buffer, pH 6.0 was applied to the instruments measurement cell.

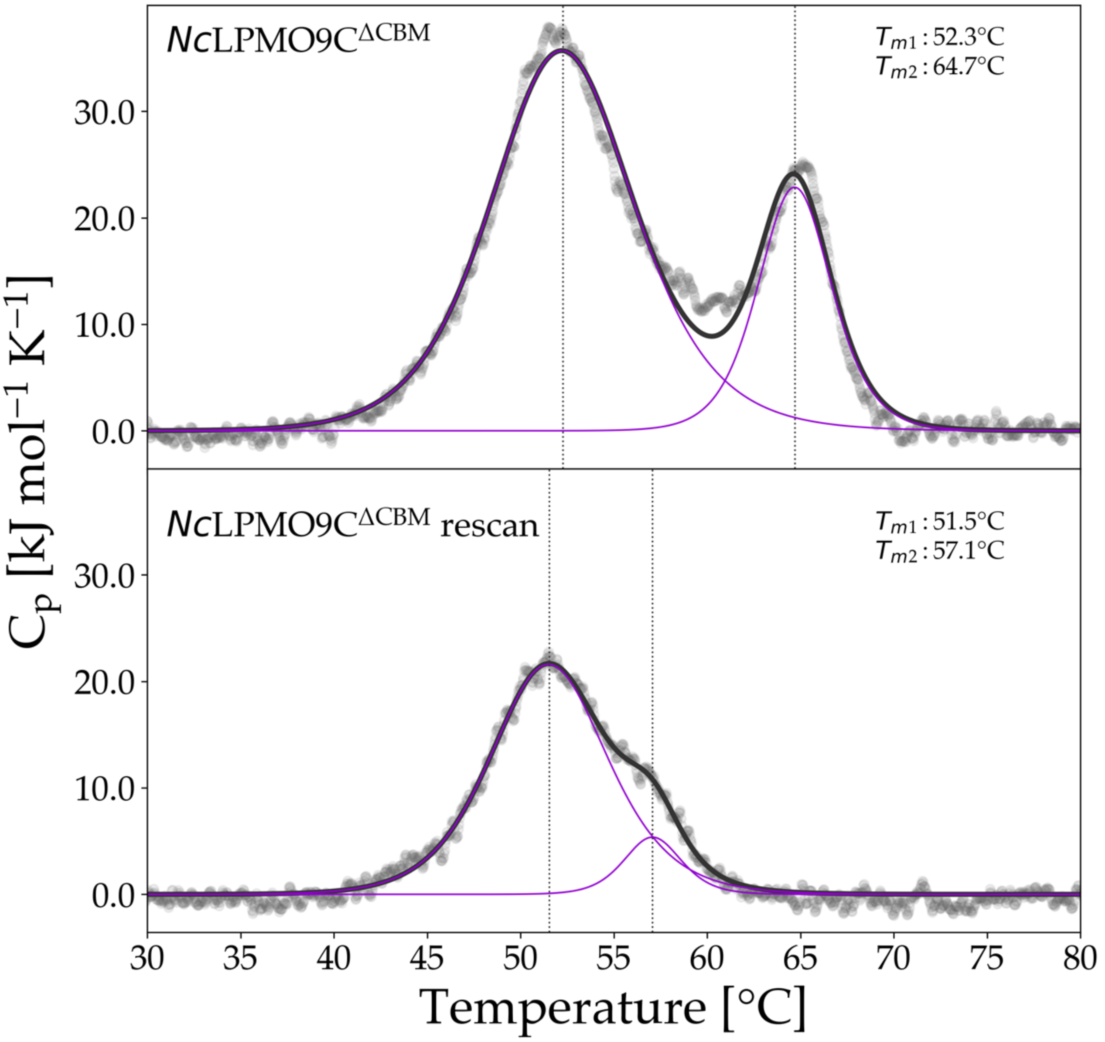

Figure S14. DSC thermograms of the scan and rescan of *Nc*LPMO9C$^{\Delta CBM}$. Baseline subtracted raw data are shown as a grey scatter plot and the fitted curve is shown in black. The fitted T_m curves are shown as a purple line and the transition midpoint temperatures are indicated by a vertical dotted line. Starting the scan at 20 °C, a temperature ramp of 1 K min^{-1} was applied until reaching 90 °C. For the rescan, the sample was cooled down with a temperature ramp of 1 K min^{-1} immediately after reaching 90°C and when reaching 20°C the rescan was started without delay. A 15 µM *Nc*LPMO9C concentration in 50 mM potassium phosphate buffer, pH 6.0 was applied to the instruments measurement cell.

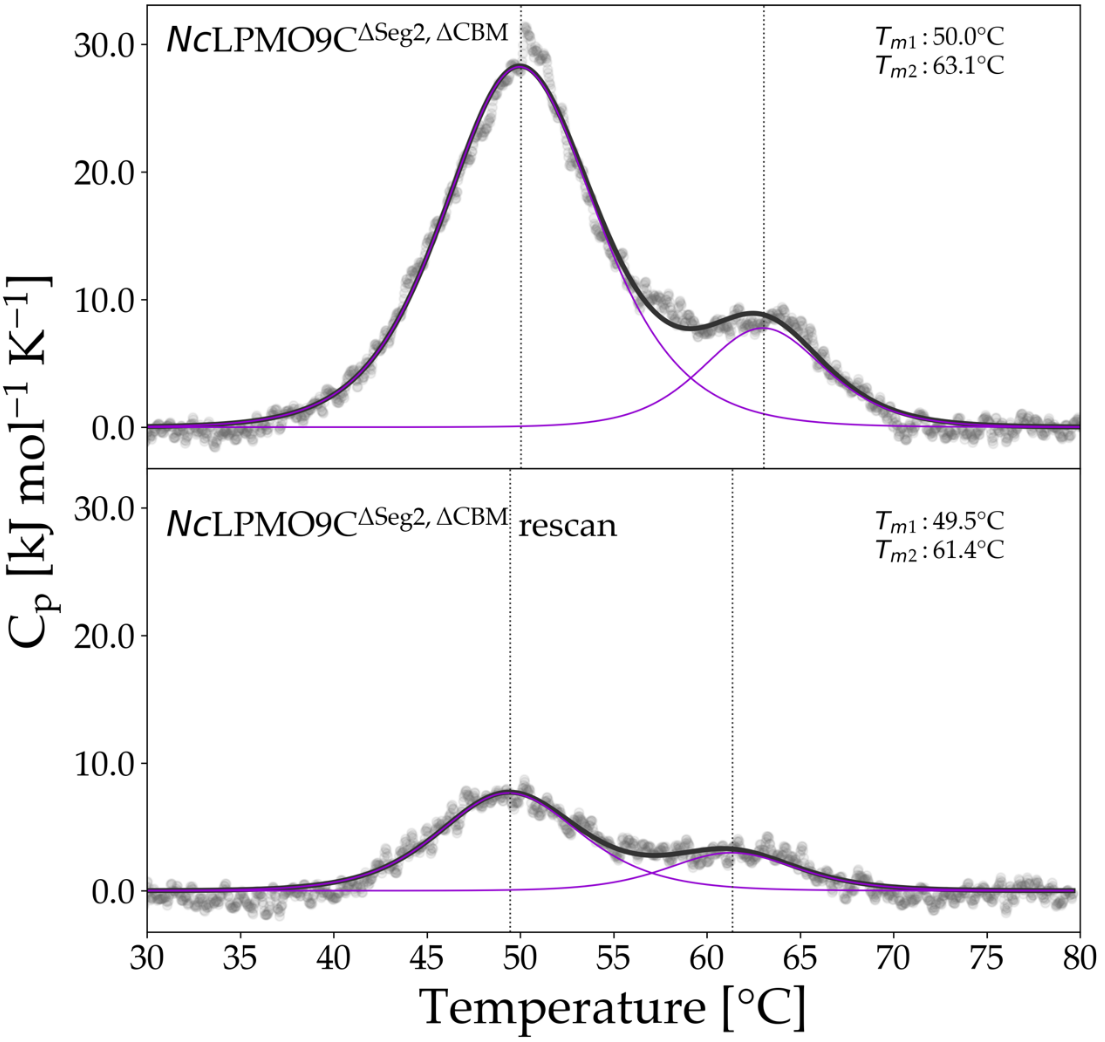

Figure S15. DSC thermograms of the scan and rescan of *Nc*LPMO9C$^{\Delta Seg2,\ \Delta CBM}$. Baseline subtracted raw data are shown as a grey scatter plot and the fitted curve is shown in black. The fitted T_m curves are shown as a purple line and the transition midpoint temperatures are indicated by a vertical dotted line. Starting the scan at 20 °C, a temperature ramp of 1 K min^{-1} was applied until reaching 90 °C. For the rescan, the sample was cooled down with a temperature ramp of 1 K min^{-1} immediately after reaching 90°C and when reaching 20°C the rescan was started without delay. A 15 µM *Nc*LPMO9C concentration in 50 mM potassium phosphate buffer, pH 6.0 was applied to the instruments measurement cell.

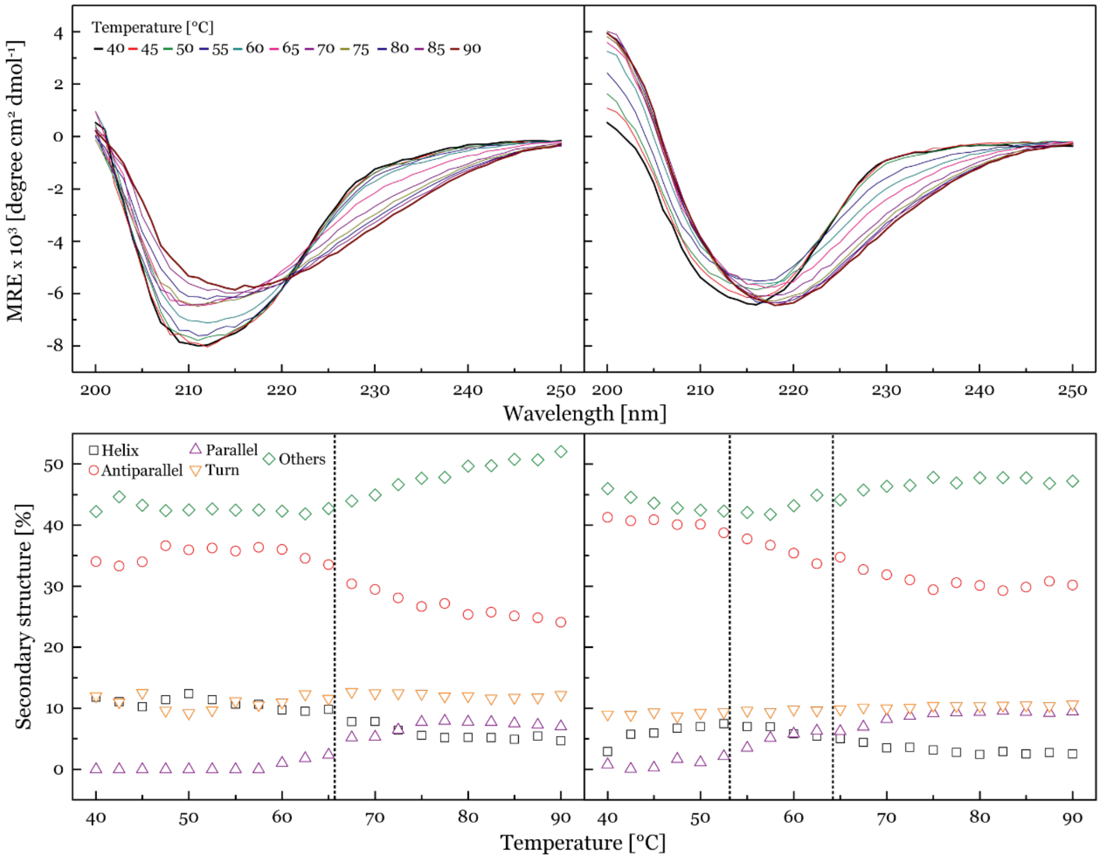

Figure S16. Monitoring of thermal denaturation by circular dichroism (CD). ECD spectra of 3 mg mL^{-1} *Nc*LPMO9C (left panels) and *Nc*LPMO9C$^{\Delta CBM}$ (right panels) at different temperatures (top panels) for a wavelength range between 200–250 nm. Using the BestSel web server (http://bestsel.elte.hu) the secondary structure was predicted for all different temperatures (lower panels) [11].

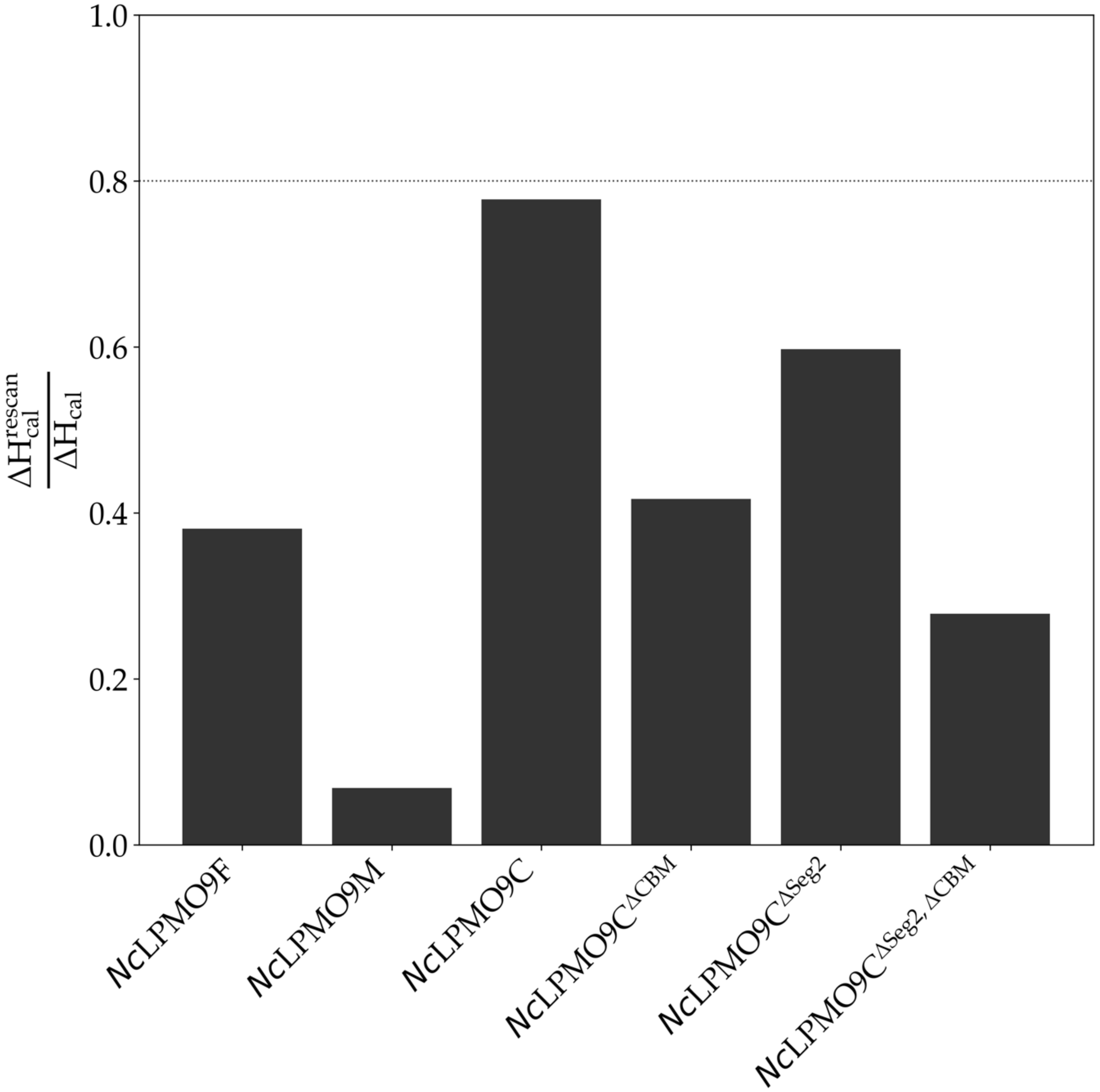

Figure S17. Bar plot showing the ratio of ΔH_{cal} of the DSC rescan and ΔH_{cal} of the first scan. This ratio is used to asses possible refolding of the protein [10]. The horizontal line indicates the threshold for reversible unfolding. Data were calculated from the thermograms shown in Supplementary Figures S10-S15.

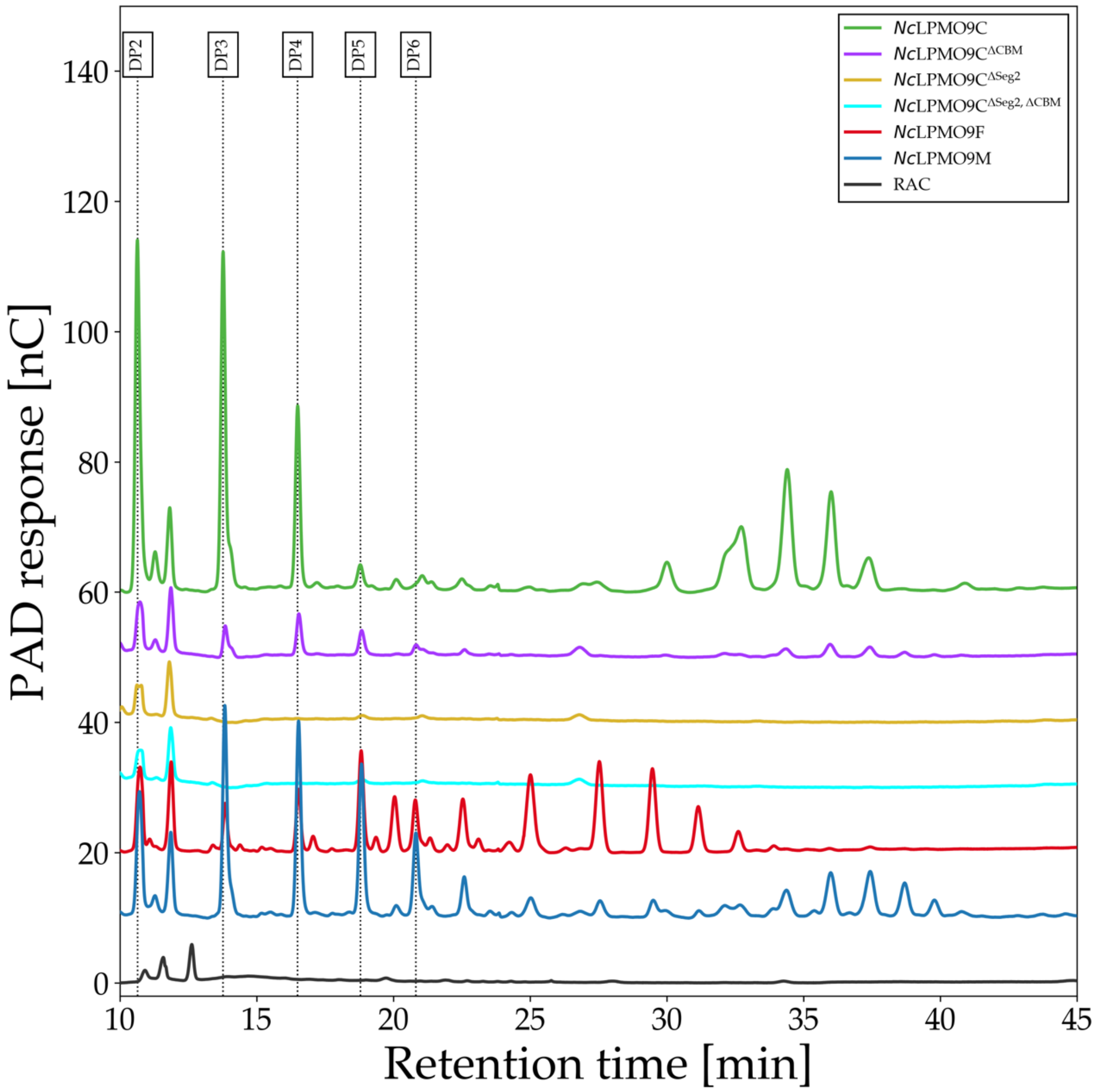

Figure S18. HPAEC chromatogram of products released from the reaction of 2 g L^{-1} RAC with 1.25 µM of the indicated LPMOs in the presence of 1 mM ascorbic acid after 24 h of incubation at 30 °C in 50 mM ammonium acetate buffer, pH 5.5. The data are baseline corrected using a baseline detection algorithm with a polynomial of the 3rd degree and a tolerance of 10^{-14} [12].

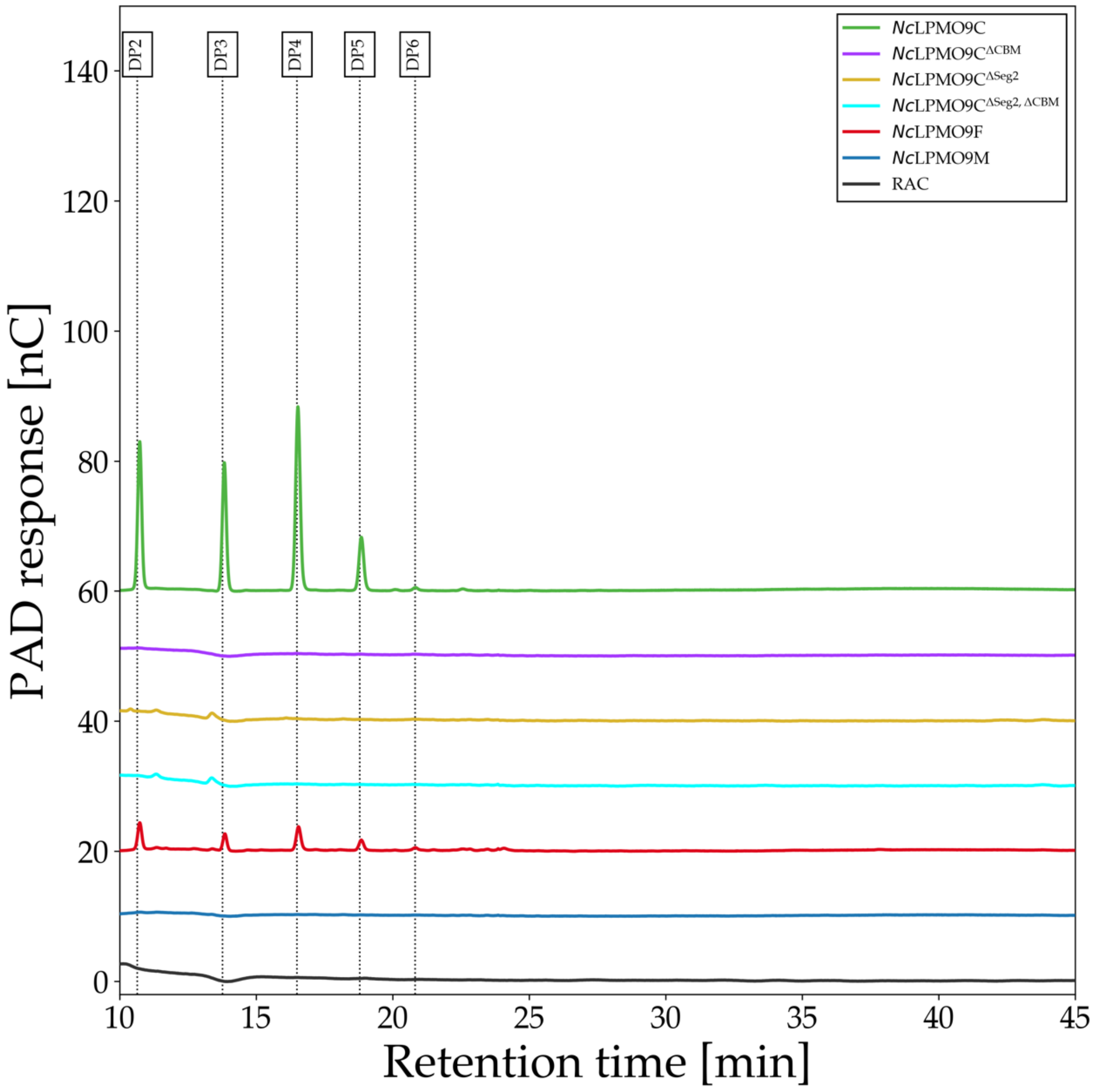

Figure S19. HPAEC chromatogram of products released from the reaction of 2 g L^{-1} RAC with 1.25 µM of the indicated LPMOs in the absence of ascorbic acid after 24 h of incubation at 30 °C in 50 mM ammonium acetate buffer, pH 5.5. The data are baseline corrected using a baseline detection algorithm with a polynomial of the 3rd degree and a tolerance of 10^{-14} [12].

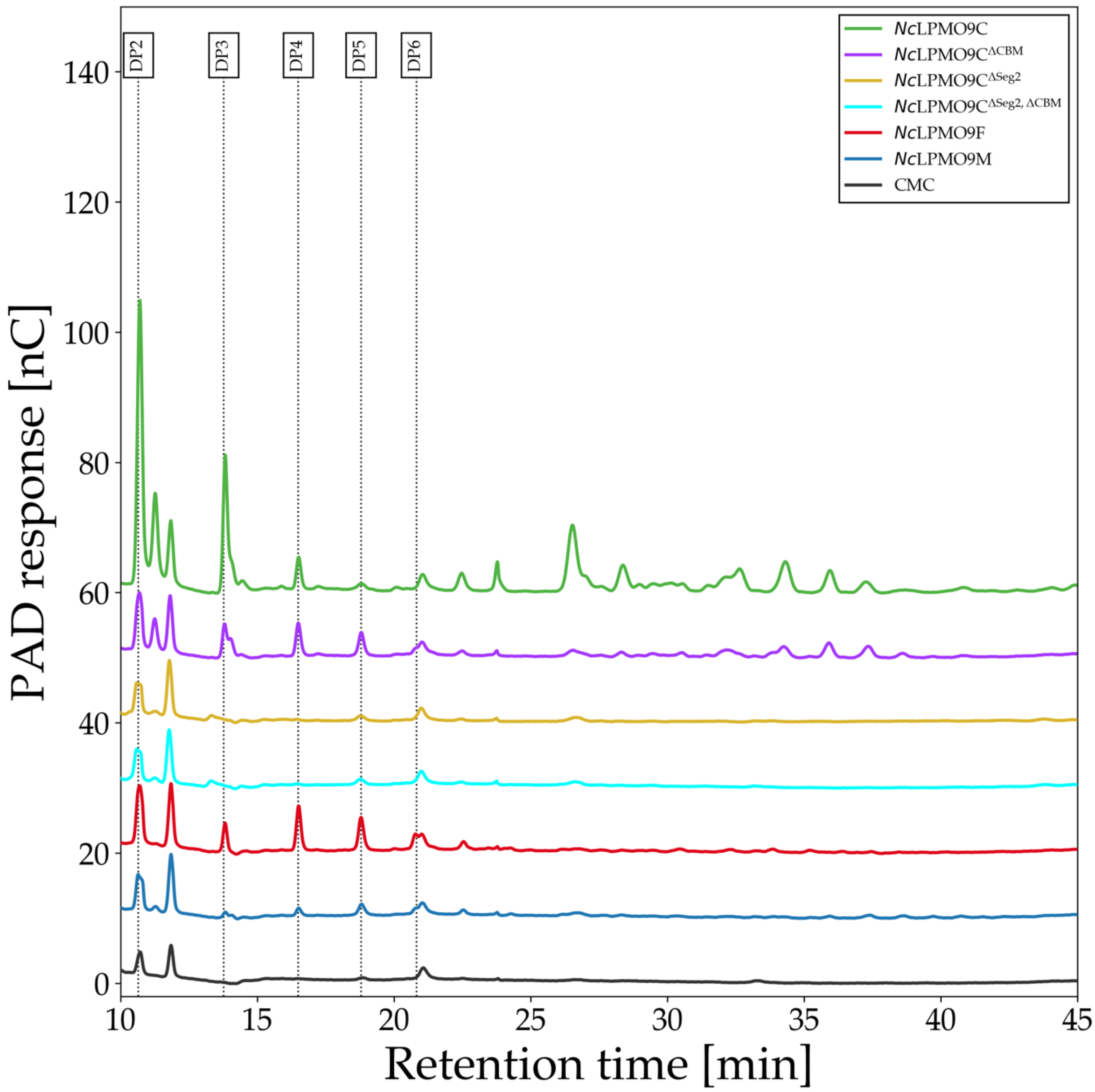

Figure S20. HPAEC chromatogram of products released from the reaction of 2 g L^{-1} CMC with 1.25 µM of the indicated LPMOs in the presence of 1 mM ascorbic acid after 24 h of incubation at 30 °C in 50 mM ammonium acetate buffer, pH 5.5. The data are baseline corrected using a baseline detection algorithm with a polynomial of the 3rd degree and a tolerance of 10^{-14} [12].

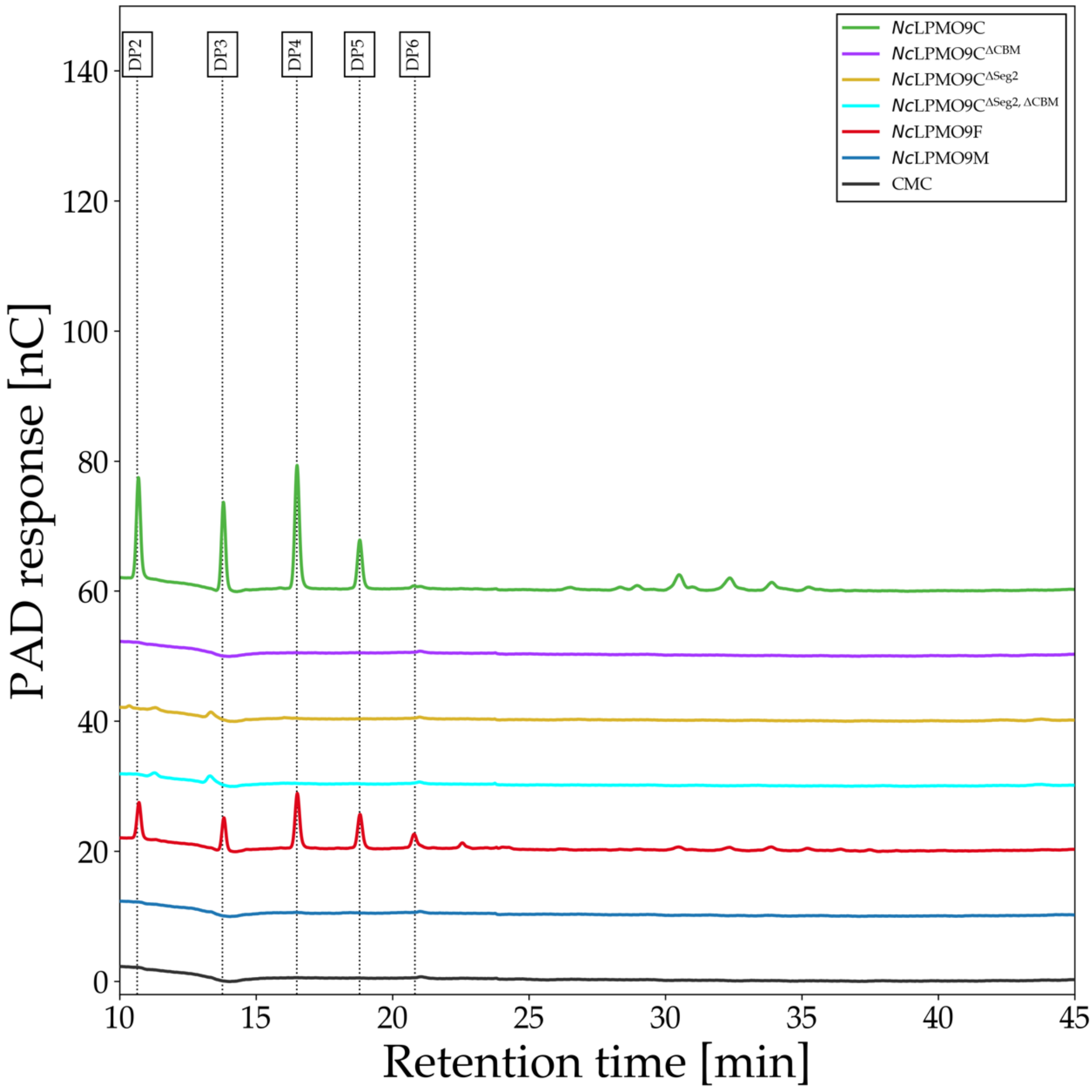

Figure S21. HPAEC chromatogram of products released from the reaction of 2 g L^{-1} CMC with 1.25 µM of the indicated LPMOs in the absence of ascorbic acid after 24 h of incubation at 30 °C in 50 mM ammonium acetate buffer, pH 5.5. The data are baseline corrected using a baseline detection algorithm with a polynomial of the 3rd degree and a tolerance of 10^{-14} [12].

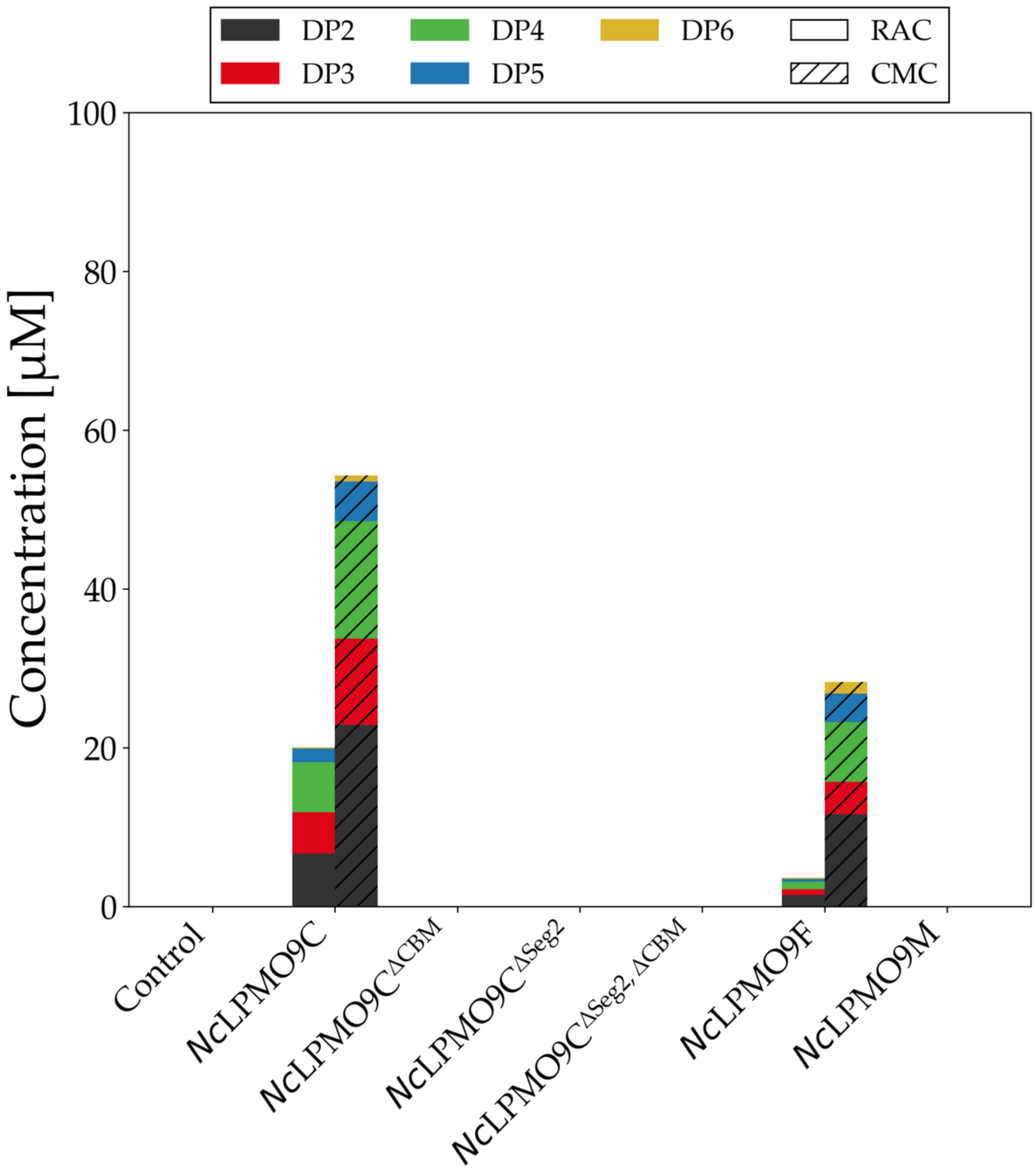

Figure S22. Quantification of cellodextrins in the supernatant released in a batch conversion by 1.25 µM of LPMO and LPMO variants after a 24 h incubation at 30 °C in 50 mM ammonium acetate buffer, pH 5.5 on 2 g L^{-1} RAC or CMC in the absence of ascorbic acid. The integration windows for product quantitation were defined as described in Supplementary **Table S3**.

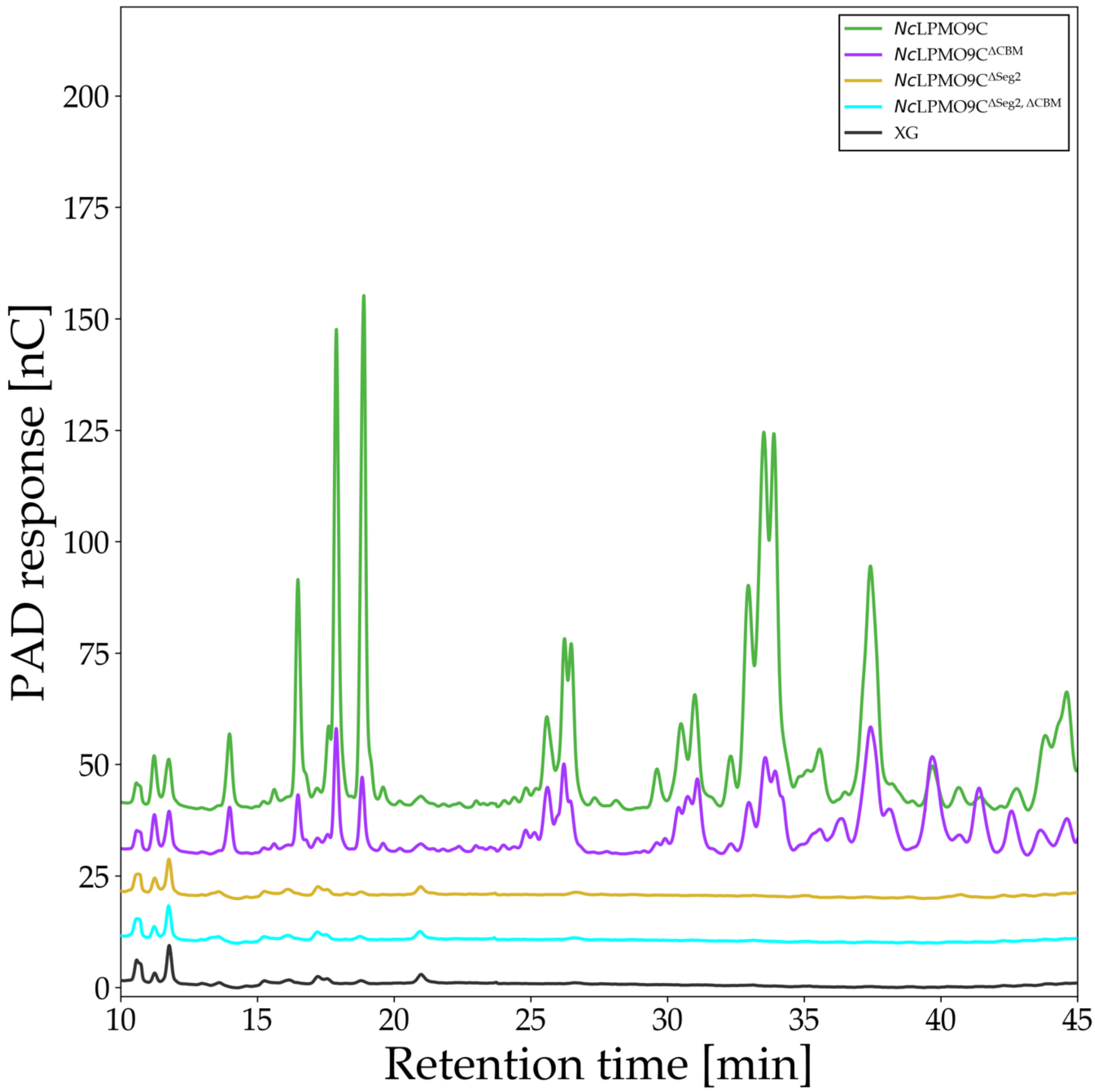

Figure S23. HPAEC chromatogram of Xylogluco-oligomer products released from the reaction of 2 g L^{-1} XG with 1.25 µM of the indicated LPMOs in the presence of 1 mM ascorbic acid after 24 h of incubation at 30 °C in 50 mM ammonium acetate buffer, pH 5.5. The data are baseline corrected using a baseline detection algorithm with a polynomial of the 3rd degree and a tolerance of 10^{-14} [12].

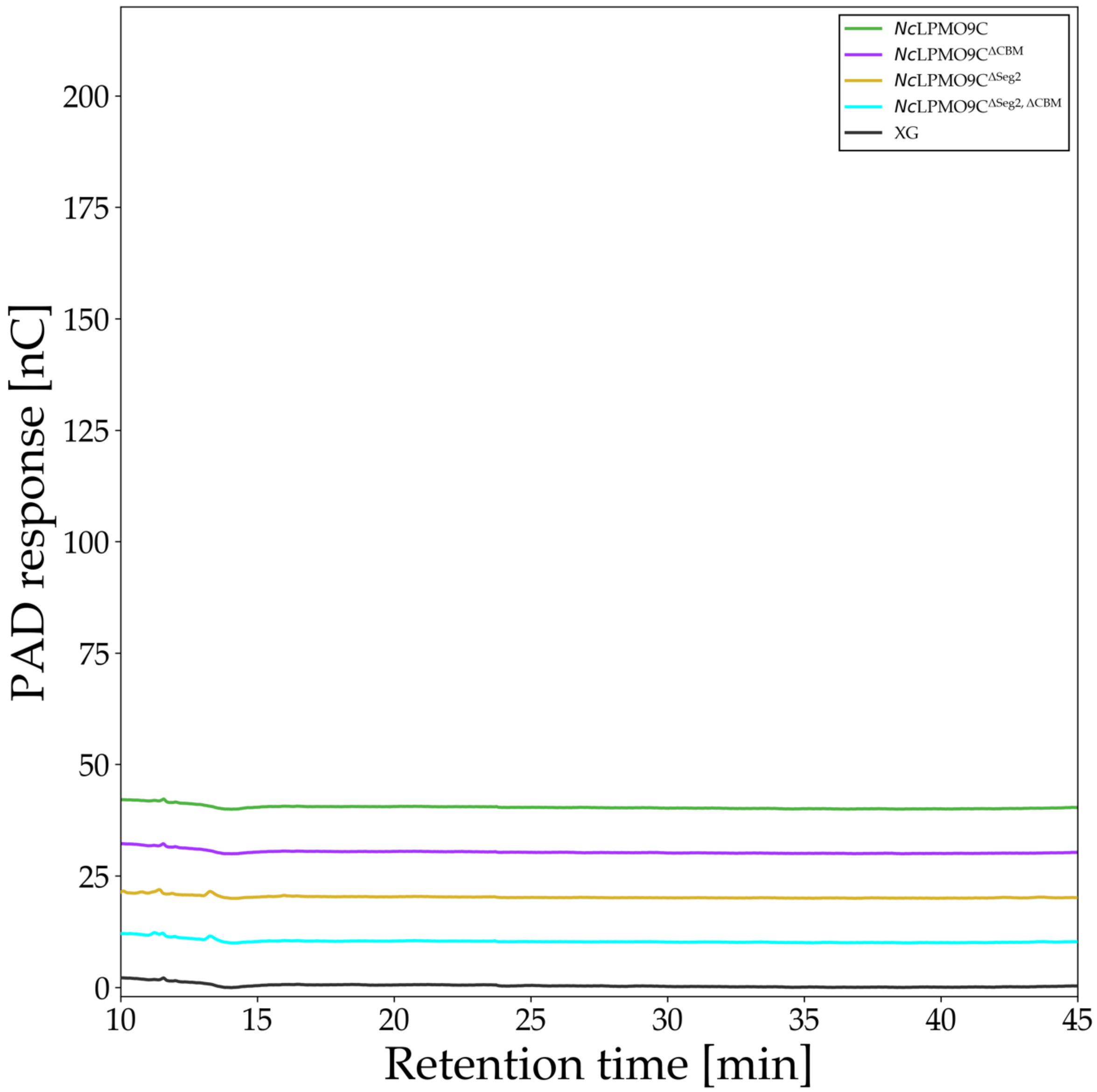

Figure S24. HPAEC chromatogram of Xylogluco-oligomer products released from the reaction of 2 g L^{-1} XG with 1.25 μM of the indicated LPMOs in the absence of ascorbic acid after 24 h of incubation at 30 °C in 50 mM ammonium acetate buffer, pH 5.5. The data are baseline corrected using a baseline detection algorithm with a polynomial of the 3rd degree and a tolerance of 10^{-14} [12].

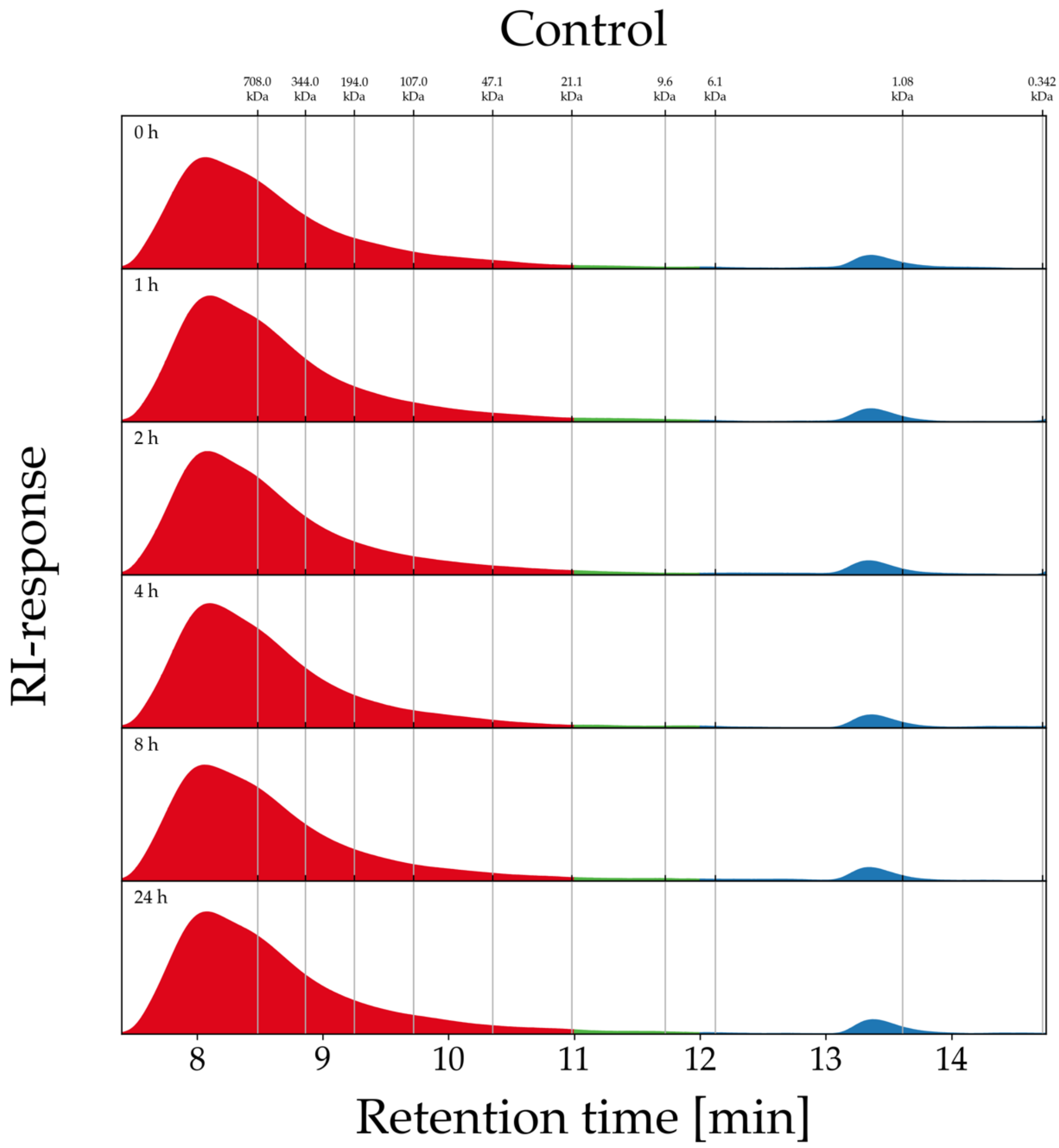

Figure S25. HPSEC chromatogram of products released from the reaction of 2 g L^{-1} XG without LPMO in the absence ascorbic acid after 24 h of incubation at 30 °C in 50 mM ammonium acetate buffer, pH 5.5. The data are baseline corrected using a baseline detection algorithm with a polynomial of the 2nd degree and a tolerance of 10^{-14} [12]

.

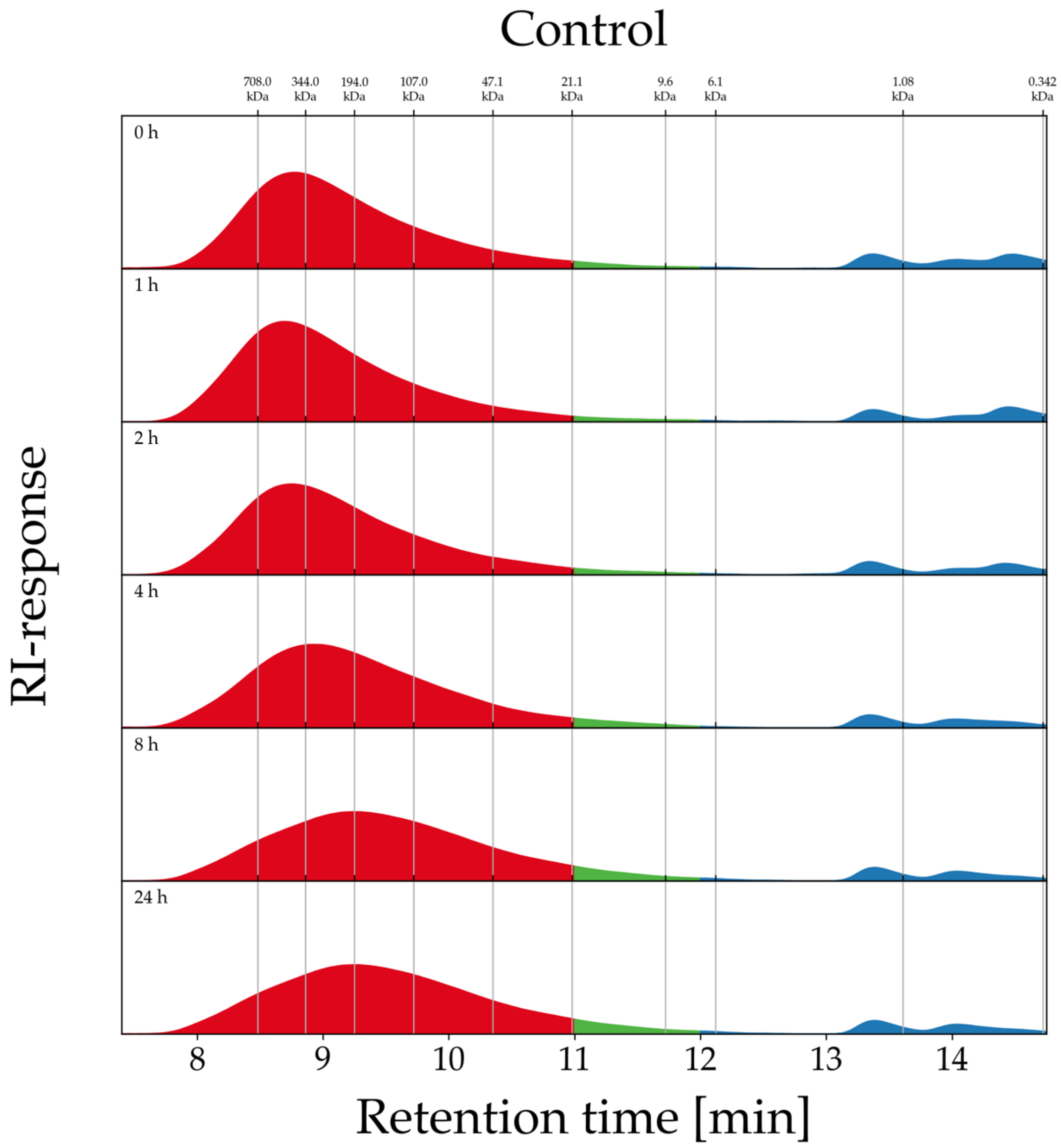

Figure S26. HPSEC chromatogram of products released from the reaction of 2 g L^{-1} XG without LPMO in the presence of 1 mM ascorbic acid after 24 h of incubation at 30 °C in 50 mM ammonium acetate buffer, pH 5.5. The data are baseline corrected using a baseline detection algorithm with a polynomial of the 2nd degree and a tolerance of 10^{-14} [12].

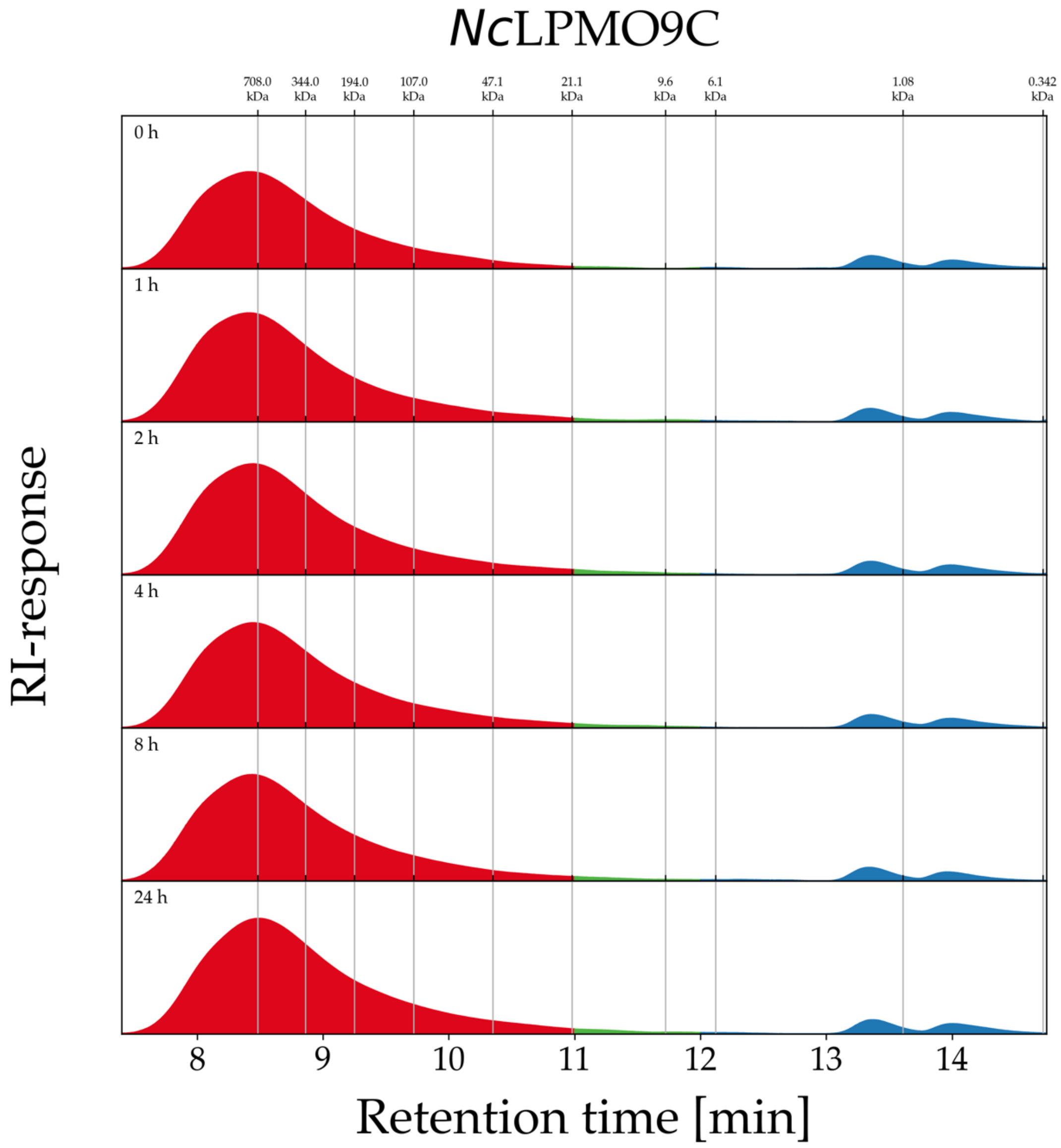

Figure S27. HPSEC chromatogram of products released from the reaction of 2 g L^{-1} XG with 1.25 µM *Nc*LPMO9C in the absence ascorbic acid after 24 h of incubation at 30 °C in 50 mM ammonium acetate buffer, pH 5.5. The data are baseline corrected using a baseline detection algorithm with a polynomial of the 2nd degree and a tolerance of 10^{-14} [12].

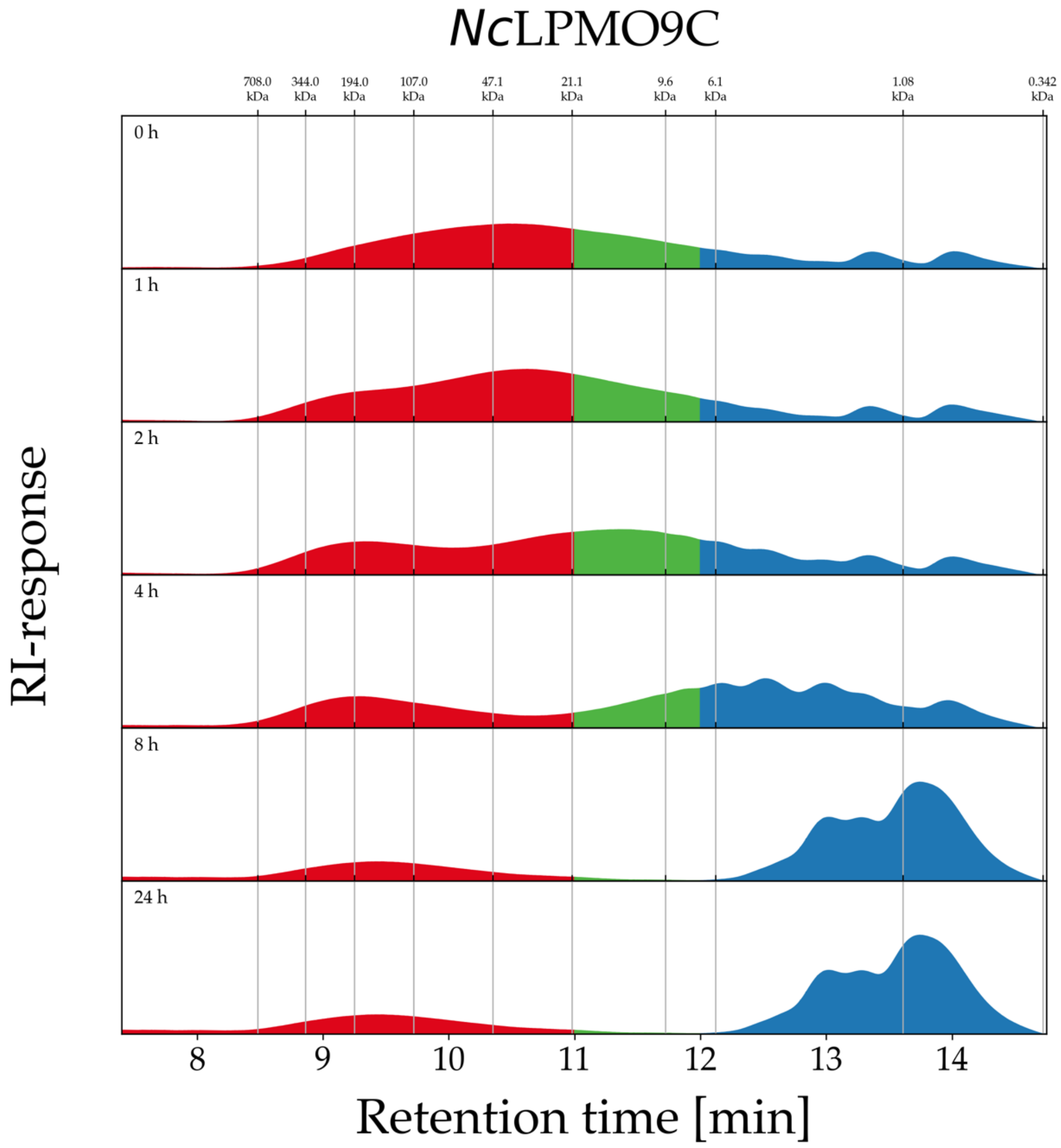

Figure S28. HPSEC chromatogram of products released from the reaction of 2 g L^{-1} XG with 1.25 µM *Nc*LPMO9C in the presence of 1 mM ascorbic acid after 24 h of incubation at 30 °C in 50 mM ammonium acetate buffer, pH 5.5. The data are baseline corrected using a baseline detection algorithm with a polynomial of the 2nd degree and a tolerance of 10^{-14} [12].

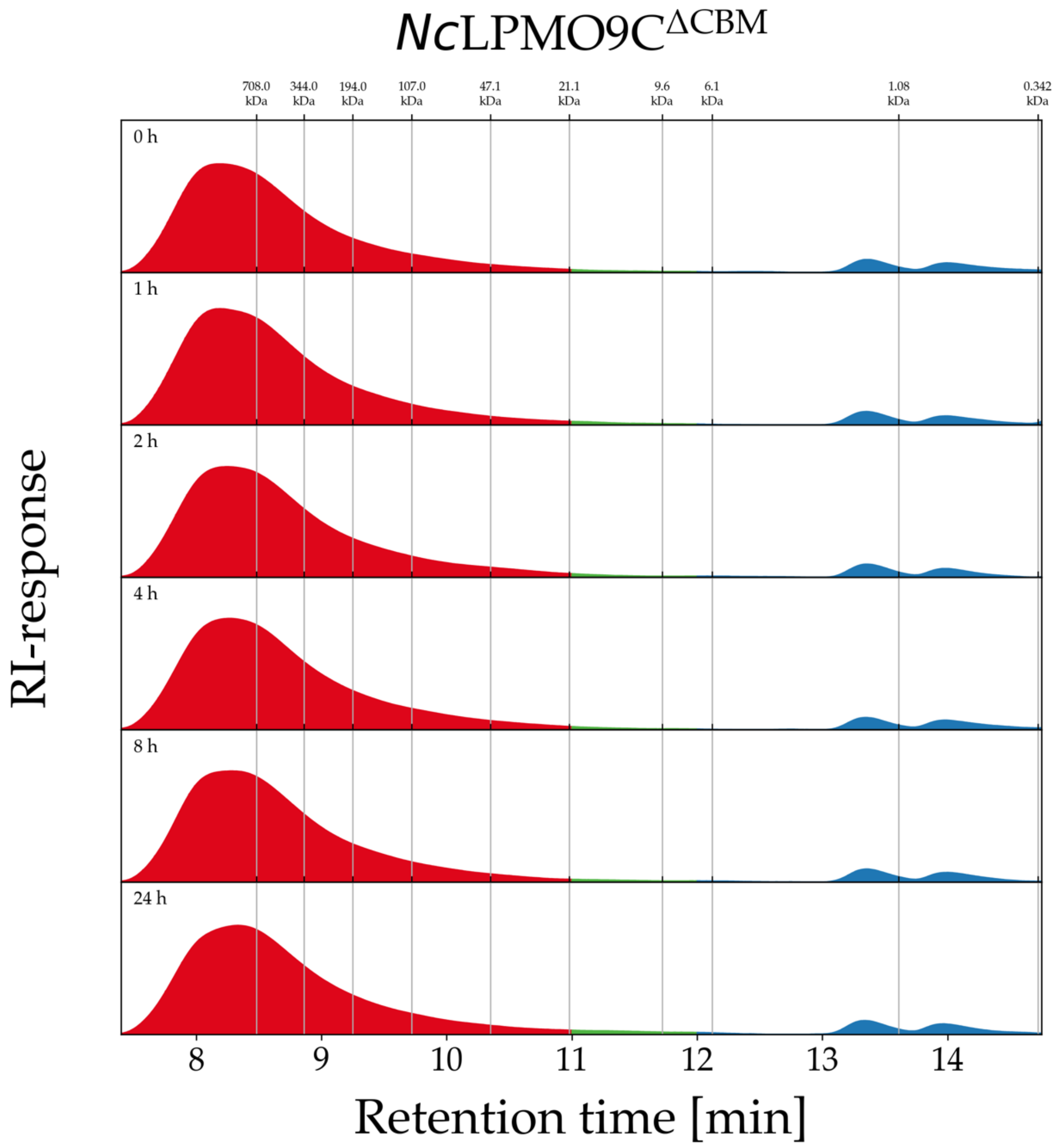

Figure S29. HPSEC chromatogram of products released from the reaction of 2 g L^{-1} XG with 1.25 µM *Nc*LPMO9C$^{\Delta CBM}$ in the absence ascorbic acid after 24 h of incubation at 30 °C in 50 mM ammonium acetate buffer, pH 5.5. The data are baseline corrected using a baseline detection algorithm with a polynomial of the 2nd degree and a tolerance of 10^{-14} [12].

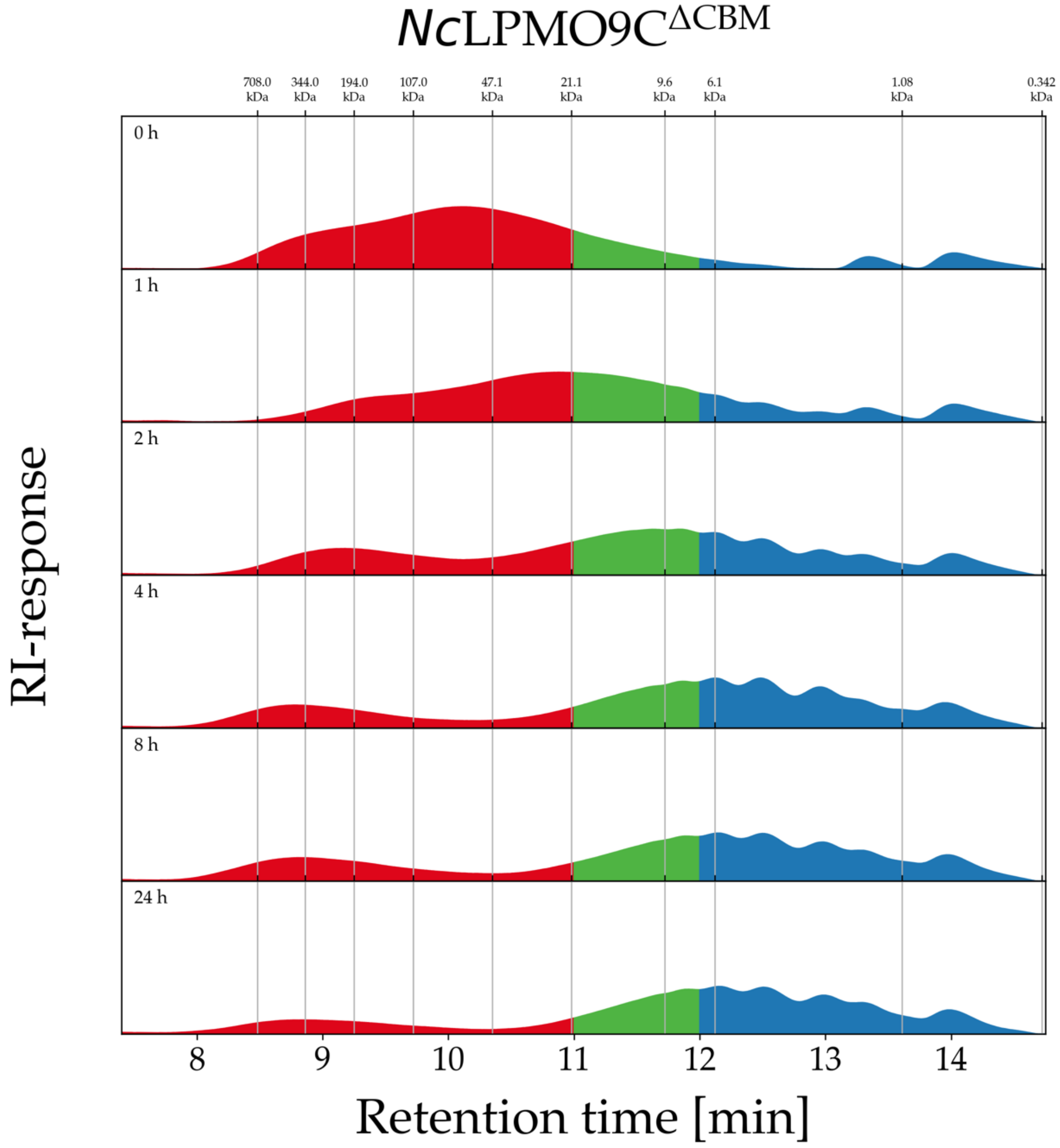

Figure S30. HPSEC chromatogram of products released from the reaction of 2 g L^{-1} XG with 1.25 µM *Nc*LPMO9C$^{\Delta CBM}$ in the presence of 1 mM ascorbic acid after 24 h of incubation at 30 °C in 50 mM ammonium acetate buffer, pH 5.5. The data are baseline corrected using a baseline detection algorithm with a polynomial of the 2nd degree and a tolerance of 10^{-14} [12].

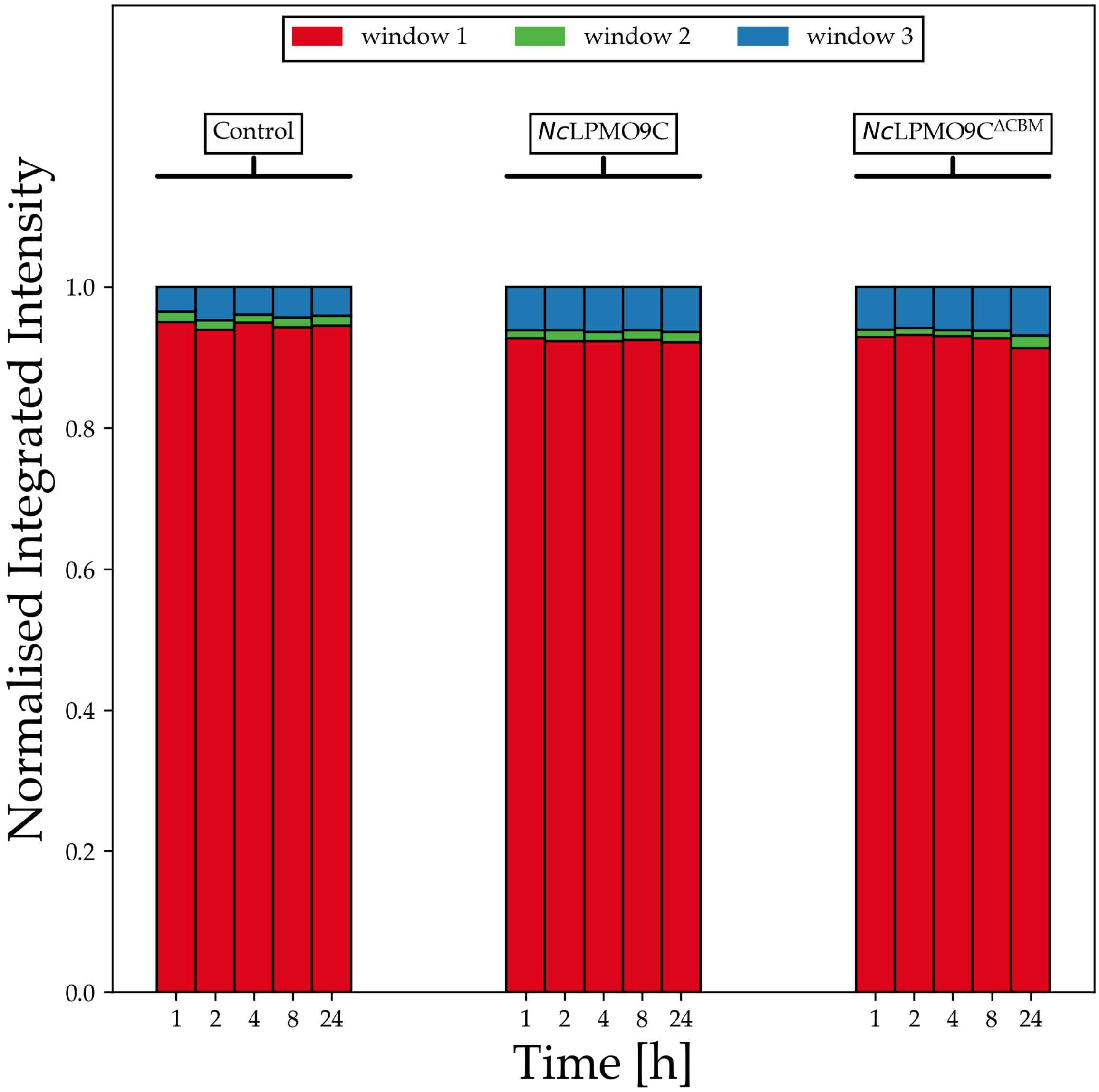

Figure S31. Normalized integrated intensity of negative controls without ascorbic acid. The area under the curves from **Figures S27-29** were integrated. The integrated peaks within the elution windows as described in Supplementary **Table S4** are showing no hydrolytic background of the LPMO preparations.

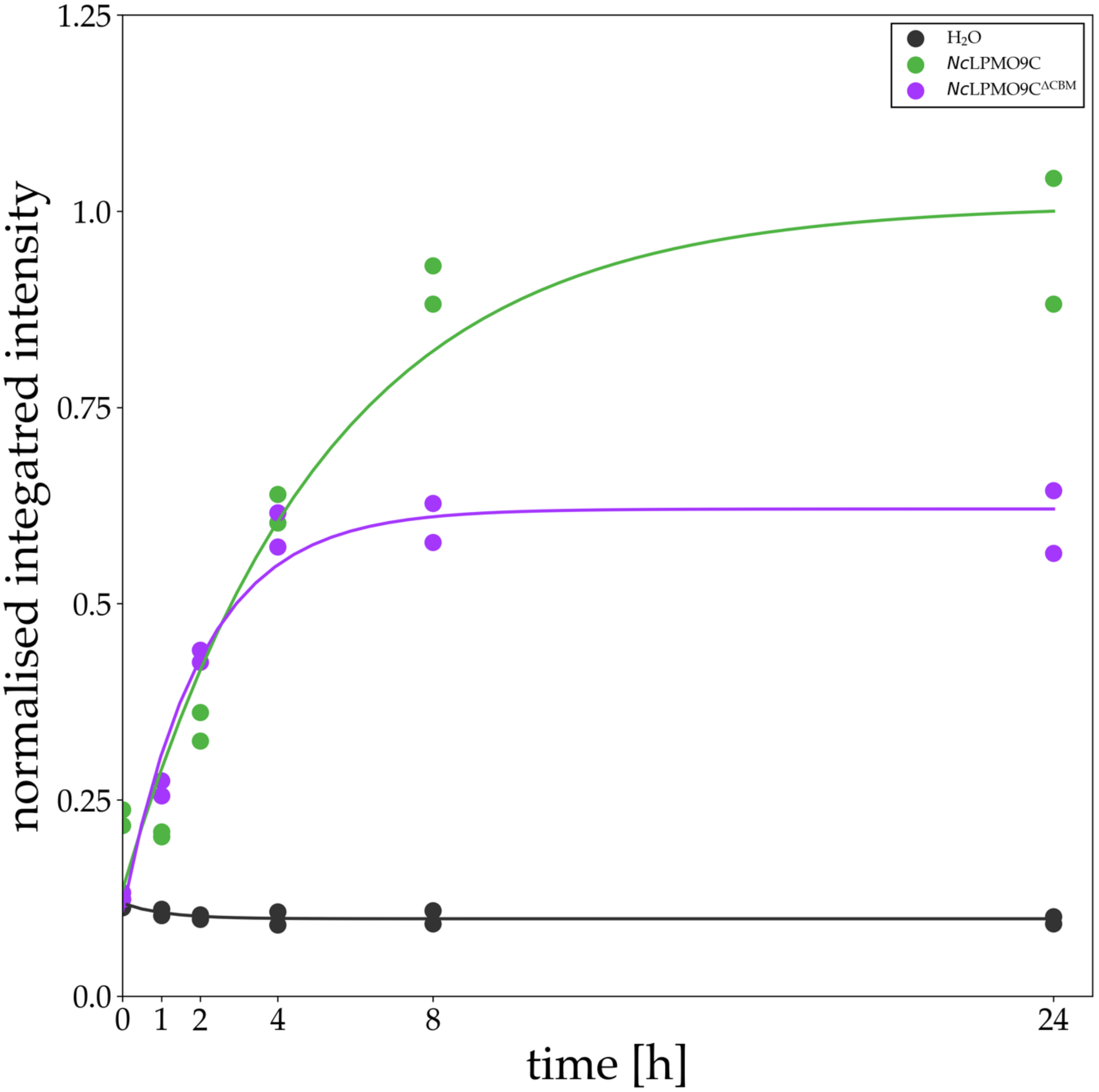

Figure S32. Normalized, integrated Elution window 3 over the conversion time. Curves are normalised to the maximum of the *Nc*LPMO9C curve. The curves are fitted to the data points using the following function: $y = ae^{-bx} + c$.

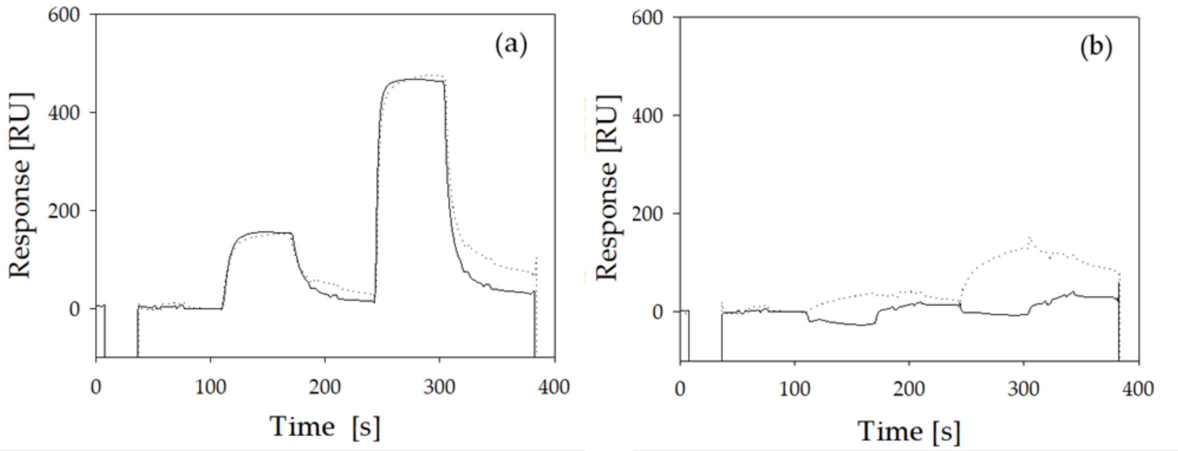

Figure S33. Sensograms of LPMO binding to cellulose surface in its oxidized and reduced state. The binding behavior of *Nc*LPMO9C (a) and *Nc*LPMO9C$^{\Delta CBM}$ (b) injected in two different concentrations (400 nM and 1600 nM) to the cellulosic substrate was recorded in the presence of oxygen (black line) and in the absence of oxygen supplemented with 10 mM gallic acid as reductant (dotted line). Sensograms were adjusted to the same response at the begin of the first injection.

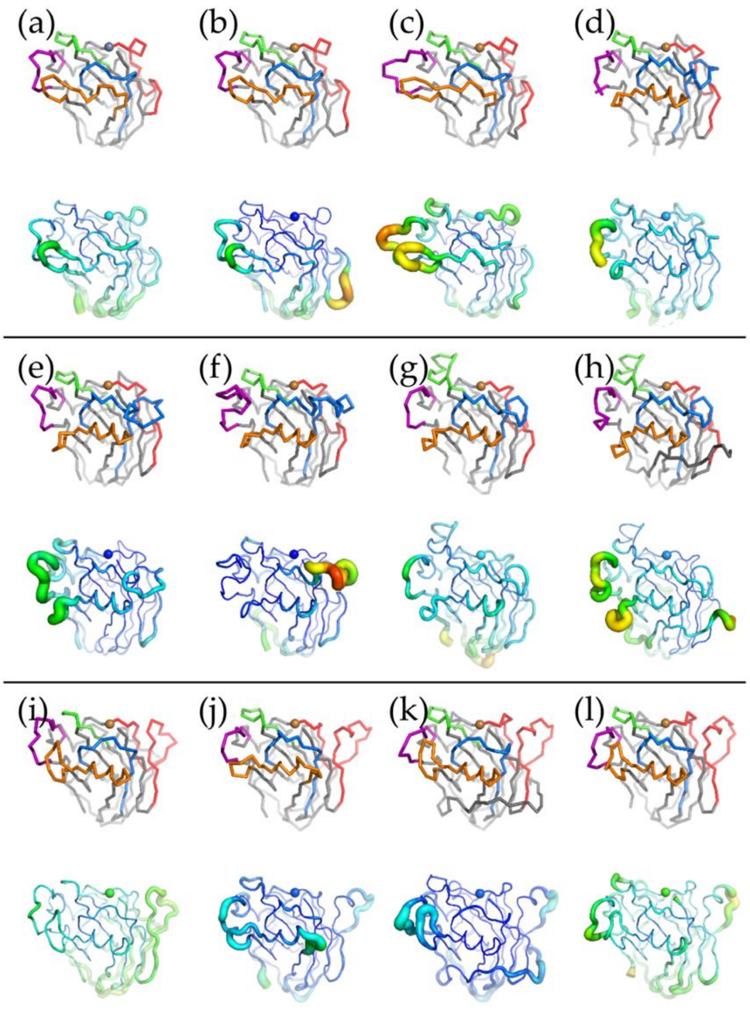

Figure S34. Representation of the mobility of the amino acid segment around the LPMO catalytic sites. Crystal structures of (a) TtLPMO9E (GeneBank: CP_003657366.1; PDB: 3EII), (b) *Nc*LPMO9F (Genebank: CAD70347.1; PDB: 4QI8), (c)PcLPMO9D (Genebank: BAL43430.1; PDB: 4B5Q), (d) *Nc*LPMO9A (GeneBank: EAA30263.1; PDB: 5FOH), (e) *Nc*LPMO9D (GeneBank: CAD21296.1; PDB: 4EIR), (f) *Nc*LPMO9C (GeneBank: EAA36362.1; PDB: 4D7U), (g) CvAA9A (GeneBank: AST24379.1; PDB: 5NLT), (h) LsAA9A (GeneBank: ALN96977.1; PDB: 5ACF), (i) HiLPMO9B (GeneBank: ETW87087.1; PDB: 5NNS), (j) *Nc*LPMO9M (GeneBank: EAA33178.2: PDB: 4EIS), (k) TrLPMO9A (GeneBank: CAA71999.1; PDB: 5O2W), (l) TaLPMO9A (GeneBank: ACS05720.1; PDB: 2YET). Top row: ribbon in gray and segments 1-5 in red, blue, orange, green, and magenta, respectively. Bottom row: flexibility indicated via „b-factor putty", that is, from blue via green to red and from narrow to wide tubes corresponds to low to high flexibility of the respective regions.

Table S1. Definitions of segments around the catalytic site of *Nc*LPMOs. The segment definitions for *Nc*LPMO9C$^{\Delta CBM}$ and *Nc*LPMO9C$^{\Delta Seg2,\ \Delta CBM}$ are identical to *Nc*LPMO9C and *Nc*LPMO9$^{\Delta Seg2}$, respectively.

**Enzymes**	**Segments**				
	**Seg1**	**Seg2**	**Seg3**	**Seg4**	**Seg5**
*Nc*LPMO9F	A15-G30	I62-P75	P107-F123	H146-Y157	I192-Y204
*Nc*LPMO9C	G15-N27	V58-P86	L115-S132	H155-Y166	I199-Y217
*Nc*LPMO9M	Q12-G40	V71-P85	R118-N136	S161-Y171	V205-Y215
*Nc*LPMO9$^{\Delta Seg2}$	G15-N27	V58-P71	L100-S117	H140-Y151	I184-Y202

Table S2. Purification scheme of recombinantly produced LPMOs. Culture supernatants of *P. pastoris* fermentations have been purified by hydrophobic interaction chromatography (HIC), anion exchange chromatography (AIEX), and size exclusion chromatography (SEC). The total protein concentration was determined with the Bradford method except for the pools indicated with an "*", which were measured with the bicinchoninic acid (BCA) assay. To the previously published purification protocol of *Nc*LPMO9C [13] a SEC step was added. *Nc*LPMO9F was produced by Kittl *et al*.

Purification step	Total protein [g]	Yield [%]
***Nc*LPMO9$^{\Delta Seg2}$**		
Culture supernatant	6.02	100
HIC	0.84	14
AIEX	0.80	13
***Nc*LPMO9M**		
Culture supernatant	4.48	100
HIC	0.49	11
AIEX	0.32	7
***Nc*LPMO9C$^{\Delta CBM}$**		
Culture supernatant	6.72	100
HIC	3.70	55
AIEX	1.04	15
SEC*	0.28	4
***Nc*LPMO9C$^{\Delta Seg2, \Delta CBM}$**		
Culture supernatant	3.84	100
HIC	0.11	2.9
AIEX	0.10	2.5
***Nc*LPMO9C**		
Stock*	0.13	100
SEC*	0.01	8
***Nc*LPMO9F**		
Stock*	0.005	

Table S3. Elution windows defined to integrate detected products in HPAEC chromatograms used for RAC and CMC conversion experiments. Values are in minutes. DP = Degree of polymerization (e.g., cellobiose id DP2)

DP2	DP3	DP4	DP5	DP6	Total
[10.40, 11.00]	[13.60, 14.25]	[16.00, 17.00]	[18.50, 19.40]	[20.50, 21.36]	[4.90, 45.00]

Table S4. Quantification of cellodextrins in the supernatant released by 1.25 µM enzymes after a 24 h incubation on 2 mg/mL RAC or CMC in the presence of 1 mM ascorbic acid. The integration times of product elution windows are defined as described in **Supplementary Table S3**.

**Enzyme**	**Released Cellodextrins [µM]**					
	**DP2**	**DP3**	**DP4**	**DP5**	**DP6**	**Total**
**RAC + ascorbic acid**						
Control	6.6	0.0	0.0	0.0	0.0	6.6
*Nc*LPMO9F	26.2	10.7	10.3	17.2	12.8	77.2
*Nc*LPMO9C	88.2	77.9	26.4	4.2	5.2	201.9
*Nc*LPMO9C$^{\Delta CBM}$	20.4	8.6	9.0	5.1	3.6	46.7
*Nc*LPMO9$^{\Delta Seg2}$	12.8	0.0	0.0	1.5	0.0	14.4
*Nc*LPMO9C$^{\Delta Seg2, \Delta CBM}$	15.8	0.0	0.0	1.0	0.0	16.8
*Nc*LPMO9M	40.3	50.9	34.8	25.4	19.4	170.8
**CMC + ascorbic acid**						
Control	14.8	0.0	0.0	1.0	0.0	15.8
*Nc*LPMO9F	13.0	2.8	2.3	2.7	5.6	26.5
*Nc*LPMO9C	61.9	25.2	0.0	0.0	0.0	87.1
*Nc*LPMO9C$^{\Delta CBM}$	24.8	12.0	7.4	5.2	3.5	52.8
*Nc*LPMO9$^{\Delta Seg2}$	16.0	0.0	0.0	2.1	0.0	18.1
*Nc*LPMO9C$^{\Delta Seg2, \Delta CBM}$	15.7	0.0	0.0	2.1	0.0	17.8
*Nc*LPMO9M	17.8	2.1	3.2	3.2	2.0	28.4

Table S5. Quantification of cellodextrins in the supernatant released by 1.25 µM enzymes after a 24 h incubation on 2 mg/mL RAC or CMC in the absence of ascorbic acid. The integration of product elution windows are defined as described in **Supplementary Table S3**.

**Enzyme**	**Released Cellodextrins [µM]**					
	**DP2**	**DP3**	**DP4**	**DP5**	**DP6**	**Total**
**RAC, no ascorbic acid**						
Control	0.0	0.0	0.0	0.0	0.0	0.0
*Nc*LPMO9F	1.5	0.7	0.9	0.4	0.1	3.6
*Nc*LPMO9C	6.7	5.2	6.3	1.7	0.2	20.1
*Nc*LPMO9C$^{\Delta CBM}$	0.0	0.0	0.0	0.0	0.0	0.0
*Nc*LPMO9$^{\Delta Seg2}$	0.0	0.0	0.0	0.0	0.0	0.0
*Nc*LPMO9C$^{\Delta Seg2, \Delta CBM}$	0.0	0.0	0.0	0.0	0.0	0.0
*Nc*LPMO9M	0.0	0.0	0.0	0.0	0.0	0.0
**CMC, no ascorbic acid**						
Control	0.0	0.0	0.0	0.0	0.0	0.0
*Nc*LPMO9F	11.6	4.1	7.5	3.6	1.5	28.3
*Nc*LPMO9C	22.9	10.9	14.8	5.0	0.7	54.3
*Nc*LPMO9C$^{\Delta CBM}$	0.0	0.0	0.0	0.0	0.0	0.0
*Nc*LPMO9$^{\Delta Seg2}$	0.0	0.0	0.0	0.0	0.0	0.0
*Nc*LPMO9C$^{\Delta Seg2, \Delta CBM}$	0.0	0.0	0.0	0.0	0.0	0.0
*Nc*LPMO9M	0.0	0.0	0.0	0.0	0.0	0.0

Table S6. Elution windows used to integrate XG conversion products in HPSEC chromatograms. Values shown are in min. The upper boundaries were excluded from the integrations as indicated by the open bracket "[".

Elution window 1	Elution window 2	Elution window 3
[7.40, 11.00[	[11.00, 12.00[	[12.00, 14.75[

Table S7. Nucleotide sequences of primer pair used to generate *Nc*LPMO9C$^{\Delta CBM}$ and *Nc*LPMO9C$^{\Delta Ceg2,\ \Delta CBM}$.

Primer name	Sequence (5'→3')
P213X_F	GTCCAGCTTTGTTCACTTGTTAAGCTGGTGGTTC
P213X_R	CAAAGCTGGACCTGGAATTTGGTATGGCTTAC

Table S8. Statistics of the peptide mapping with liquid chromatography-electrospray ionization mass spectrometry (LC-ESI-MS). Results with 1 unique peptide have been removed from the table. Two human proteins have been removed. Rows that were showing the used protease were also deleted from the table. The likelihood that a peptide is a random match is indicated the log(e) score. E.g., the log(e) of the first row corresponds a 1 in 10419.3 chance that this result is a false positive. The coverage indicates sequence coverage.

	Coverage [%]	log(e) Score	Unique Peptides	UniProt ID
*Nc*LPMO9C	**53**	**-419.3**	**34**	***Nc*LPMO9C**
*Nc*LPMO9C$^{\Delta CBM}$	**88**	**-622.4**	**50**	***Nc*LPMO9C$^{\Delta CBM}$**
*Nc*LPMO9C$^{\Delta Seg2}$	**72**	**-668.6**	**53**	***Nc*LPMO9C$^{\Delta Seg2}$**
	45	-63.6	6	C4R8X7 (*P. pastoris*)
*Nc*LPMO9C$^{\Delta Seg2, \Delta CBM}$	**62**	**-570.3**	**47**	***Nc*LPMO9C$^{\Delta Seg2, \Delta CBM}$**
	60	-149.4	13	C4R8X7 (*P. pastoris*)
	48	-72.4	7	C4R300 (*P. pastoris*)
	36	-72.3	8	A0A1B2JFA7 (*P. pastoris*)
	33	-214.1	19	C4R9F6 (P.pastoris)
	25	-93.5	10	C4QW09 (*P. pastoris*)
	23	-68.6	8	C4R862 (*P. pastoris*)
	22	-70.9	7	A0A1B2J744 (*P. pastoris*)
	21	-65.4	7	C4QY07 (*P. pastoris*)
	21	-63.3	7	C4R7U0 (*P. pastoris*)
*Nc*LPMO9F	**71**	**-244.7**	**27**	***Nc*LPMO9F**
	71	-62	7	A0A1B2J759 (*P. pastoris*)
	68	-359.3	33	A0A1B2JHZ1 (*P. pastoris*)
	39	-163.5	18	A0A1B2JGG7 (*P. pastoris*)
	39	-41.4	6	A0A1B2JCS3 (*P. pastoris*)
	38	-67.5	11	A0A1B2JEK0 (*P. pastoris*)
	28	-44.2	6	A0A1B2JGF8 (*P. pastoris*)
	17	-40.2	6	A0A1B2JIZ2 (*P. pastoris*)
	17	-38.8	6	A0A1B2J5L5 (*P. pastoris*)
	10	-54.8	6	A0A1B2J5W9 (*P. pastoris*)
	8.8	-35.8	5	A0A1B2J841 (*P. pastoris*)
*Nc*LPMO9M	**84**	**-369**	**32**	***Nc*LPMO9M**

Supplementary References

1. Frommhagen, M.; Westphal, A.H.; van Berkel, W.J.H.; Kabel, M.A. Distinct substrate specificities and electron-donating systems of fungal lytic polysaccharide monooxygenases. *Front. Microbiol.* 2018, *9*, 1080.
2. Kabsch, W.; Sander, C. Dictionary of protein secondary structure: Pattern recognition of hydrogen-bonded and geometrical features. *Biopolymers* **1983**, *22*, 2577–2637.
3. Eichenberger, A.P.; Allison, J.R.; Dolenc, J.; Geerke, D.P.; Horta, B.A.C.; Meier, K.; Oostenbrink, C.; Schmid, N.; Steiner, D.; Wang, D.; et al. GROMOS++ software for the analysis of biomolecular simulation trajectories. *J. Chem. Theory Comput.* **2011**, *7*, 3379–3390.
4. Bertoni, M.; Kiefer, F.; Biasini, M.; Bordoli, L.; Schwede, T. Modeling protein quaternary structure of homo- and hetero-oligomers beyond binary interactions by homology. *Sci. Rep.* **2017**, *7*, 10480.
5. Benkert, P.; Biasini, M.; Schwede, T. Toward the estimation of the absolute quality of individual protein structure models. *Bioinformatics* **2011**, *27*, 343–350.
6. Guex, N.; Peitsch, M.C.; Schwede, T. Automated comparative protein structure modeling with SWISS-MODEL and Swiss-PdbViewer: A historical perspective. *Electrophoresis* **2009**, *30*, S162–S173.
7. Bienert, S.; Waterhouse, A.; de Beer, T.A.P.; Tauriello, G.; Studer, G.; Bordoli, L.; Schwede, T. The SWISS-MODEL Repository—new features and functionality. *Nucleic Acids Res.* **2017**, *45*, D313–D319.
8. Waterhouse, A.; Bertoni, M.; Bienert, S.; Studer, G.; Tauriello, G.; Gumienny, R.; Heer, F.T.; de Beer, T.A.P.; Rempfer, C.; Bordoli, L.; et al. SWISS-MODEL: homology modelling of protein structures and complexes. *Nucleic Acids Res.* **2018**, *46*, W296–W303.
9. Breslmayr, E.; Hanžek, M.; Hanrahan, A.; Leitner, C.; Kittl, R.; Šantek, B.; Oostenbrink, C.; Ludwig, R. A fast and sensitive activity assay for lytic polysaccharide monooxygenase. *Biotechnol. Biofuels* **2018**, *11*, 79.
10. Durowoju, I.B.; Bhandal, K.S.; Hu, J.; Carpick, B.; Kirkitadze, M. Differential scanning calorimetry - a method for assessing the thermal stability and conformation of protein antigen. *J. Vis. Exp.* **2017**, *121*, e55262.
11. Micsonai, A.; Wien, F.; Bulyáki, É.; Kun, J.; Moussong, É.; Lee, Y.-H.; Goto, Y.; Réfrégiers, M.; Kardos, J. BeStSel: a web server for accurate protein secondary structure prediction and fold recognition from the circular dichroism spectra. *Nucleic Acids Res.* **2018**, *46*, W315–W322.
12. Negri, L.H.; Vestri, C. peakutils: v1.1.0 2017.
13. Kittl, R.; Kracher, D.; Burgstaller, D.; Haltrich, D.; Ludwig, R. Production of four *Neurospora crassa* lytic polysaccharide monooxygenases in *Pichia pastoris* monitored by a fluorimetric assay. *Biotechnol. Biofuels* **2012**, *5*, 79.
